# Patient and public involvement (PPI) reporting in maternal and neonatal clinical trials: an exploratory review

**DOI:** 10.1186/s13063-026-09580-z

**Published:** 2026-03-06

**Authors:** Kathleen Hannon, Déirdre Daly, Valerie Smith

**Affiliations:** 1 School of Nursing and Midwifery, Trinity College Dublin, 24 D’Olier Street, Dublin, D02 T283 Ireland; 2 Health Research Board—Trials Methodology Research Network (HRB-TMRN), Galway, Ireland; 3 Trinity Centre for Maternal Care Research (TCMCR), Trinity College Dublin, Dublin, D02 T283 Ireland; 4 School of Nursing, Midwifery and Health Systems, University College Dublin, Health Sciences Centre, 4 Stillorgan Rd, Belfield, Dublin Ireland

**Keywords:** Patient and public involvement, Neonatal trials, Maternal trials, GRIPP2 checklist, Clinical trials methodology

## Abstract

**Background:**

Patient and public involvement (PPI) is increasingly a research priority encouraged by health research funders. It is difficult to know, however, how prevalent PPI is within research. The aim of this review was to gain a contemporary understanding of the frequency and types of PPI being reported in maternal and neonatal clinical trial reports, and if an increase in PPI reporting was evident over time.

**Methods:**

An exploratory review of maternal and neonatal trial reports published in nine healthcare journals between 2017 and 2022 was undertaken. A search was conducted for eligible trial reports in each journal using SCOPUS, in addition to a manual search of each journal’s archive. Once an eligible trial report was identified, a search was conducted for the trial’s associated protocol. Both trial documents were reviewed for any information on PPI activity. Descriptive statistics of the included trials’ characteristics were produced.

**Results:**

Three hundred and fifty-two trial reports, along with the associated trial protocols for 170 trial reports, were identified and included in the analysis. Of these, 48 trials (14%) reported PPI, either within the main trial record (*n* = 40) or solely in the trial protocol (*n* = 8). Twenty (42%) of these trials were conducted in the UK. Thirty-six trials reported PPI contributors in trial design and planning, 29 trials reported PPI during the running of the trial, and 20 trials involved contributors in trial analysis and dissemination. There was no sustained increase in PPI reporting over the included timeframe.

**Conclusions:**

There is minimal reporting of PPI in maternal and neonatal clinical trials, with wide variations in the depth of information provided. PPI reporting guidelines in academic journals may be beneficial in prompting researchers to provide PPI information and to raise awareness of the profile of PPI in maternal and neonatal trial research.

**Registration number:**

The review was not registered.

**Supplementary Information:**

The online version contains supplementary material available at 10.1186/s13063-026-09580-z.

## Background

There has been a growing emphasis on incorporating patient and public involvement (PPI) into health research over the past decade, with health research funders in Ireland and the United Kingdom (UK) requesting researchers to incorporate PPI into their research proposals [1, 2]. A considerable amount of the current literature on PPI has emerged from the UK [3], where PPI has been incorporated into their national research policies and governance since the early 2000 s [4, 5]. PPI is generally understood as involving patient and other members of the public, known as PPI contributors or PPI partners, *in* designing and conducting research as opposed to conducting research *on* these individuals or groups [1]. In trials, PPI contributors are typically involved as members of the trial’s steering committee [6] or asked to review trial documents, such as participant information leaflets (PILs) [7]. While PPI is regarded as particularly important to implement within the conduct of clinical trials [8, 9], there is limited evidence-based guidance available on how best to conduct PPI within a trial [7]. Several barriers to implementing PPI have been identified, such as difficulties recruiting PPI contributors, lack of funding to reimburse PPI contributors, a lack of time to conduct PPI activities, and PPI contributors not feeling listened to or valued within the research process [7, 10].

Issues concerning inconsistency or a lack of reporting in research about when, how, and why PPI is conducted have been raised previously [11, 12]. Poor reporting of PPI limits the available information on how PPI is being implemented in research and makes it difficult to assess the extent and impact of PPI [13]. There have been efforts to improve the extent and quality of PPI reporting; for instance, the GRIPP2 checklist (Guidance for Reporting Involvement of Patients and the Public) was developed to provide guidance to researchers when reporting PPI [14].

As in many areas of healthcare, PPI is regarded as a means to improve the health and health outcomes of pregnant and postpartum women and their babies, by helping to shape research that is relevant and acceptable to the needs of the target population [15–18]. For instance, there is evidence of maternity health researchers using innovative and creative approaches in order to involve women from under-represented groups in research [19, 20], which has been viewed as having the potential to reduce health inequalities [20]. As such, PPI has become a priority in maternal and neonatal research [16, 20, 21]. It remains unknown, however, if developments in involving members of the public are translating to PPI reporting in maternal and neonatal trial reports.

To gain a contemporary understanding of the frequency of PPI reporting in published maternal and neonatal clinical trial reports, the types of PPI activities being conducted, and who is being involved as PPI contributors, we undertook an exploratory review of PPI reporting in maternal and neonatal trials published over a 6-year period, between 2017 and 2022. In undertaking the review, we were interested in determining if PPI reporting had increased over time and, for journals that had PPI reporting guidelines, whether authors provided reasons for not conducting PPI (if this was the case). These explanations may provide some insight into the reasons why PPI was not implemented, and potential barriers to conducting PPI.

## Materials and methods

### Inclusion criteria

Reports of clinical trials, either academic or industry-led, in maternal and neonatal health, published in nine selected journals from 2017 to 2022, and the trial’s protocol, were eligible for inclusion. The 6-year time frame was chosen to offer contemporary insight as well as providing a duration that would facilitate a sufficiently comprehensive review. In addition, as the GRIPP2 checklist was published in 2017 [14], we wished to see if the checklist was being adhered to when authors of trial reports were reporting PPI. Protocols that were publicly available via trial registries, as supplementary material of a published trial report or published as a stand-alone journal publication, were eligible for inclusion. Trials that did not provide an assigned trial registry number, or if evidence of registration could not be found elsewhere, were excluded. Systematic reviews of trial data, short reports, secondary analyses papers, and reports of trials that were actively recruiting or currently in progress were also excluded.

For this review, we considered PPI to be any information provided in the manuscript that indicated that lay individuals—which could include, for example, charity representatives, parents, and other members of the public—were involved at any stage of a trial. This could include information that was described by the trial manuscript authors explicitly as PPI, such as information provided under a ‘*patient and public involvement statement*’ within a trial manuscript. This also included trial manuscripts where PPI was indirectly referenced, such as a listed co-author whose affiliation information identified them as a ‘non-professional researcher’ ( [22], p. 2–3).

### Search strategy

Our decision-making on which journals to include was pragmatically driven. Two journals were initially chosen as they had implemented PPI reporting guidelines at the time of the review, had a high impact factor, and regularly published trial reports of relevance to maternal and neonatal health; the *British Medical Journal* (*BMJ*) implemented their guidelines in 2014 [23], and the *British Journal of Obstetrics and Gynaecology* (*BJOG*)’s PPI reporting guidelines were introduced in 2018 [24]. We wished to compare these journals to other journals with similar fields of interest.

i) General health journals: *The Lancet* and the *New England Journal of Medicine* (*NEJM*), in addition to the already included *BMJ*, were previously identified by trials methodology researchers involved in a similar study to this current review as ‘key journals for publishing trials’ ( [25] p. 3).
ii) Maternal health research journals: A search was conducted of high-impact journals listed on Clarivate. *BJOG* and *Obstetrics & Gynecology* were listed in the top seven journals, as was the *American Journal of Obstetrics and Gynecology* (*AJOG*) which was used to conduct a pilot search as part of planning this review. *BJOG* and *Obstetrics & Gynecology* were chosen based on our familiarity with these journals in comparison to the other listed journals. We then decided to include *BMC Pregnancy and Childbirth* as it is an open-access journal, and we had prior knowledge that it regularly published relevant trial reports.
iii) Neonatal health journals: *Archives in Disease—Fetal & Neonatal Edition* and *Pediatrics* were listed in the top eight high-impact journals in the field of paediatric journals listed on Clarivate. Some of the other high-impact journals were not considered for inclusion as they were too specific or were not focused on the cohort of interest (e.g. *Child and Adolescent Psychiatry and Mental Health* and *Journal of Adolescent Health*). Finally, *Neonatology* was chosen as it was one of the top journals that focused specifically on neonates.

*NEJM* request that authors provide the trial protocol as supplementary material when submitting a trial report [26], which would likely increase the probability of protocol availability for trials included in this review that were published in that journal.

#### Search of journals

A search strategy was developed to search the *SCOPUS* database for each included journal (Supplementary file 1). The SCOPUS search results were downloaded as Excel spreadsheets for each journal and all publications screened by title and abstract. Potentially eligible reports were accessed, and the full texts were screened.

All nine of the journals’ websites were also searched manually. This entailed accessing the online archive of each journal and searching through each issue published between January 2017 and December 2022 inclusive. The Table of Contents for each published issue was screened for potentially relevant trial reports. Potentially relevant trial reports were accessed, and the title and abstract were screened to determine if the report was eligible for inclusion. If a trial report met the inclusion criteria, the reference for the trial report was recorded in an Excel database for full-text screening and data extraction.

The results from the SCOPUS and manual searches were compared to ensure all potentially relevant reports were included. All included trial reports were searched for a reference to a published trial protocol. If it was not located in the trial report, the trial registration number was located on the relevant trial registry to check for a published trial protocol. Both the report and published trial protocol, if available, were reviewed in full for any information relating to PPI. All data that appeared related to PPI were extracted verbatim onto a purposively pre-designed data extraction form (Supplementary file 2).

Data extracted from each report and/or protocol included author information and affiliations, country of origin, health condition or condition of interest, the type and purpose of the intervention under evaluation, and details related to funding. Any information relating to PPI was extracted, such as the type of contributor involved, the number of PPI contributors reported, the trial stage at which PPI occurred, and if any reference to training or reimbursement for PPI contributors’ input was provided. No assessment of methodological quality was conducted on included reports as this was not the focus of the current review. All trial reports that were identified as eligible, irrespective of quality, were included.

The lead author reviewed and extracted the data from the identified trials. In the event of trial reports where it was unclear if PPI had occurred or not, this was discussed among the reviewers until a decision was reached.

### Patient and public involvement in this review

Although PPI contributors were not involved directly in the design or conduct of this review, the review was conducted as part of a wider study on PPI in maternal and neonatal trials, and which involves PPI contributors at different stages, including a member of the overall study Advisory Group.

## Results

### Screening

**Fig. 1:**
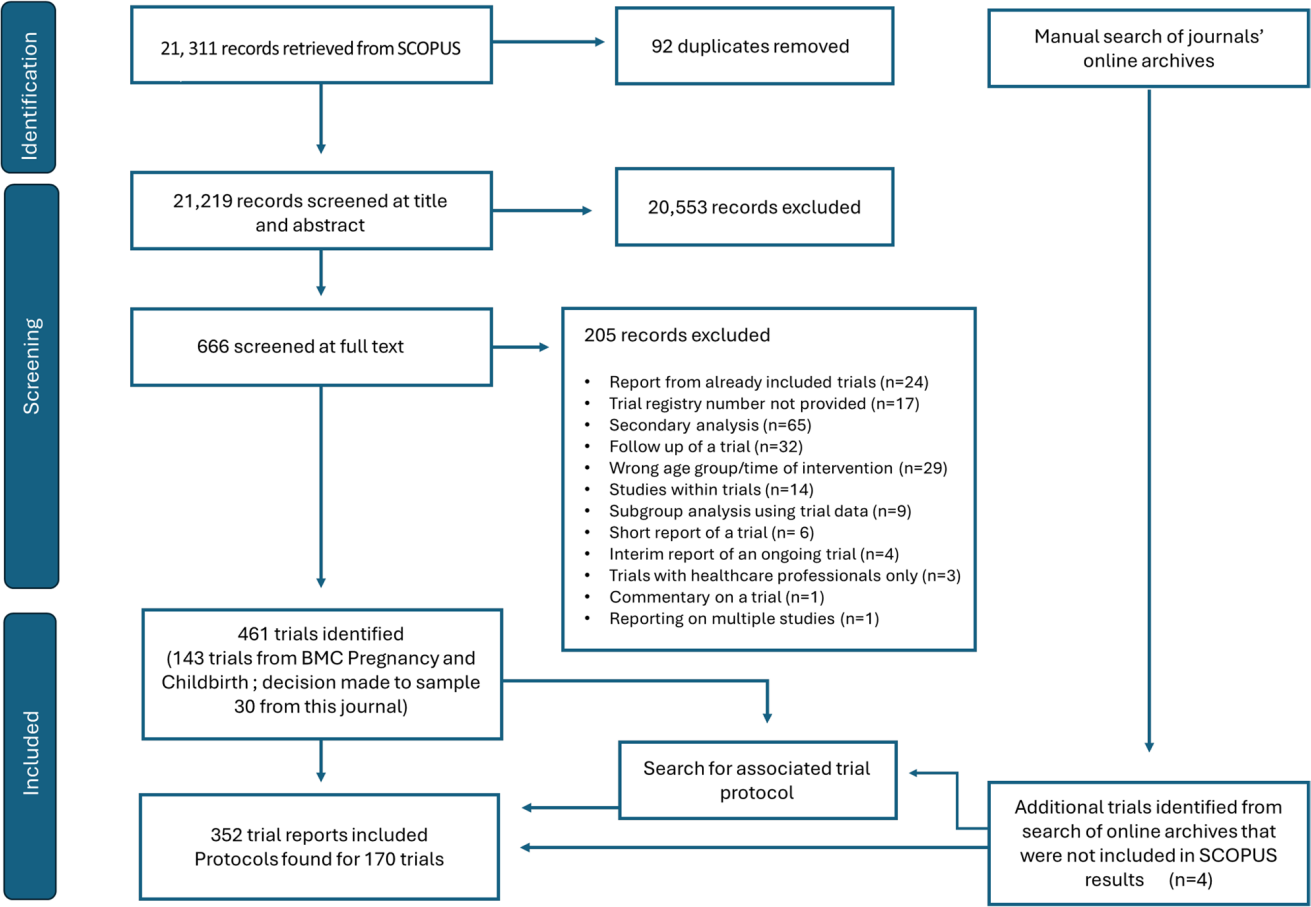
Screening process to identify trial reports and associated trial protocols

A total of 21,311 records were identified using SCOPUS (Fig. 1). Following removal of duplicates (*n* = 92), 21,219 records were screened by title and abstract and 20,553 were excluded. A total of 666 were screened at full text and 205 records were excluded, leaving 461 eligible reports. Of these, 143 (31%) were published in *BMC Pregnancy and Childbirth*. For balance and volume management, a decision was taken to only include 30 trial reports from this journal, consisting of the first five trial reports published in each included year. Forty-one of these trial records were excluded because 24 were economic analyses or follow-up studies of already included trial reports, and the trial registry numbers could not be located for 17 records. The manual search results identified four additional trials that had not been captured by the SCOPUS search. Thus, 352 trial reports were included for purposes of analyses, with associated trial protocols identified for 170 trials; published trial protocols were not found for the remaining 182 trial reports. A total of 53 protocols were published as supplementary files of the main trial report and 29 trials had two protocols available each; as a supplement to the main trial record *and* as a separate publication. Supplementary file 3 provides a list of all included trial reports and associated trial protocols.

A summary of the characteristics of the included trials is presented in Table 1 (see Supplementary file 4 for more information). Of the 352 trials identified, the majority were reports of randomised trials (*n* = 330) with the remainder of reports being quasi-experimental trials or non-randomised controlled or open label trials. Forty-six were multinational trials, and 306 were single-country trials, conducted in 42 locations. A total of 119 were reports of neonatal trials and 233 were reports of pregnancy, intrapartum or early postpartum trials. A total of 109 trials (31%) were investigating drug interventions while 49% (*n* = 174) trials were for the purpose of treatment of a condition or illness.

**Table 1:** Characteristics of all included trials (*n* = 352)

PPI status			Total (*n* = 352) *n* (%)
Reported PPI			48 (14%)
Stated PPI did not occur			28 (8%)
No information on PPI activity provided			272 (77%)
Unclear on PPI status			4 (1%)
Trial population			
Neonatal trial			119 (34%)
Maternal trial			233 (66%)
Antepartum intervention			141 (40%)
Intrapartum intervention			63 (18%)
Postpartum intervention			29 (8%)
Trial design			
Randomised trials			330 (94%)
Non-randomised trials			19 (5%)
Quasi-experimental trials			3 (<1%)
Type of intervention tested	Maternal trials (*n* = 233)	Neonatal trials (*n* = 119)	Total trials (*n* = 352)
Drug	81 (35%)	28 (24%)	109 (31%)
Other	43 (18%)	35 (29%)	78 (22%)
Device	29 (12%)	27 (23%)	56 (16%)
Behavioural	36 (15%)	5 (4%)	41 (12%)
Procedure	23 (10%)	17 (14%)	40 (11%)
Testing two or more interventions (e.g. comparing a device to a drug intervention)	12 (5%)	4 (3%)	16 (5%)
Dietary supplement	4 (2%)	2 (2%)	6 (2%)
Diagnostic test	4 (2%)	–	4 (1%)
Vaccine	1 (<1%)	1 (<1%)	2 (1%)
Purpose of the trial	Maternal trials (*n* = 233)	Neonatal trials (*n* = 119)	Total trials (*n* = 352)
Treatment	103 (44%)	71 (60%)	174 (49%)
Prevention	87 (37%)	25 (21%)	112 (32%)
Diagnostic/screening	13 (6%)	5 (4%)	18 (5%)
Supportive care	7 (3%)	6 (5%)	13 (4%)
Health services research	6 (3%)	1 (<1%)	7 (2%)
Other*	17 (7%)	11 (9%)	28 (8%)

^*^Maternal trials: ‘Other’ category includes 13 described as ‘other’ intervention on the trial’s registry, 2 QoL interventions, 1 educational intervention, and 1 basic science intervention. Neonatal trials: ‘Other’ category includes 1 basic science intervention, 1 device feasibility study, 1 QoL, and 8 described as ‘Other’ on the trial’s registry

### PPI reporting in included trials

Of the 352 trials, 48 (14%) mentioned PPI activity in the main trial record (*n* = 40), or reported PPI within the trial’s associated protocol, which was available as supplementary material to the main trial record (*n *= 13) or published separately (*n* = 25). A total of 23 trials reported PPI in both the main trial report *and* the trial’s associated protocol. A total of eight trials reported PPI only in the trial's protocol. Twenty-eight trials (8%) explicitly stated that they did not conduct PPI at any stage of the trial, with the majority of trials (77%, *n* = 272) providing no details as to whether PPI had occurred. PPI reporting in four trial reports was categorised as ‘unclear reporting’ because the information was insufficient to determine if it constituted PPI. For example, one of these trials mentioned the involvement of community representatives in a Trial Advisory Group [27]. However, in the list of Advisory Committee members provided, only healthcare professionals appear to be listed [28].

One trial reporting PPI in the main trial report provided conflicting information about the extent of involvement in the trial protocol. The trial protocol stated that while PPI contributors were not involved in trial design or conduct, the results would be disseminated through established patient groups [29]. However, the main trial report stated that a panel of maternity service users *were* involved in the trial’s development, with particular input into the PIL and trial questionnaire development [30]. 

### Trials reporting ‘no involvement’ of patients or the public

Eight of the 28 trials that stated that no PPI had occurred provided reasons for non-involvement and 21 did not. Explanations included that PPI was not common practice or required at the time the trial began (*n* = 4, 14%), or that the trial outcome (an economic evaluation) did not require public involvement (*n* = 1). Other trialists reported that formal research with participants and/or healthcare professionals was conducted in preparation for the trial (*n* = 3).

### PPI reporting in main trial reports over time period and by journal (*n* = 354)

During 2017 to 2022, the *BMJ* (50%) and *The Lancet* (38%) had the highest reporting of PPI in maternal and neonatal trial reports, followed by *BJOG* (28%). *Obstetrics & Gynecology* did not appear to report PPI in any of the 82 trial reports from this journal (Table 2).

**Table 2:** Frequency and source of PPI reporting in maternal and neonatal trials (2017–2022)

Journal	No. of trials published	Reported PPI in trial record *n* (%)	Reported in trial protocol only *n* (%)	Unclear reporting *n* (%)	Reported no involvement *n* (%)	Did not report any information on PPI *n* (%)
*The BMJ*	14	7 (50%)		1 (7%)	6 (43%)	–
*The Lancet*	32	12 (38%)*	1 (3%)	1 (3%)	–	18 (56%)
*NEJM*	42	8 (19%)**		1 (2%)	–	33 (79%)
*BJOG*	53	15 (28%)		–	21 (40%)	17 (32%)
*Obstetrics & Gynecology*	82	–	–	1 (1%)	–	81 (99%)
*BMC Pregnancy & Childbirth*	30***	1 (3%)	–	–	1 (3%)	28 (93%)
*Pediatrics*	26	1 (4%)	–	–	–	25 (96%)
*Archives in Disease—Fetal & Neonatal Edition*	48	2 (4%)	–	–	–	46 (96%)
*Neonatology*	25	1 (4%)		–	–	24 (96%)
Total trials	352	47 (13%)	1 (<1%)	4 (1%)	28 (8%)	272 (77%)
Total trials reporting PPI		48 (14%)				

^*^The Lancet—two trials reported PPI solely within the protocol provided as supplementary material of the main trial record

^**^NEJM—five trials reported PPI solely within the protocol provided as supplementary material of the main trial record

^***^A total of 143 potentially eligible trial reports were published in *BMC Pregnancy and Childbirth* from 2017 to 2022—a sample of 30 reports were included in this review

Exploring PPI reporting over time found that PPI reporting, overall, increased from a low of 3% (*n* = 2) of trials in 2017 to 21% (*n* = 13) of all trials analysed in this review that were published in 2019. After 2019, however, there was a slight decrease in PPI reporting to 16% (*n* = 10) in 2020, 15% (*n* = 8) in 2021, and a slight increase to 17% (*n* = 8) in 2022. When examining each individual journal’s reporting by year of publication, there was no indication that the proportion of PPI reporting had steadily increased over time, other than in *BJOG* (Supplementary file 5).

### Reporting of PPI contributors and activities

Tables 3 and 4 present the characteristics of the 48 trials that reported PPI. A range of conditions were investigated in the 36 maternal trials (22 discrete conditions) and 12 neonatal trials (11 discrete conditions) (Supplementary file 6). Miscarriage management and prevention (*n* = 4) and preeclampsia (*n* = 4) were the most frequently investigated maternal health conditions that reported PPI, and neonatal respiratory conditions were the most frequently investigated neonatal health condition (*n* = 2). Twenty trials (42%) reporting PPI were conducted in the UK, followed by 11 multinational trials (23%). Of these 11 multinational trials, six included the UK as one of their locations. The remaining 17 trials reporting PPI were conducted in nine different countries. Sixteen trials (33%) were funded by the UK’s National Institute for Health and Care Research (NIHR), who require funding recipients to include PPI.

**Table 3:** Characteristics of trials reporting PPI (*n* = 48)

			Total (*n* = 48) *n* (%)
Type of trial			
Maternal trial			36 (75%)
Neonatal trial			12 (25%)
Type of intervention	Maternal trials (*n* = 36) *n* (%)	Neonatal trials (*n* = 12) *n* (%)	Total (*n* = 48) *n* (%)
Drug	11 (31%)	3 (25%)	14 (29%)
Other*	9 (25%)	5 (42%)	14 (29%)
Procedure	7 (19%)	1 (8%)	8 (17%)
Behavioural	4 (11%)	2 (17%)	6 (13%)
Device	3 (8%)	1 (8%)	4 (8%)
Testing two or more interventions	1 (3%)	–	1 (2%)
Dietary supplement	1 (3%)	–	1 (2%)
Vaccine	–	–	
Diagnostic test	–	–	
Purpose	Maternal trials (*n* = 36) *n* (%)	Neonatal trials (*n* = 12) *n* (%)	Total (*n* = 48) *n* (%)
Treatment	14 (39%)	8 (67%)	22 (46%)
Prevention	16 (44%)	4 (33%)	20 (42%)
Diagnostic/screening	4 (11%)	–	4 (8%)
Supportive care	–	–	–
Health services research	1 (3%)	–	1 (2%)
Other	1 (3%)	–	1 (2%)
Location			Total (*n* = 48) *n* (%)
UK			20 (42%)
Multinational trials			11 (23%)
Australia			4 (8%)
Netherlands			4 (8%)
Ireland			3 (6%)
France			1 (2%)
India			1 (2%)
Sweden			1 (2%)
Denmark			1 (2%)
South Africa			1 (2%)
Hong Kong, China			1 (2%)
PPI reporting by year of publication—main trial report and supplement protocol only			*n* (%)
2017 (*n* = 70)			2 (3%)
2018 (*n *= 54)			7 (13%)
2019 (*n *= 63)			13 (21%)
2020 (*n *= 61)			10 (16%)
2021 (*n *= 53)			8 (15%)
2022 (*n *= 51)			8 (17%)
Location of PPI reporting			Total (*n* = 48) *n* (%)
Reported PPI in protocol			30 (63%)
Reported PPI in protocol *only*			8 (17%) (%)
PPI in main trial report			40 (83%)
Co-authors/authors’ information			10 (21%)
Methods section			26 (54%)
Discussion section			2 (4%)
Acknowledgements/contributors' information			11 (24%)
Funding information			1 (2%)
Supplementary protocol			13 (27%)
Description of involved PPI contributors			Total (*n* = 48) *n* (%)
Charities, advocacy, or other non-profit organisations			24 (50%)
Maternity service users and families			15 (31%)
PPI contributor			9 (19%)
Patient representatives			7 (15%)
Former trial/research participants or their guardians			3 (6%)
Community representatives/community leaders			3 (6%)
Lay member of ethics committee			2 (4%)
			Total (*n* = 48) *n* (%)
Reported training provided to PPI contributors			0% (0)
Reported reimbursement			0% (0)

^*^Maternal trials: ‘Other’ category includes six described as ‘other’ intervention on the trial’s registry, 1 educational intervention, and 1 described as 'prevention'. Neonatal trials: ‘Other’ category includes five trials described as ‘Other’ on the trial’s registry

**Table 4:** Details of trials that reported PPI (*n* = 48)

Journal Author	Country	Funder	Maternal trial/neonatal trial	Health condition/area under evaluation	Location of PPI reporting	PPI activity	PPI contributors
*BMJ*							
Tappin et al*.* [31]	UK	- Cancer Research UK - Chief Scientist Office (Scotland) - HSC Public Health Agency, Northern Ireland (NI) - Health and Social Care R&D Division (NI) - Northern Ireland Chest Heart and Stroke - Scottish Cot Death Trust - Lullaby Trust	Maternal	Smoking cessation in pregnancy	Methods. Protocol [32].	- Trial planning	- Two previous smokers who participated in a feasibility trial
						- Trial steering committee (TSC)	- Patient representative
Cluver et al. [33]	South Africa	- Mercy Health Foundation - Peter Joseph Pappas Preeclampsia Foundation - South African Medical Research Council	Maternal	Preeclampsia	Methods	- Reviewed trial manuscript	- Member of the public
						- Developed dissemination plan	- Knowledge users
Hayes-Ryan et al*.* [34]	Ireland	Health Research Board Mother and Baby Clinical Trial Network Collaborative Ireland	Maternal	Preeclampsia	Methods. Protocol [35].	- Reviewed trial protocol	- Lay representatives on research ethics committees
						- TSC	- Public representatives
Henrichs et al*.* [36]	Netherlands	Netherlands Organisation for Health Research and Development (ZonMw)	Maternal	Perinatal morbidity and mortality	Methods	- Contributed to developing the grant proposal and gave feedback on trial design, partook in discussions concerning trial findings	- Patient representative
						- Communicating trial findings to the public	- Client organisations (unnamed)
Keulen et al*.* [37]	Netherlands	- ZonMw	Maternal	Post-term birth, prolonged pregnancy	Methods	- Plan to conduct future PPI: developing public facing material and documents	- Patient representatives
Adnan et al*.* [38]	Ireland	- Trinity College Dublin - Coombe Women and Infants University Hospital	Maternal	Postpartum haemorrhage	Methods	- Feedback on design	- Women in pilot trial
						- Feedback on patient information leaflet (PIL)	- Lay members of the institutional research ethics committee
*The Epidural and Position Trial Collaborative * [39]	UK	National Institute for Health Research (NIHR)	Maternal	Spontaneous vaginal birth	Methods	- Trial planning and trial meetings	- PPI co-investigator
						- Grant application	
						- Trial protocol development	
						- Trial report	
						- Developing PILs	
						- Disseminating findings, including producing a summary report	
*The Lancet*							
The INFANT Collaborative Group [40]	Multinational	NIHR	Maternal	Foetal monitoring	Co-authors of trial report (not identified as PPI contributors); supplementary protocol. Reported in published protocol [41].	- Protocol development	- Two co-investigators—National Childbirth Trust (NCT)
						- Trial running and trial management - Co-authors of trial report	
Norman et al*.* [42]	Multinational	- Chief Scientist Office, Scottish Government - Tommy’s - Sands	Maternal	Stillbirth	Acknowledgements. Protocol [43]	- TSC	- Lay member
Wilson et al*.* [44]	UK	- NIHR	Maternal	Pain management	Discussion, role of the funding source. Protocol [45]	- TSC	- Two PRIME members (Public and Researchers Involvement in Maternal and Early pregnancy group)
						- Reviewing information resources for participants	- PRIME group members
						- Trial design	
Hartley et al*.* [46]	UK	- NIHR - Wellcome Trust	Neonatal	Pain management	Acknowledgements. Protocol [47]	- Trial design - Trial promotion - Development of trial material—participant information resources - Trial conduct	- SSNAP (Supporting Sick Newborn and their Parents) charity representative - PPI contributor
						- TSC	- PPI contributor
Griffiths et al*.* [48]	UK	- NIHR	Neonatal	Infection	Authorship. Protocol [49]	- Trial co-investigator - Co-author of trial report and protocol	- BLISS representative
						- Trial development and delivery	- Infant and family representatives mainly recruited through BLISS
Knight et al. [50]	UK	- NIHR	Maternal	Surgical/operative birth infection	Supplementary protocol and in published protocol [51]	- TSC	- PPI representative from NCT
						- Trial Collaborative Group - Involved in producing the trial report	- Lay member—PRIME group
Chappell et al*.* [52]	UK	- NIHR	Maternal	Intrahepatic cholestasis of pregnancy	Authorship. Protocol [53]	- Co-author of trial manuscript and protocol	- ICP Support representative
						- Co-Investigator Group	
						- TSC	- PPI representative
Chappell, Brocklehurst et al*.* [54]	UK	- NIHR	Maternal	Preeclampsia	Authorship, acknowledgements. Protocol [55]	- TSC	- Action On Pre-Eclampsia (APEC) representative/ lay member
						- Co-Investigators Group - Co-author of trial report and protocol	- APEC representative
Chu et al*.* [56]	UK	- NIHR	Maternal	Miscarriage	Authorship; discussion. Protocol [57]	- Consulted about the trial’s primary outcome	- Women diagnosed with miscarriage
						- TSC	- Two PPI contributors: Miscarriage Association representative and one PPI representative
						- Co-authors of trial report	- Tommy’s charity representative - Miscarriage Association representative
Caeymaex et al*.* [58]	France	- Solidarity and Health Ministry (France)	Neonatal	NICU adverse events	Authorship, was confirmed as PPI due to the trial protocol [59]	- Co-author of main trial report - Dissemination of findings	- Association SOS Prema representative
Edqvist et al*.* [60]	Sweden	- Swedish Research Council for Health, Working Life and Welfare - Jan Hains Research Foundation - Skane County Council’s Research and Development Foundation	Maternal	Birth injury prevention	Acknowledgements. Protocol [61]	- Trial design and acceptability	- User representative
						- Review of trial questionnaires	- Focus groups with women
Hodgetts Morton, Toozs-Hobson et al*.* [62]	UK	NIHR	Maternal	Miscarriage prevention	Protocol [63]	- TSC	- Two PPI representatives
Chalmers et al*.* [64]	UK	NIHR	Neonatal	Eczema	Acknowledgements; supplementary protocol and published protocol [65]	- Trial design	- Patients
						- TSC’	- PPI representative
*NEJM*							
Lissauer et al*.* [66]	Multinational	- Medical Research Council (UK) and others	Maternal	Miscarriage	Supplementary material (protocol)	- Developing trial documents—PILs and consent forms	- Two patient champions and other patients
						- TSC	- Miscarriage Association representative
Curley et al*.* [67]	Multinational	- NHS Blood and Transplant (NHSBT); others	Neonatal	Severe thrombocytopenia	Supplementary material (protocol)	- TSC	- Parent representative(s)
Manley et al. [68]	Australia	- Australian National Health and Medical Research Council (NHMRC) - Monash University	Neonatal	Neonatal respiratory conditions	Methods; supplementary protocol and published protocol [69]	- Trial co-investigator - Provided input into trial design	- Life’s Little Treasures Foundation representative
Coomarasamy et al*.* [70]	UK	NIHR	Maternal	Miscarriage prevention	Authorship, Supplementary material (protocol)	- TSC - Two co-authors of trial report	- Tommy’s representative - Miscarriage Association representative and Tommy's Charity representative
Dorling et al*.* [71]	UK	NIHR	Neonatal	Feeding	Authorship; supplementary material (protocol). PPI also reported in published protocol [72 ]	- TSC	- Representatives from PPI groups
						- Co-authors of trial report and protocol	- BLISS representatives
						- Consulted on trial design/acceptability	- TAMBA (Twins And Multiple Births Association) - BLISS - SSNAP
Deprest, Nicolaides et al*.* [73]	Multinational	- European Commission - KU Leuven - Wellcome Trust - Engineering and Physical Sciences Research Council (UK); others	Maternal	Congenital diaphragmatic hernia	Supplementary material (protocol)	- Data Monitoring and Safety Committee (DMSC)	- CDH representative
Deprest, Benachi et al*.* [74]	Multinational	- European Commission - KU Leuven - Wellcome Trust - Engineering and Physical Sciences Research Council (UK); others	Maternal	Congenital diaphragmatic hernia	Supplementary material (protocol)	- DMSC	- CDH representative
Hodgson et al*.* [75]	Australia	NHMRC	Neonatal	Neonatal respiratory distress	Supplementary material (protocol previously published in *BMJ Open* [76 ])	- Consulted on trial design/acceptability	- Parents of infants who participated in pilot trial
*BJOG*							
Smith et al. [77]	Ireland	Health Research Board (HRB) Ireland	Maternal	Caesarean section	Methods	- TSC	- Maternal service user
Bellad et al*.* [78]	India	Thrasher Research Foundation (US)	Maternal	Preterm birth	Methods	- Protocol development	- Pregnant women, community leaders, potential participants
Hyldig et al*.* [79]	Denmark	- University of Southern Denmark - Odense University Hospital - Lundbeckfonden - Smith & Nephew	Maternal	Surgical/operative birth infection	Methods	- Feedback on information material, questionnaire and patient follow-up	- Women giving birth at the main trial site
Goldenberg et al*.* [80]	Multinational	- Bill & Melinda Gates Foundation - Eunice Kennedy Shriver National Institute of Child Health	Maternal	Maternal and neonatal mortality	Methods	- Sought input on trial design	- Community meetings: women, families and community leaders
Ahmed et al*.* [81]	UK	Medical Research Council	Maternal	Preeclampsia	Methods, collaborators' information, supplementary protocol	- TSC	- Action on Pre-eclampsia patient–parent representative
Bick et al*.* [82]	UK	NIHR	Maternal	Postnatal weight management	Methods. Protocol [83]	- Trial design - Trial conduct	- Four women with experience of pregnancy with a BMI of 25 or more
						- TSC	- Lay member
Beckmann et al*.* [84]	Australia	The Mater Foundation	Maternal	Induction of labour	Methods	- Trial design - Reviewed protocol	- Maternal Choices Australia
Ngai et al*.* [85]	Hong Kong, China	General Research Fund (Hong Kong government)	Maternal	Postpartum depression	Methods	- Trial design	- Expectant parents
Slade et al*.* [86]	UK	NIHR	Maternal	Traumatic childbirth—PTSD	Authorship, Methods. Protocol [87]	- Trial management group - Two co-investigators - Reported involvement across trial lifespan (from development through to interpreting and disseminating findings) - Two co-authors of trial paper	- Three PPI contributors named, including Birth Trauma Association representative (co-author on main trial manuscript), and an Expert by Experience
Husain et al. [88]	UK	Barts Charity	Maternal	Vaginal health	Methods	- Trial design and conduct - Protocol development	- Patient representatives
						- Future trial dissemination in a PPI session	- Patient network
Stegwee et al*.* [89]	Netherlands	ZonMw	Maternal	Caesarean section complications	Methods, funding information	- Trial design - review of protocol - Grant proposal for funding	- Dutch gynaecological patients’ association (patient involvement panel)
Gurol-Urganci et al*.* [90]	UK	The Health Foundation	Maternal	Birth injury prevention	Methods. Protocol [91]	- Independent Advisory Group	- Lay representatives
						- Development of intervention component (information sheet)	- PPI groups
Acosta-Manzano et al*.* [92]	Multinational	- European Union FP7 programme - ZonMw - Polish Ministry of Science - Odense University (Denmark) - CAIBER 1527-B-226 (Spain); others. Supported by NIHR Clinical Research Network	Maternal	Gestational diabetes mellitus	Methods	- Involved in development of trial’s interventions	- Women
Flenady et al*.*^93^ ]	Multinational	- NHMRC - Stillbirth Foundation Australia	Maternal	Stillbirth	Methods; acknowledgements. Protocol [94]	- Consulted on trial design/acceptability	- Aboriginal women and community representatives
						- Developed study material—brochure	- Perinatal Society of Australia and New Zealand (PSANZ) Consumer Advisory Group - Stillbirth Foundation Australia
van der Nelson et al. [30]	UK	- Ferring Pharmaceuticals - North Bristol NHS Trust	Maternal	Postpartum haemorrhage	Methods, strengths and limitations, acknowledgements	- Consulted on trial design - Reviewed PILs and questionnaire - Developed study protocol	- Maternal Service User Panel
*Neonatology*							
Schindler et al*.* [95]	Australia	Running for Premature Babies	Neonatal	Patent ductus arteriosus	Funding sources, Protocol [96]	- Study conception - Research question and trial proposal	- Running for Premature Babies founder
						- Trial design and acceptability	- The Royal Hospital for Women’s Quality and Patient Safety Committee
						- Plans to disseminate trial findings	- Running for Premature Babies - The Royal Hospital for Women Foundation
*Archives of Disease in Childhood—Fetal and Neonatal Edition*							
de Kort et al*.* [97]	Netherlands	- Fonds NutsOhra - ZonMw	Neonatal	Anaesthesia intubation	Acknowledgements	- Trial design - Trial evaluation	- Parents’ organisation (VOC)
Duley et al*.* [98]	UK	NIHR	Neonatal	Cord clamping	Methods, collaborators information section. Protocol [99]	- Co-investigators - Research question - Trial funding application - Trial design and conduct - Consulted on recruitment - Analysis and interpretation of results - Writing report of results	- Three parent representatives (two BLISS representatives and one NCT representative)
						- Protocol co-author	- NCT representative
						- TSC	- Two parents
*Pediatrics*							
Gaden et al*.* [100]	Multinational	Research Council of Norway	Neonatal	Mother-infant bonding, parental anxiety, and maternal depression	Methods, acknowledgements. Protocol [101]	- Protocol development - Research question - Acceptability of intervention	- Parents of premature infant for protocol feedback
						- Dissemination strategies - Consulted prior and during trial progress - Recruitment approach	- User Advisory Group (3 named)—parents of premature infants
*BMC Pregnancy and Childbirth*							
Clarke et al*.* [102]	Multinational	European Union FP7 programme	Maternal	Vaginal birth after caesarean	Authorship	- Trial report co-author	- AIMS UK representative

In 12 trials, only one PPI contributor was reported to be involved. In 11 trials, the number of PPI contributors reported ranged from two to five individuals. In the remaining 25 trials, it was unclear how many PPI contributors were involved, with the information provided indicating that multiple PPI contributors were involved at some stage of the trial.

A range of terms was used in the trial reports to describe PPI contributors. For this review, we categorised PPI contributors into seven main categories.

- ‘Consumer organisations/charities’: representatives from charities or other similar organisations focused on maternal and neonatal health.
- ‘Maternal service users and families’: PPI contributors who were described as parents, service users, or as pregnant women and women with personal experience of the condition or illness under investigation.
- ‘PPI contributors’: individuals who were described solely based on their role in relation to PPI, for example, being described as a lay member or as a member of the public.
- ‘Former trial/research participants or their guardians’: PPI contributors who had been a participant of a previous research study or trial. This category also included parents of neonates previously enrolled in a pilot trial.
- The three remaining categories were ‘patient representatives’, ‘community representatives/community leaders’, and ‘lay member of ethics committee/safety committee’.

Overall, PPI contributors associated with consumer or charity organisations constituted the largest group of contributors (*n* = 24 trials, 50%) followed by ‘*maternal service users and families*’ (*n* = 15 trials, 31%). A total of 21 consumer organisations and charities were reported as PPI contributors. Three UK organisations were the most commonly reported organisations: the National Childbirth Trust, BLISS, and the Miscarriage Association. Supplementary file 7 provides an overview of the organisations that were involved in PPI. Two trials did not provide the name of the organisations involved in PPI [36, 103].

**Fig. 2:**
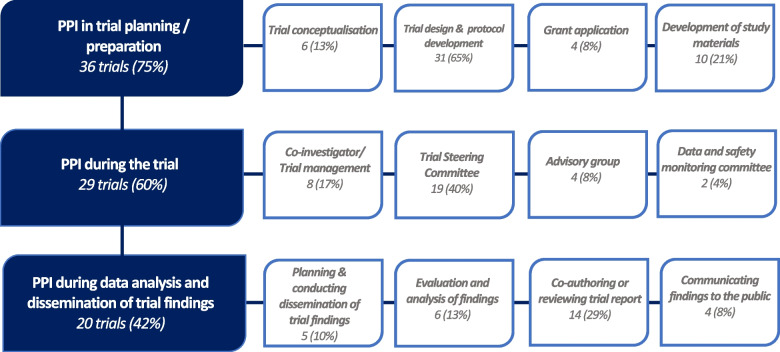
Type and stage of PPI reported in trials (*n* = 48)

PPI activities were reported at all stages of a trial, from trial conceptualisation to disseminating the findings, with 25 trials reporting that PPI contributors were involved at multiple stages of the trial (Fig. 2). For example, PPI contributors were involved in trial design and planning in 36 trials, including providing input into trial design or protocol development (65%), producing or reviewing study materials (21%), or completing grant applications (8%). Twenty-nine trials reported PPI during the running of the trial (60%), including contributor involvement on trial steering committees (*n* = 19 trials, 40%), and 42% of trials reported PPI during trial analysis and dissemination of findings, which included PPI contributors reviewing or analysing the findings (13%) or co-writing and reviewing trial reports (29%). Some trials reported PPI at multiple stages of a trial, because of the role of a PPI contributor within that trial. For example, a PPI contributor who was a trial co-investigator was involved from grant application, developing trial documents, attending trial meetings, to the co-writing of trial reports [39].

 None of the included trials provided information on financial reimbursements made to PPI contributors or appeared to report any training opportunities provided.

## Discussion

The results of this exploratory review demonstrate that, during the years 2017 to 2022, PPI reporting is lacking and, where reported, inconsistencies in reporting across trial reports exists. Previous reviews in other health research areas have found similarly low frequencies of PPI reporting [13, 23, 104, 105].

A review exploring PPI reporting in published orthopaedic trials (*n* = 475) found that only two trials reported PPI [104], whereas Gray et al*.* [105] found no evidence of Patient and Public Involvement and Engagement (PPIE) in 89 nursing trials reports. These frequencies of reporting are similar to those found in five of the six maternal and neonatal specific journals included in this review. Poor reporting limits our ability to understand how PPI is being conducted, which impacts the ability to reproduce methods of implementing PPI into research [11]. There is a reoccurring discussion on whether researchers can adequately assess or evaluate PPI’s impact on research [106, 107], with the suggestion that improved PPI reporting will allow for greater clarity on the impact of PPI within individual studies [13, 108]. Introducing reporting guidelines into maternal and neonatal specific journals could help to provide a clearer understanding of the extent that PPI is currently being implemented, in addition to raising the profile of PPI within the clinical research areas of maternal and neonatal health.

Only one trial report included in this review referred to the GRIPP2 checklist. The lack of consistency across the included journals on where PPI information was located within the trial report, in addition to the varying levels of details provided in terms of PPI aims and methods, would suggest that the GRIPP2 reporting guideline is not being consulted when reporting PPI. A review of PPI reporting in patient safety research found that 6% of 82 articles adhered to the GRIPP2 checklist [108]. The authors suggested that a published guide, providing detailed explanation on how GRIPP2 should be completed, could be beneficial in improving the use of the checklist and therefore enhance the consistency of reporting [108].

This review also found some discrepancies on what activity was considered ‘PPI’. For instance, in response to the journals’ PPI reporting prompt, two trials cited lay representation on an institutional Ethics Committee which, arguably, does not constitute researchers’ intentionally incorporating PPI into the trial. Another trial which did report some form of PPI additionally stated that a member of the trial team experienced the condition being investigated by the trial in response to the PPI reporting prompt. This would suggest that alongside the implementation of PPI reporting guidelines, it would be beneficial for journals requesting PPI reporting to provide information and examples of PPI in order to foster a common and shared understanding of constitutes ‘PPI’.

Price et al*.* [23] compared PPI reporting within *The BMJ* before and after the journal implemented PPI reporting guidelines and found that, while PPI reporting increased from 0.5% (2013–2014) to 11% (2015–2016), the overall quality and quantity of reporting remained low. Lang et al*.*’s [22] review of PPI reporting in articles published in *BMJ Open* in 2020 revealed that 21% reported PPI, with 44.5% of PPI reported occurring in the UK. Both Price et al*.* [23] and Lang et al. [22] noted that the lack of reporting cannot be entirely interpreted as simply an issue of under-reporting PPI that *was* conducted. Price et al*.* suggests that the overall lack of reporting in the *BMJ* during the selected years was due to a lack of PPI being conducted in general or researchers not wishing to share details of PPI that did not work well or that did not achieve its objectives [23]. While a substantial number of trials that reported PPI included this review were conducted in the UK, future work could be done to compare the implementation of PPI in research on a national and organisational level by country, as well as exploring trends of PPI reporting based on geographic location.

This review included two journals which have existing PPI reporting guidelines, both of which had higher levels of reporting in comparison to the majority of the seven other included journals. However, as previously highlighted, this may be due to authors intentionally submitting PPI informed research to journals with PPI guidelines [22]. Despite their levels of reporting, *BJOG* and the *BMJ* still had considerable numbers of trials stating that no PPI took place (40% and 43%, respectively). Most authors who reported that no PPI had occurred in response to the PPI guidelines did not provide reasons for this decision. As evidenced in two reviews on the impact of PPI reporting guidelines in journals [22, 23], reporting remained low in quality despite the implementation of a reporting guideline. The updated CONSORT 2025 checklist [109] and the SPIRIT 2025 (Standard Protocol Items: Recommendations for Interventional Trials) checklist [110] now request that authors provide information of PPI when reporting randomised trials and randomised trial protocols, respectively. As both checklists are requested of prospective authors by a substantial number of academic journals, including those included in this review, we would anticipate that this should increase the frequency of PPI reporting. However, trial journals can still implement PPI reporting guidelines to raise awareness of and frequency of PPI reporting across all research reports.

The numerous existing organisations who advocate for specific maternal and neonatal health and health needs, such as preeclampsia or miscarriage, enables trial research teams to have access to individuals with experiential knowledge to take part in PPI. This was evidenced by half of the included trials that reported PPI involving an individual linked to a maternal or neonatal charity or consumer organisation. However, several of the trials that reported PPI did not include information that would provide insight on how they were able to recruit PPI contributors. For example, in nine trials, PPI contributors were discussed only as ‘contributors’; no other information was provided to explain how and why they, as individuals, were involved in a particular trial. Involving individuals with lived experience who are most likely to benefit from the research is an integral aspect of why PPI has the potential to improve the relevance of research [107, 111, 112]. As such, it would be helpful to have more information provided on who was involved in PPI.

### Strengths and limitations

The review provides an overview of PPI reporting in maternal and neonatal trials published between 2017 and 2022, which to our knowledge has not been addressed previously, and which provides an overview of the current landscape from which to build on. The strengths of this study include the range of journals and years included, with the latter providing contemporary insight. The review, however, has some limitations. As only a sample of trials published in *BMC Pregnancy & Childbirth* were included in this review, it is possible that PPI was reported in other eligible trials reports published in this journal. However, based on the low levels of PPI reported in the included journals overall, particularly the neonatal and maternal health specific journals, it is unlikely that the frequency of reporting would have changed substantially. It is also possible that had we chosen different journals for inclusion in this review, our results may have been different.

There is also the potential that trial researchers did conduct PPI but did not explicitly report this in the trial report, particularly in journals without PPI guidelines. For instance, information provided in two included trials were only identifiable as related to PPI due to further information provided in their associated trial protocols. This suggests that other trials that conducted PPI, and acknowledged an individual’s involvement but did not explicitly cite their role as a PPI contributor, may have gone undetected in this review. Emailing authors to request PPI information was considered for this current review; however, a similar review that used this method reported a low response rate which had little impact on the review’s findings [13].

## Conclusion

The reporting of PPI in maternal and neonatal clinical trials is lacking, and there is a wide variation in the depths of information provided, including those involved as PPI contributors and how they contributed to the trial. Implementing PPI reporting guidelines in academic journals may be beneficial in prompting researchers to provide information on whether PPI was conducted. Implementing structured reporting frameworks, such as the GRIPP2 or the GRIPP2 short form, alongside journal reporting guidelines could help to standardise PPI reporting [11].

## Supplementary Information

Additional file 1. SCOPUS search strategy and results.

Additional file 2. Data extraction form.

Additional file 3. All included trials and their associated trial protocol (*n* = 352).

Additional file 4. Characteristics of included trials (*n* = 352).

Additional file 5. PPI reporting in trial reports by journal and year of publication.

Additional file 6. Conditions investigated in trials that reported PPI (*n* = 48).

Additional file 7. Charities/consumer organisations involved in PPI, as reported in 24 trial reports.

Additional file 8. The PRISMA 2020 reporting checklist.

## Data Availability

The dataset created and analysed for the purpose of this review is available from the authors, upon reasonable request.
