# Supplementary material for: Patient and public involvement (PPI) reporting in maternal and neonatal clinical trials: an exploratory review
Source: Trials. 2026 Mar 6;27:300. doi: 10.1186/s13063-026-09580-z (PMC13081287; doi:10.1186/s13063-026-09580-z)
Supplement: Supplementary file 1 — Additional file 1. SCOPUS search strategy and results. [file 13063_2026_9580_MOESM1_ESM.docx]

Additional file 1: SCOPUS search strategy and results

| Scopus Search 7^th^ July 2023 | | |
| --- | --- | --- |
| **Journal** | **Search** | **Total retrieved** |
| *BMJ* | SOURCE-ID ( 51748 ) AND PUBYEAR > 2016 AND PUBYEAR < 2023 AND ( LIMIT-TO ( DOCTYPE , "ar" ) OR LIMIT-TO ( DOCTYPE , "re" ) OR LIMIT-TO ( DOCTYPE , "cp" ) ) AND ( LIMIT-TO ( EXACTSRCTITLE , "BMJ" ) OR LIMIT-TO ( EXACTSRCTITLE , "BMJ Online" ) ) | 2,806 |
| *Lancet* | SOURCE-ID ( 16590 ) AND PUBYEAR > 2016 AND PUBYEAR < 2023 AND ( LIMIT-TO ( DOCTYPE , "ar" ) OR LIMIT-TO ( DOCTYPE , "cp" ) OR LIMIT-TO ( DOCTYPE , "re" ) ) AND ( LIMIT-TO ( SRCTYPE , "j" ) ) | 2,601 |
| *NEJM* | SOURCE-ID ( 15847 ) AND PUBYEAR > 2016 AND PUBYEAR < 2023 AND ( LIMIT-TO ( DOCTYPE , "ar" ) OR LIMIT-TO ( DOCTYPE , "re" ) OR LIMIT-TO ( DOCTYPE , "cp" ) ) AND ( LIMIT-TO ( EXACTSRCTITLE , "New England Journal Of Medicine" ) ) | 3,465 |
| *Obstetrics and Gynecology* | SOURCE-ID ( 28089 ) AND PUBYEAR > 2016 AND PUBYEAR < 2023 AND ( LIMIT-TO ( DOCTYPE , "ar" ) OR LIMIT-TO ( DOCTYPE , "cp" ) OR LIMIT-TO ( DOCTYPE , "re" ) ) | 2,276 |
| *BMC Pregnancy & Childbirth** | SOURCE-ID ( 12550 ) AND PUBYEAR > 2016 AND PUBYEAR < 2023 AND ( LIMIT-TO ( DOCTYPE , "ar" ) OR LIMIT-TO ( DOCTYPE , "re" ) ) AND ( LIMIT-TO ( EXACTSRCTITLE , "BMC Pregnancy And Childbirth" ) ) | 4,034 |
| *BJOG** | SOURCE-ID ( 12549 ) AND PUBYEAR > 2016 AND PUBYEAR < 2023 AND ( LIMIT-TO ( DOCTYPE , "ar" ) OR LIMIT-TO ( DOCTYPE , "re" )) AND ( LIMIT-TO ( EXACTSRCTITLE , "BJOG An International Journal Of Obstetrics And Gynaecology" ) ) | 1,219 |
| *Neonatology* | SOURCE-ID ( 5200152603 ) AND PUBYEAR > 2016 AND PUBYEAR < 2023 AND ( LIMIT-TO ( DOCTYPE , "ar" ) OR LIMIT-TO ( DOCTYPE , "re" ) OR LIMIT-TO ( DOCTYPE , "cp" ) ) AND ( LIMIT-TO ( EXACTSRCTITLE , "Neonatology" ) ) | 596 |
| *Pediatrics* | SOURCE-ID ( 15756 ) AND PUBYEAR > 2016 AND PUBYEAR < 2023 AND ( LIMIT-TO ( DOCTYPE , "ar" ) OR LIMIT-TO ( DOCTYPE , "cp" ) OR LIMIT-TO ( DOCTYPE , "re" ) ) AND ( LIMIT-TO ( EXACTSRCTITLE , "Pediatrics" ) ) AND ( LIMIT-TO ( SRCTYPE , "j" ) ) | 3,695 |
| *Archives Of Disease In Childhood -Fetal And Neonatal Edition* | SOURCE-ID ( 30081 ) AND PUBYEAR > 2016 AND PUBYEAR < 2023 AND ( LIMIT-TO ( DOCTYPE , "ar" ) OR LIMIT-TO ( DOCTYPE , "re" ) OR LIMIT-TO ( DOCTYPE , "cp" ) ) AND ( LIMIT-TO ( EXACTSRCTITLE , "Archives Of Disease In Childhood Fetal And Neonatal Edition" ) ) | 619 |
| **Total** |  | **21,311 records** |
