# Supplementary material for: Patient and public involvement (PPI) reporting in maternal and neonatal clinical trials: an exploratory review
Source: Trials. 2026 Mar 6;27:300. doi: 10.1186/s13063-026-09580-z (PMC13081287; doi:10.1186/s13063-026-09580-z)
Supplement: Supplementary file 2 — Additional file 2. Data extraction form. [file 13063_2026_9580_MOESM2_ESM.docx]

Additional file 2: Data extraction form

| **General data of included trials** |  |
| --- | --- |
| Reference |  |
| Author |  |
| Year published |  |
| Journal |  |
| Country |  |
| Trial registration number |  |
| Research topic | - Maternal trials - Neonatal trials |
| Time of intervention |  |
| Health condition/Area under evaluation |  |
| Details of the intervention being tested |  |
| Study type (purpose of the trial) | - Prevention - Treatment - Diagnostic/Screening - Health Services Research - Other |
| Intervention types | - Drug - Device - Behavioural - Procedure - Dietary supplement - Diagnostic test - Vaccine - Other - Testing two or more interventions |
| Funding body |  |
| **Does the publishing journal mandate the inclusion of PPI information?** | Yes/No |
| **Any details of PPI activity provided in the published trial article or trial protocol?** | Yes/No   - In published article - In trial protocol |
| **Where is the PPI reported?** | - Author affiliation information - Methods - Results - Discussion - Acknowledgements - Funding information - Supplementary material - In the protocol only |
| **Type of PPI contributor(s) involved** | - Trial participant(s) - Patient/Service user representative organisation - Members of the public - Other |
|  |  |
| **Number of PPI contributors involved** |  |
| **Trial stage that PPI occurs at** | **Planning of trial activities**   - Trial conceptualisation - Development of research question - Grant application - Protocol development - Developing study materials (e.g., patient information leaflet)   **Trial conduct**   - *Trial steering committee* - *Data monitoring committee* - *Trial management group* - *Community advisory groups*   **Interpretation and dissemination of trial results**   - Analysis and interpretation of results - Co-writing trial reports and manuscripts - Presentation of findings |
| **PPI recruitment methods described?** | Yes/No |
| **Funding provided for PPI contributors’ involvement?** | Yes/No  If yes, details of funding: … |
| **Training provided to PPI contributors?** | Yes/No  If yes, details of training: … |
