# Supplementary material for: Patient and public involvement (PPI) reporting in maternal and neonatal clinical trials: an exploratory review
Source: Trials. 2026 Mar 6;27:300. doi: 10.1186/s13063-026-09580-z (PMC13081287; doi:10.1186/s13063-026-09580-z)
Supplement: Supplementary file 3 — Additional file 3. All included trials and their associated trial protocol (n = 352). [file 13063_2026_9580_MOESM3_ESM.docx]

**Additional file 3: All included trials and their associated trial protocol (n=352)**

| **Reference of trial report** | **Reference for associated trial protocol** |
| --- | --- |
| Tappin D, Sinclair L, Kee F, McFadden M, Robinson-Smith L, Mitchell A, et al. Effect of financial voucher incentives provided with UK stop smoking services on the cessation of smoking in pregnant women (CPIT III): pragmatic, multicentre, single blinded, phase 3, randomised controlled trial. BMJ. 2022;379:e071522. <https://doi.org/10.1136/bmj-2022-071522>  **A trial protocol was also made available as a supplementary material to the main trial report.** | Sinclair L, McFadden M, Tilbrook H, Mitchell A, Keding A, Watson J, et al. The smoking cessation in pregnancy incentives trial (CPIT): study protocol for a phase III randomised controlled trial. Trials. 2020;21(1):183. <https://doi.org/10.1186/s13063-019-4042-8> |
| Taneja S, Chowdhury R, Dhabhai N, Upadhyay RP, Mazumder S, Sharma S, et al. Impact of a package of health, nutrition, psychosocial support, and WaSH interventions delivered during preconception, pregnancy, and early childhood periods on birth outcomes and on linear growth at 24 months of age: factorial, individually randomised controlled trial. Bmj. 2022;379:e072046. <https://doi.org/10.1136/bmj-2022-072046> | Taneja S, Chowdhury R, Dhabhai N, Mazumder S, Upadhyay RP, Sharma S, et al. Impact of an integrated nutrition, health, water sanitation and hygiene, psychosocial care and support intervention package delivered during the pre- and peri-conception period and/or during pregnancy and early childhood on linear growth of infants in the first two years of life, birth outcomes and nutritional status of mothers: study protocol of a factorial, individually randomized controlled trial in India. Trials. 2020;21(1):127. <https://doi.org/10.1186/s13063-020-4059-z> |
| Berlin I, Berlin N, Malecot M, Breton M, Jusot F, Goldzahl L. Financial incentives for smoking cessation in pregnancy: multicentre randomised controlled trial. BMJ. 2021;375:e065217. <https://doi.org/10.1136/bmj-2021-065217> | Berlin N, Goldzahl L, Jusot F, Berlin I. Protocol for study of financial incentives for smoking cessation in pregnancy (FISCP): randomised, multicentre study. BMJ Open. 2016;6(7):e011669. <https://doi.org/10.1136/bmjopen-2016-011669> |
| Cluver CA, Hiscock R, Decloedt EH, Hall DR, Schell S, Mol BW, et al. Use of metformin to prolong gestation in preterm pre-eclampsia: randomised, double blind, placebo controlled trial. BMJ. 2021;374:n2103. | Cluver C, Walker SP, Mol BW, Hall D, Hiscock R, Brownfoot FC, et al. A double blind, randomised, placebo-controlled trial to evaluate the efficacy of metformin to treat preterm pre-eclampsia (PI2 Trial): study protocol. BMJ Open. 2019;9(4):e025809. <https://doi.org/10.1136/bmjopen-2018-025809> |
| Hayes-Ryan D, Khashan AS, Hemming K, Easter C, Devane D, Murphy DJ, et al. Placental growth factor in assessment of women with suspected pre-eclampsia to reduce maternal morbidity: a stepped wedge cluster randomised control trial (PARROT Ireland). Bmj. 2021;374:n1857. <https://doi.org/10.1136/bmj.n1857> | Hayes-Ryan D, Hemming K, Breathnach F, Cotter A, Devane D, Hunter A, et al. PARROT Ireland: Placental growth factor in Assessment of women with suspected pre-eclampsia to reduce maternal morbidity: a Stepped Wedge Cluster Randomised Control Trial Research Study Protocol. BMJ Open. 2019;9(2):e023562. <https://doi.org/10.1136/bmjopen-2018-023562> |
| Boie S, Glavind J, Uldbjerg N, Steer PJ, Bor P. Continued versus discontinued oxytocin stimulation in the active phase of labour (CONDISOX): double blind randomised controlled trial. Bmj. 2021;373:n716. <https://doi.org/10.1136/bmj.n716> | Boie S, Glavind J, Uldbjerg N, Bakker JJH, van der Post JAM, Steer PJ, et al. CONDISOX- continued versus discontinued oxytocin stimulation of induced labour in a double-blind randomised controlled trial. BMC Pregnancy and Childbirth. 2019;19(1):320. <https://doi.org/10.1186/s12884-019-2461-x> |
| Gillespie BM, Webster J, Ellwood D, Thalib L, Whitty JA, Mahomed K, et al. Closed incision negative pressure wound therapy versus standard dressings in obese women undergoing caesarean section: multicentre parallel group randomised controlled trial. BMJ. 2021;373:n893. <https://doi.org/10.1136/bmj.n893> | Gillespie BM, Webster J, Ellwood D, Stapleton H, Whitty JA, Thalib L, et al. ADding negative pRESSure to improve healING (the DRESSING trial): a RCT protocol. BMJ Open. 2016;6(2):e010287. <https://doi.org/10.1136/bmjopen-2015-010287> |
| Wennerholm UB, Saltvedt S, Wessberg A, Alkmark M, Bergh C, Wendel SB, et al. Induction of labour at 41 weeks versus expectant management and induction of labour at 42 weeks (SWEdish Post-term Induction Study, SWEPIS): multicentre, open label, randomised, superiority trial. Bmj. 2019;367:l6131. <https://doi.org/10.1136/bmj.l6131> | Elden H, Hagberg H, Wessberg A, Sengpiel V, Herbst A, Bullarbo M, et al. Study protocol of SWEPIS a Swedish multicentre register based randomised controlled trial to compare induction of labour at 41 completed gestational weeks versus expectant management and induction at 42 completed gestational weeks. BMC Pregnancy Childbirth. 2016;16:49. <https://doi.org/10.1186/s12884-016-0836-9> |
| Henrichs J, Verfaille V, Jellema P, Viester L, Pajkrt E, Wilschut J, et al. Effectiveness of routine third trimester ultrasonography to reduce adverse perinatal outcomes in low risk pregnancy (the IRIS study): nationwide, pragmatic, multicentre, stepped wedge cluster randomised trial. Bmj. 2019;367:l5517. <https://doi.org/10.1136/bmj.l5517> | Henrichs J, Verfaille V, Viester L, Westerneng M, Molewijk B, Franx A, et al. Effectiveness and cost-effectiveness of routine third trimester ultrasound screening for intrauterine growth restriction: study protocol of a nationwide stepped wedge cluster-randomized trial in The Netherlands (The IRIS Study). BMC Pregnancy and Childbirth. 2016;16(1):310. <https://doi.org/10.1186/s12884-016-1104-8> |
| Keulen JK, Bruinsma A, Kortekaas JC, van Dillen J, Bossuyt PM, Oudijk MA, et al. Induction of labour at 41 weeks versus expectant management until 42 weeks (INDEX): multicentre, randomised non-inferiority trial. Bmj. 2019;364:l344. <https://doi.org/10.1136/bmj.l344> | Kortekaas JC, Bruinsma A, Keulen JKJ, van Dillen J, Oudijk MA, Zwart JJ, et al. Effects of induction of labour versus expectant management in women with impending post-term pregnancies: the 41 week – 42 week dilemma. BMC Pregnancy and Childbirth. 2014;14(1):350. <https://doi.org/10.1186/1471-2393-14-350> |
| Wen SW, White RR, Rybak N, Gaudet LM, Robson S, Hague W, et al. Effect of high dose folic acid supplementation in pregnancy on pre-eclampsia (FACT): double blind, phase III, randomised controlled, international, multicentre trial. Bmj. 2018;362:k3478. <https://doi.org/10.1136/bmj.k3478> | Wen SW, Champagne J, Rennicks White R, Coyle D, Fraser W, Smith G, et al. Effect of folic acid supplementation in pregnancy on preeclampsia: the folic acid clinical trial study. J Pregnancy. 2013;2013:294312. <https://doi.org/10.1155/2013/294312> |
| Adnan N, Conlan-Trant R, McCormick C, Boland F, Murphy DJ. Intramuscular versus intravenous oxytocin to prevent postpartum haemorrhage at vaginal delivery: randomised controlled trial. Bmj. 2018;362:k3546. <https://doi.org/10.1136/bmj.k3546> | Adnan N, Boland F, Murphy DJ. Intramuscular oxytocin versus intravenous oxytocin to prevent postpartum haemorrhage at vaginal delivery (LabOR trial): study protocol for a randomised controlled trial. Trials. 2017;18(1):541. https://doi.org/10.1186/s13063-017-2269-9 |
| Epidural T, Group PTC. Upright versus lying down position in second stage of labour in nulliparous women with low dose epidural: BUMPES randomised controlled trial. BMJ. 2017;359:j4471. <https://doi.org/10.1136/bmj.j4471> | Brocklehurst P. (2012) Protocol; A study of position during the late stages of labour in women with an epidural. The BUMPES study. Available at: https://www.birmingham.ac.uk/Documents/college-mds/trials/bctu/BUMPES/protocolv5.pdf |
| Velzel J, Vlemmix F, Opmeer BC, Molkenboer JF, Verhoeven CJ, van Pampus MG, et al. Atosiban versus fenoterol as a uterine relaxant for external cephalic version: randomised controlled trial. Bmj. 2017;356:i6773. <https://doi.org/10.1136/bmj.i6773> | Not available |
| Brocklehurst P, Field D, Greene K, Juszczak E, Keith R, Kenyon S, et al. Computerised interpretation of fetal heart rate during labour (INFANT): a randomised controlled trial. The Lancet. 2017;389(10080):1719-29. <https://doi.org/10.1016/S0140-6736(17)30568-8>  **A trial protocol was also made available as a supplementary material to the main trial report.** | Brocklehurst P. A study of an intelligent system to support decision making in the management of labour using the cardiotocograph - the INFANT study protocol. BMC Pregnancy Childbirth. 2016;16:10. <https://doi.org/10.1186/s12884-015-0780-0> |
| WOMAN Trial Collaborators. Effect of early tranexamic acid administration on mortality, hysterectomy, and other morbidities in women with post-partum haemorrhage (WOMAN): an international, randomised, double-blind, placebo-controlled trial. Lancet. 2017;389(10084):2105-16. <https://doi.org/10.1016/s0140-6736(17)30638-4> | Shakur H, Elbourne D, Gülmezoglu M, Alfirevic Z, Ronsmans C, Allen E, et al. The WOMAN Trial (World Maternal Antifibrinolytic Trial): tranexamic acid for the treatment of postpartum haemorrhage: an international randomised, double blind placebo controlled trial. Trials. 2010;11:40. <https://doi.org/10.1186/1745-6215-11-40> |
| Mundle S, Bracken H, Khedikar V, Mulik J, Faragher B, Easterling T, et al. Foley catheterisation versus oral misoprostol for induction of labour in hypertensive women in India (INFORM): a multicentre, open-label, randomised controlled trial. Lancet. 2017;390(10095):669-80. <https://doi.org/10.1016/s0140-6736(17)31367-3> | Bracken H, Mundle S, Faragher B, Easterling T, Haycox A, Turner M, et al. Induction of labour in pre-eclamptic women: a randomised trial comparing the Foley balloon catheter with oral misoprostol. BMC Pregnancy Childbirth. 2014;14:308. <https://doi.org/10.1186/1471-2393-14-308> |
| Forster DA, Moorhead AM, Jacobs SE, Davis PG, Walker SP, McEgan KM, et al. Advising women with diabetes in pregnancy to express breastmilk in late pregnancy (Diabetes and Antenatal Milk Expressing [DAME]): a multicentre, unblinded, randomised controlled trial. Lancet. 2017;389(10085):2204-13. <https://doi.org/10.1016/s0140-6736(17)31373-9> | Forster DA, Jacobs S, Amir LH, Davis P, Walker SP, McEgan K, et al. Safety and efficacy of antenatal milk expressing for women with diabetes in pregnancy: protocol for a randomised controlled trial. BMJ Open. 2014;4(10):e006571. <https://doi.org/10.1136/bmjopen-2014-006571> |
| Stocker M, van Herk W, El Helou S, Dutta S, Fontana MS, Schuerman F, et al. Procalcitonin-guided decision making for duration of antibiotic therapy in neonates with suspected early-onset sepsis: a multicentre, randomised controlled trial (NeoPIns). Lancet. 2017;390(10097):871-81. <https://doi.org/10.1016/s0140-6736(17)31444-7>  **A trial protocol was also made available as a supplementary material to the main trial report.** | Stocker M, Hop WC, van Rossum AM. Neonatal Procalcitonin Intervention Study (NeoPInS): Effect of Procalcitonin-guided decision making on duration of antibiotic therapy in suspected neonatal early-onset sepsis: A multi-centre randomized superiority and non-inferiority Intervention Study. BMC Pediatr. 2010;10:89. <https://doi.org/10.1186/1471-2431-10-89> |
| Feig DS, Donovan LE, Corcoy R, Murphy KE, Amiel SA, Hunt KF, et al. Continuous glucose monitoring in pregnant women with type 1 diabetes (CONCEPTT): a multicentre international randomised controlled trial. Lancet. 2017;390(10110):2347-59. <https://doi.org/10.1016/s0140-6736(17)32400-5> | Feig DS, Asztalos E, Corcoy R, De Leiva A, Donovan L, Hod M, et al. CONCEPTT: Continuous Glucose Monitoring in Women with Type 1 Diabetes in Pregnancy Trial: A multi-center, multi-national, randomized controlled trial - Study protocol. BMC Pregnancy and Childbirth. 2016;16(1):167. <https://doi.org/10.1186/s12884-016-0961-5> |
| Norman JE, Heazell AEP, Rodriguez A, Weir CJ, Stock SJE, Calderwood CJ, et al. Awareness of fetal movements and care package to reduce fetal mortality (AFFIRM): a stepped wedge, cluster-randomised trial. The Lancet. 2018;392(10158):1629-38. <https://doi.org/10.1016/S0140-6736(18)31543-5> | Norman JE, French R, Whyte S, Weir C. Study Protocol. Can Promoting Awareness of Fetal movements and Focussing Interventions Reduce Fetal Mortality - a stepped wedge cluster randomised trial? Clinical Trials.gov; 2017/ Available at: <https://cdn.clinicaltrials.gov/large-docs/22/NCT01777022/Prot_001.pdf> |
| Wilson MJA, MacArthur C, Hewitt CA, Handley K, Gao F, Beeson L, et al. Intravenous remifentanil patient-controlled analgesia versus intramuscular pethidine for pain relief in labour (RESPITE): an open-label, multicentre, randomised controlled trial. The Lancet. 2018;392(10148):662-72. <https://doi.org/10.1016/S0140-6736(18)31613-1> | Wilson M, MacArthur C, Gao Smith F, Homer L, Handley K, Daniels J. The RESPITE trial: remifentanil intravenously administered patient-controlled analgesia (PCA) versus pethidine intramuscular injection for pain relief in labour: study protocol for a randomised controlled trial. Trials. 2016;17(1):591. <https://doi.org/10.1186/s13063-016-1708-3> |
| Subtil D, Brabant G, Tilloy E, Devos P, Canis F, Fruchart A, et al. Early clindamycin for bacterial vaginosis in pregnancy (PREMEVA): a multicentre, double-blind, randomised controlled trial. Lancet. 2018;392(10160):2171-9. <https://doi.org/10.1016/s0140-6736(18)31617-9> | Not available |
| Hartley C, Moultrie F, Hoskin A, Green G, Monk V, Bell JL, et al. Analgesic efficacy and safety of morphine in the Procedural Pain in Premature Infants (Poppi) study: randomised placebo-controlled trial. Lancet. 2018;392(10164):2595-605. <https://doi.org/10.1016/s0140-6736(18)31813-0> | Slater R, Hartley C, Moultrie F, Adams E, Juszczak E, Rogers R, et al. A blinded randomised placebo-controlled trial investigating the efficacy of morphine analgesia for procedural pain in infants: Trial protocol. Wellcome Open Res. 2016;1:7. <https://doi.org/10.12688/wellcomeopenres.10005.2> |
| Hofmeyr GJ, Betrán AP, Singata-Madliki M, Cormick G, Munjanja SP, Fawcus S, et al. Prepregnancy and early pregnancy calcium supplementation among women at high risk of pre-eclampsia: a multicentre, double-blind, randomised, placebo-controlled trial. Lancet. 2019;393(10169):330-9. <https://doi.org/10.1016/s0140-6736(18)31818-x> | Hofmeyr GJ, Novikova N, Singata M. Protocol 11PRT/4028: long term calcium supplementation in women at high risk of pre-eclampsia: a randomised, placebo-controlled trial (PACTR201105000267371) The Lancet. <http://www.thelancet.com/protocol-reviews/11PRT-4028> |
| Bernitz S, Dalbye R, Zhang J, Eggebø TM, Frøslie KF, Olsen IC, et al. The frequency of intrapartum caesarean section use with the WHO partograph versus Zhang's guideline in the Labour Progression Study (LaPS): a multicentre, cluster-randomised controlled trial. Lancet. 2019;393(10169):340-8. <https://doi.org/10.1016/s0140-6736(18)31991-3> | Bernitz S, Dalbye R, Øian P, Zhang J, Eggebø TM, Blix E. Study protocol: the Labor Progression Study, LAPS - does the use of a dynamic progression guideline in labor reduce the rate of intrapartum cesarean sections in nulliparous women? A multicenter, cluster randomized trial in Norway. BMC Pregnancy and Childbirth. 2017;17(1):370. <https://doi.org/10.1186/s12884-017-1553-8> |
| Griffiths J, Jenkins P, Vargova M, Bowler U, Juszczak E, King A, et al. Enteral lactoferrin supplementation for very preterm infants: a randomised placebo-controlled trial. The Lancet. 2019;393(10170):423-33<https://doi.org/10.1016/S0140-6736(18)32221-9> | The ELFIN Trial Investigators Group. Summary Protocol for a Multi-Centre Randomised Controlled Trial of Enteral Lactoferrin Supplementation in Newborn Very Preterm Infants (ELFIN). Neonatology. 2018;114(2):142-8. <https://doi.org/10.1159/000488927> |
| Kajubi R, Ochieng T, Kakuru A, Jagannathan P, Nakalembe M, Ruel T, et al. Monthly sulfadoxine-pyrimethamine versus dihydroartemisinin-piperaquine for intermittent preventive treatment of malaria in pregnancy: a double-blind, randomised, controlled, superiority trial. Lancet. 2019;393(10179):1428-39. <https://doi.org/10.1016/s0140-6736(18)32224-4> | Dorsey G, Kamya M. (2018) Prevention of Malaria in HIV-uninfected Pregnant Women and Infants. Short Title: PROMOTE Birth Cohort 3 NCT02793622. Protocol Version 5.0 February 2018 Retrieved from: https://cdn.clinicaltrials.gov/large-docs/22/NCT02793622/Prot_SAP_000.pdf |
| Duhig KE, Myers J, Seed PT, Sparkes J, Lowe J, Hunter RM, et al. Placental growth factor testing to assess women with suspected pre-eclampsia: a multicentre, pragmatic, stepped-wedge cluster-randomised controlled trial. The Lancet. 2019;393(10183):1807-18. <https://doi.org/10.1016/S0140-6736(18)33212-4> | Not available |
| Knight M, Chiocchia V, Partlett C, Rivero-Arias O, Hua X, Hinshaw K, et al. Prophylactic antibiotics in the prevention of infection after operative vaginal delivery (ANODE): a multicentre randomised controlled trial. Lancet. 2019;393(10189):2395-403. <https://doi.org/10.1016/s0140-6736(19)30773-1>  **A trial protocol was also made available as a supplementary material to the main trial report.** | Knight M, Mottram L, Gray S, Partlett C, Juszczak E. Prophylactic antibiotics for the prevention of infection following operative vaginal delivery (ANODE): study protocol for a randomised controlled trial. Trials. 2018;19(1):395. <https://doi.org/10.1186/s13063-018-2787-0> |
| Chappell LC, Bell JL, Smith A, Linsell L, Juszczak E, Dixon PH, et al. Ursodeoxycholic acid versus placebo in women with intrahepatic cholestasis of pregnancy (PITCHES): a randomised controlled trial. Lancet. 2019;394(10201):849-60. <https://doi.org/10.1016/s0140-6736(19)31270-x> | Chappell LC, Chambers J, Dixon PH, Dorling J, Hunter R, Bell JL, et al. Ursodeoxycholic acid versus placebo in the treatment of women with intrahepatic cholestasis of pregnancy (ICP) to improve perinatal outcomes: protocol for a randomised controlled trial (PITCHES). Trials. 2018;19(1):657. <https://doi.org/10.1186/s13063-018-3018-4> |
| Easterling T, Mundle S, Bracken H, Parvekar S, Mool S, Magee LA, et al. Oral antihypertensive regimens (nifedipine retard, labetalol, and methyldopa) for management of severe hypertension in pregnancy: an open-label, randomised controlled trial. Lancet. 2019;394(10203):1011-21. <https://doi.org/10.1016/s0140-6736(19)31282-6> | Not available |
| Stahl A, Lepore D, Fielder A, Fleck B, Reynolds JD, Chiang MF, et al. Ranibizumab versus laser therapy for the treatment of very low birthweight infants with retinopathy of prematurity (RAINBOW): an open-label randomised controlled trial. Lancet. 2019;394(10208):1551-9. <https://doi.org/10.1016/s0140-6736(19)31344-3> | Supplement to: Stahl A, Lepore D, Fielder A, et al. Ranibizumab versus laser therapy for the treatment of very low birthweight infants with retinopathy of prematurity (RAINBOW): an open-label randomised controlled trial. Lancet 2019; published online  Sept 12. <https://doi.org/10.1016/s0140-6736(19)31344-3> |
| Chappell LC, Brocklehurst P, Green ME, Hunter R, Hardy P, Juszczak E, et al. Planned early delivery or expectant management for late preterm pre-eclampsia (PHOENIX): a randomised controlled trial. The Lancet. 2019;394(10204):1181-90. <https://doi.org/10.1016/S0140-6736(19)31963-4> | Chappell LC, Green M, Marlow N, Sandall J, Hunter R, Robson S, et al. Planned delivery or expectant management for late preterm pre-eclampsia: study protocol for a randomised controlled trial (PHOENIX trial). Trials. 2019;20(1):85. <https://doi.org/10.1186/s13063-018-3150-1> |
| Mazumder S, Taneja S, Dube B, Bhatia K, Ghosh R, Shekhar M, et al. Effect of community-initiated kangaroo mother care on survival of infants with low birthweight: a randomised controlled trial. Lancet. 2019;394(10210):1724-36. <https://doi.org/10.1016/s0140-6736(19)32223-8> | Mazumder S, Taneja S, Dalpath SK, Gupta R, Dube B, Sinha B, et al. Impact of community-initiated Kangaroo Mother Care on survival of low birth weight infants: study protocol for a randomized controlled trial. Trials. 2017;18(1):262. <https://doi.org/10.1186/s13063-017-1991-7> |
| Hoffman MK, Goudar SS, Kodkany BS, Metgud M, Somannavar M, Okitawutshu J, et al. Low-dose aspirin for the prevention of preterm delivery in nulliparous women with a singleton pregnancy (ASPIRIN): a randomised, double-blind, placebo-controlled trial. Lancet. 2020;395(10220):285-93. <https://doi.org/10.1016/s0140-6736(19)32973-3> | Hoffman MK, Goudar SS, Kodkany BS, Goco N, Koso-Thomas M, Miodovnik M, et al. A description of the methods of the aspirin supplementation for pregnancy indicated risk reduction in nulliparas (ASPIRIN) study. BMC Pregnancy Childbirth. 2017;17(1):135. <https://doi.org/10.1186/s12884-017-1312-x> |
| Chu JJ, Devall AJ, Beeson LE, Hardy P, Cheed V, Sun Y, et al. Mifepristone and misoprostol versus misoprostol alone for the management of missed miscarriage (MifeMiso): a randomised, double-blind, placebo-controlled trial. Lancet. 2020;396(10253):770-8. <https://doi.org/10.1016/s0140-6736(20)31788-8> | MifeMiso. A randomised placebo-controlled trial of mifepristone and misoprostol versus misoprostol alone in the medical management of missed miscarriage 2019 [Protocol 5.0 (27-June-2019). Retrieved from: <https://www.birmingham.ac.uk/Documents/college-mds/trials/bctu/MifeMiso/MifeMiso-Protocol-v5.0-clean-checked-for-accessibility.pdf> |
| Shahar-Nissan K, Pardo J, Peled O, Krause I, Bilavsky E, Wiznitzer A, et al. Valaciclovir to prevent vertical transmission of cytomegalovirus after maternal primary infection during pregnancy: a randomised, double-blind, placebo-controlled trial. The Lancet. 2020;396(10253):779-85. <https://doi.org/10.1016/S0140-6736(20)31868-7> | Not available |
| Lockman S, Brummel SS, Ziemba L, Stranix-Chibanda L, McCarthy K, Coletti A, et al. Efficacy and safety of dolutegravir with emtricitabine and tenofovir alafenamide fumarate or tenofovir disoproxil fumarate, and efavirenz, emtricitabine, and tenofovir disoproxil fumarate HIV antiretroviral therapy regimens started in pregnancy (IMPAACT 2010/VESTED): a multicentre, open-label, randomised, controlled, phase 3 trial. Lancet. 2021;397(10281):1276-92. <https://doi.org/10.1016/s0140-6736(21)00314-7> | Lockman S, Chinula L. (2017) IMPAACT 2010. Phase III Study of the Virologic Efficacy and Safety of Dolutegravir-Containing versus Efavirenz-Containing Antiretroviral Therapy  Regimens in HIV-1-Infected Pregnant Women and their Infants. “VESTED”: Virologic Efficacy and Safety of ART Combinations with TAF/TDF, EFV, and DTG. A Multisite Study of the International Maternal Pediatric Adolescent AIDS Clinical Trials Network. Protocol Version 2, 2017. <https://cdn.clinicaltrials.gov/large-docs/22/NCT03048422/Prot_ICF_000.pdf> |
| Caeymaex L, Astruc D, Biran V, Marcus L, Flamein F, Le Bouedec S, et al. An educational programme in neonatal intensive care units (SEPREVEN): a stepped-wedge, cluster-randomised controlled trial. Lancet. 2022;399(10322):384-92. <https://doi.org/10.1016/s0140-6736(21)01899-7> | Caeymaex L, Lebeaux C, Roze JC, Danan C, Reynaud A, Jung C, et al. Study on preventing adverse events in neonates (SEPREVEN): A stepped-wedge randomised controlled trial to reduce adverse event rates in the NICU. Medicine (Baltimore). 2020;99(31):e20912. <https://doi.org/10.1097/md.0000000000020912> |
| Edqvist M, Dahlen HG, Häggsgård C, Tern H, Ängeby K, Teleman P, et al. The effect of two midwives during the second stage of labour to reduce severe perineal trauma (Oneplus): a multicentre, randomised controlled trial in Sweden. The Lancet. 2022;399(10331):1242-53. <https://doi.org/10.1016/S0140-6736(22)00188-X> | Edqvist M, Dahlen HG, Häggsgård C, Tern H, Ängeby K, Tegerstedt G, et al. One Plus One Equals Two—will that do? A trial protocol for a Swedish multicentre randomised controlled trial to evaluate a clinical practice to reduce severe perineal trauma. Trials. 2020;21(1):945. <https://doi.org/10.1186/s13063-020-04837-7> |
| Endler M, Petro G, Gemzell Danielsson K, Grossman D, Gomperts R, Weinryb M, et al. A telemedicine model for abortion in South Africa: a randomised, controlled, non-inferiority trial. Lancet. 2022;400(10353):670-9. <https://doi.org/10.1016/s0140-6736(22)01474-x> | Not available |
| Schmitz T, Doret-Dion M, Sentilhes L, Parant O, Claris O, Renesme L, et al. Neonatal outcomes for women at risk of preterm delivery given half dose versus full dose of antenatal betamethasone: a randomised, multicentre, double-blind, placebo-controlled, non-inferiority trial. Lancet. 2022;400(10352):592-604. <https://doi.org/10.1016/s0140-6736(22)01535-5>  **A trial protocol was also made available as a supplementary material to the main trial report.** | Schmitz T, Alberti C, Ursino M, Baud O, Aupiais C. Full versus half dose of antenatal betamethasone to prevent severe neonatal respiratory distress syndrome associated with preterm birth: study protocol for a randomised, multicenter, double blind, placebo-controlled, non-inferiority trial (BETADOSE). BMC Pregnancy Childbirth. 2019;19(1):67. <https://doi.org/10.1186/s12884-019-2206-x> |
| Hodgetts Morton V, Toozs-Hobson P, Moakes CA, Middleton L, Daniels J, Simpson NAB, et al. Monofilament suture versus braided suture thread to improve pregnancy outcomes after vaginal cervical cerclage (C-STICH): a pragmatic randomised, controlled, phase 3, superiority trial. Lancet. 2022;400(10361):1426-36. <https://doi.org/10.1016/s0140-6736(22)01808-6> | Israfil-Bayli F, Morton VH, Hewitt CA, Ewer AK, Gray J, Norman J, et al. C-STICH: Cerclage Suture Type for an Insufficient Cervix and its effect on Health outcomes-a multicentre randomised controlled trial. Trials. 2021;22(1):664. . <https://doi.org/10.1186/s13063-021-05629-3> |
| Bistervels IM, Buchmüller A, Wiegers HMG, F NÁ, Tardy B, Donnelly J, et al. Intermediate-dose versus low-dose low-molecular-weight heparin in pregnant and post-partum women with a history of venous thromboembolism (Highlow study): an open-label, multicentre, randomised, controlled trial. Lancet. 2022;400(10365):1777-87. <https://doi.org/10.1016/s0140-6736(22)02128-6> | Bleker SM, Buchmüller A, Chauleur C, F NÁ, Donnelly J, Verhamme P, et al. Low-molecular-weight heparin to prevent recurrent venous thromboembolism in pregnancy: Rationale and design of the Highlow study, a randomised trial of two doses. Thromb Res. 2016;144:62-8. <https://doi.org/10.1016/j.thromres.2016.06.001> |
| Chalmers JR, Haines RH, Bradshaw LE, Montgomery AA, Thomas KS, Brown SJ, et al. Daily emollient during infancy for prevention of eczema: the BEEP randomised controlled trial. Lancet. 2020;395(10228):962-72. <https://doi.org/10.1016/s0140-6736(19)32984-8>  **A trial protocol was also made available as a supplementary material to the main trial report.** | Chalmers JR, Haines RH, Mitchell EJ, Thomas KS, Brown SJ, Ridd M, et al. Effectiveness and cost-effectiveness of daily all-over-body application of emollient during the first year of life for preventing atopic eczema in high-risk children (The BEEP trial): protocol for a randomised controlled trial. Trials. 2017;18(1):343. <https://doi.org/10.1186/s13063-017-2031-3> |
| Casey BM, Thom EA, Peaceman AM, Varner MW, Sorokin Y, Hirtz DG, et al. Treatment of Subclinical Hypothyroidism or Hypothyroxinemia in Pregnancy. N Engl J Med. 2017;376(9):815-25 <https://doi.org/10.1056/NEJMoa1606205> | Biostatistical Coordinating Center for the NICHD MFMU Network. Protocol for: Casey BM, Thom EA, Peaceman AM, et al. Treatment of subclinical hypothyroidism or hypothyroxinemia in pregnancy. N Engl J Med 2017;376:815-25. DOI: 10.1056/NEJMoa1606205. 2006. <https://www.nejm.org/doi/suppl/10.1056/NEJMoa1606205/suppl_file/nejmoa1606205_protocol.pdf> |
| Kraft WK, Adeniyi-Jones SC, Chervoneva I, Greenspan JS, Abatemarco D, Kaltenbach K, et al. Buprenorphine for the Treatment of the Neonatal Abstinence Syndrome. N Engl J Med. 2017;376(24):2341-8. <https://doi.org/10.1056/NEJMoa1614835> | Protocol for: Kraft WK, Adeniyi-Jones SC, Chervoneva I, et al. Buprenorphine for the treatment of the neonatal  abstinence syndrome. N Engl J Med 2017;376:2341-8. DOI: 10.1056/NEJMoa1614835. Available at: <https://www.nejm.org/doi/suppl/10.1056/NEJMoa1614835/suppl_file/nejmoa1614835_protocol.pdf> |
| Rolnik DL, Wright D, Poon LC, O’Gorman N, Syngelaki A, Matallana CdP, et al. Aspirin versus Placebo in Pregnancies at High Risk for Preterm Preeclampsia. New England Journal of Medicine. 2017;377(7):613-22. <https://doi.org/doi:10.1056/NEJMoa1704559>  **A trial protocol was also made available as a supplementary material to the main trial report.** | O'Gorman N, Wright D, Rolnik DL, Nicolaides KH, Poon LC. Study protocol for the randomised controlled trial: combined multimarker screening and randomised patient treatment with ASpirin for evidence-based PREeclampsia prevention (ASPRE). BMJ Open. 2016;6(6):e011801. <https://doi.org/10.1136/bmjopen-2016-011801> |
| Tarnow-Mordi W, Morris J, Kirby A, Robledo K, Askie L, Brown R, et al. Delayed versus Immediate Cord Clamping in Preterm Infants. N Engl J Med. 2017;377(25):2445-55. <https://doi.org/10.1056/NEJMoa1711281> | Protocol for: Tarnow‑Mordi W, Morris J, Kirby A, et al. Delayed versus immediate cord clamping in preterm in‑  fants. N Engl J Med 2017;377:2445-55. DOI: 10.1056/NEJMoa1711281.  Available at: <https://www.nejm.org/doi/suppl/10.1056/NEJMoa1711281/suppl_file/nejmoa1711281_protocol.pdf> |
| Semrau KEA, Hirschhorn LR, Marx Delaney M, Singh VP, Saurastri R, Sharma N, et al. Outcomes of a Coaching-Based WHO Safe Childbirth Checklist Program in India. N Engl J Med. 2017;377(24):2313-24. <https://doi.org/10.1056/NEJMoa1701075>  **A trial protocol was also made available as a supplementary material to the main trial report.** | Semrau K, Hirschhorn L, Kodkany B, Spector J, Tuller D, King G, et al. Effectiveness of the WHO Safe Childbirth Checklist program in reducing severe maternal, fetal, and newborn harm in Uttar Pradesh, India: Study protocol for a matched-pair, cluster-randomized controlled trial. Trials. 2016;17. https://doi.org/10.1186/s13063-016-1673-x |
| Schreiber CA, Creinin MD, Atrio J, Sonalkar S, Ratcliffe SJ, Barnhart KT. Mifepristone Pretreatment for the Medical Management of Early Pregnancy Loss. N Engl J Med. 2018;378(23):2161-70. <https://doi.org/10.1056/NEJMoa1715726> | Schreiber CA, Creinin MD, Atrio J, Sonalkar S, Ratcliffe SJ, Barnhart KT. Mifepristone pretreatment  for the medical management of early pregnancy loss. N Engl J Med 2018;378:2161-70. DOI: 10.1056/NEJMoa1715726. Available at: <https://www.nejm.org/doi/suppl/10.1056/NEJMoa1715726/suppl_file/nejmoa1715726_protocol.pdf> |
| Jourdain G, Ngo-Giang-Huong N, Harrison L, Decker L, Khamduang W, Tierney C, et al. Tenofovir versus Placebo to Prevent Perinatal Transmission of Hepatitis B. N Engl J Med. 2018;378(10):911-23. <https://doi.org/10.1056/NEJMoa1708131> | 1. Jourdain G, Ngo-Giang-Huong N, Cressey TR, Hua L, Harrison L, Tierney C, et al. Prevention of mother-to-child transmission of hepatitis B virus: a phase III, placebo-controlled, double-blind, randomized clinical trial to assess the efficacy and safety of a short course of tenofovir disoproxil fumarate in women with hepatitis B virus e-antigen. BMC Infect Dis. 2016;16:393. <https://doi.org/10.1186/s12879-016-1734-5> |
| Grobman WA, Rice MM, Reddy UM, Tita ATN, Silver RM, Mallett G, et al. Labor Induction versus Expectant Management in Low-Risk Nulliparous Women. N Engl J Med. 2018;379(6):513-23. <https://doi.org/10.1056/NEJMoa1800566> | Protocol for: Grobman WA, Rice MM, Reddy UM, et al. Labor induction versus expectant management in lowrisk nulliparous women. N Engl J Med 2018;379:513-23. DOI: 10.1056/NEJMoa1800566. <https://www.nejm.org/doi/suppl/10.1056/NEJMoa1800566/suppl_file/nejmoa1800566_protocol.pdf> |
| Widmer M, Piaggio G, Nguyen TMH, Osoti A, Owa OO, Misra S, et al. Heat-Stable Carbetocin versus Oxytocin to Prevent Hemorrhage after Vaginal Birth. N Engl J Med. 2018;379(8):743-52. <https://doi.org/10.1056/NEJMoa1805489>  -52  **A trial protocol was also made available as a supplementary material to the main trial report.** | Widmer M, Piaggio G, Abdel-Aleem H, Carroli G, Chong YS, Coomarasamy A, et al. Room temperature stable carbetocin for the prevention of postpartum haemorrhage during the third stage of labour in women delivering vaginally: study protocol for a randomized controlled trial. Trials. 2016;17(1):143. <https://doi.org/10.1186/s13063-016-1271-y> |
| Sentilhes L, Winer N, Azria E, Sénat MV, Le Ray C, Vardon D, et al. Tranexamic Acid for the Prevention of Blood Loss after Vaginal Delivery. N Engl J Med. 2018;379(8):731-42. <https://doi.org/10.1056/NEJMoa1800942>  **A trial protocol was also made available as a supplementary material to the main trial report.** | Sentilhes L, Daniel V, Darsonval A, Deruelle P, Vardon D, Perrotin F, et al. Study protocol. TRAAP - TRAnexamic Acid for Preventing postpartum hemorrhage after vaginal delivery: a multicenter randomized, double-blind, placebo-controlled trial. BMC Pregnancy Childbirth. 2015;15:135. <https://doi.org/10.1186/s12884-015-0573-5> |
| Roth DE, Morris SK, Zlotkin S, Gernand AD, Ahmed T, Shanta SS, et al. Vitamin D Supplementation in Pregnancy and Lactation and Infant Growth. N Engl J Med. 2018;379(6):535-46. <https://doi.org/10.1056/NEJMoa1800927>  **A trial protocol was also made available as a supplementary material to the main trial report.** | Roth DE, Gernand AD, Morris SK, Pezzack B, Islam MM, Dimitris MC, et al. Maternal vitamin D supplementation during pregnancy and lactation to promote infant growth in Dhaka, Bangladesh (MDIG trial): study protocol for a randomized controlled trial. Trials. 2015;16:300. <https://doi.org/10.1186/s13063-015-0825-8> |
| Lissauer D, Wilson A, Hewitt CA, Middleton L, Bishop JRB, Daniels J, et al. A Randomized Trial of Prophylactic Antibiotics for Miscarriage Surgery. N Engl J Med. 2019;380(11):1012-21. <https://doi.org/10.1056/NEJMoa1808817>  **A trial protocol was also made available as a supplementary material to the main trial report.** | Lissauer D, Wilson A, Daniels J, Middleton L, Bishop J, Hewitt C, et al. Prophylactic antibiotics to reduce pelvic infection in women having miscarriage surgery - The AIMS (Antibiotics in Miscarriage Surgery) trial: study protocol for a randomized controlled trial. Trials. 2018;19(1):245. <https://doi.org/10.1186/s13063-018-2598-3> |
| Curley A, Stanworth SJ, Willoughby K, Fustolo-Gunnink SF, Venkatesh V, Hudson C, et al. Randomized Trial of Platelet-Transfusion Thresholds in Neonates. N Engl J Med. 2019;380(3):242-51. <https://doi.org/10.1056/NEJMoa1807320> | Protocol for: Curley A, Stanworth SJ, Willoughby K, et al. Randomized trial of platelet-transfusion thresholds in  neonates. N Engl J Med 2019;380:242-51. DOI: 10.1056/NEJMoa1807320. <https://www.nejm.org/doi/suppl/10.1056/NEJMoa1807320/suppl_file/nejmoa1807320_protocol.pdf> |
| Manley BJ, Arnolda GRB, Wright IMR, Owen LS, Foster JP, Huang L, et al. Nasal High-Flow Therapy for Newborn Infants in Special Care Nurseries. N Engl J Med. 2019;380(21):2031-40. <https://doi.org/10.1056/NEJMoa1812077>  **A trial protocol was also made available as a supplementary material to the main trial report.** | Manley BJ, Roberts CT, Arnolda GRB, Wright IMR, Owen LS, Dalziel KM, et al. A multicentre, randomised controlled, non-inferiority trial, comparing nasal high flow with nasal continuous positive airway pressure as primary support for newborn infants with early respiratory distress born in Australian non-tertiary special care nurseries (the HUNTER trial): study protocol. BMJ Open. 2017;7(6):e016746. <https://doi.org/10.1136/bmjopen-2017-016746> |
| Coomarasamy A, Devall AJ, Cheed V, Harb H, Middleton LJ, Gallos ID, et al. A Randomized Trial of Progesterone in Women with Bleeding in Early Pregnancy. N Engl J Med. 2019;380(19):1815-24. <https://doi.org/10.1056/NEJMoa1813730> | PRISM & Progesterone In Spontaneous Miscarriage (2019) Protocol for: Coomarasamy A, Devall AJ, Cheed V, et al. A randomized trial of progesterone in women with bleeding in early pregnancy. N Engl J Med 2019;380:1815-24. DOI: 10.1056/NEJMoa1813730. Available at: <https://www.nejm.org/doi/suppl/10.1056/NEJMoa1813730/suppl_file/nejmoa1813730_protocol.pdf> |
| Dorling J, Abbott J, Berrington J, Bosiak B, Bowler U, Boyle E, et al. Controlled Trial of Two Incremental Milk-Feeding Rates in Preterm Infants. N Engl J Med. 2019;381(15):1434-43. <https://doi.org/10.1056/NEJMoa1816654>  **A trial protocol was also made available as a supplementary material to the main trial report.** | Abbott J, Berrington J, Bowler U, Boyle E, Dorling J, Embleton N, et al. The Speed of Increasing milk Feeds: a randomised controlled trial. BMC Pediatr. 2017;17(1):39. <https://doi.org/10.1186/s12887-017-0794-z> |
| Makrides M, Best K, Yelland L, McPhee A, Zhou S, Quinlivan J, et al. A Randomized Trial of Prenatal n-3 Fatty Acid Supplementation and Preterm Delivery. N Engl J Med. 2019;381(11):1035-45. <https://doi.org/10.1056/NEJMoa1816832>  **A trial protocol was also made available as a supplementary material to the main trial report.** | Zhou SJ, Best K, Gibson R, McPhee A, Yelland L, Quinlivan J, et al. Study protocol for a randomised controlled trial evaluating the effect of prenatal omega-3 LCPUFA supplementation to reduce the incidence of preterm birth: the ORIP trial. BMJ Open. 2017;7(9):e018360. <https://doi.org/10.1136/bmjopen-2017-018360> |
| Gupta A, Montepiedra G, Aaron L, Theron G, McCarthy K, Bradford S, et al. Isoniazid Preventive Therapy in HIV-Infected Pregnant and Postpartum Women. N Engl J Med. 2019;381(14):1333-46. <https://doi.org/10.1056/NEJMoa1813060> | Protocol for: Gupta A, Montepiedra G, Aaron L, et al. Isoniazid preventive therapy in HIV-infected pregnant and postpartum women. N Engl J Med 2019;381:1333-46. DOI: 10.1056/NEJMoa1813060 Available at: https://www.nejm.org/doi/suppl/10.1056/NEJMoa1813060/suppl_file/nejmoa1813060_protocol.pdf |
| van Kempen A, Eskes PF, Nuytemans D, van der Lee JH, Dijksman LM, van Veenendaal NR, et al. Lower versus Traditional Treatment Threshold for Neonatal Hypoglycemia. N Engl J Med. 2020;382(6):534-44. <https://doi.org/10.1056/NEJMoa1905593> | Protocol for: van Kempen AAMW, Eskes PF, Nuytemans DHGM, et al. Lower versus traditional treatment threshold for neonatal hypoglycemia. N Engl J Med 2020;382:534-44. DOI: 10.1056/NEJMoa1905593. Available at: https://www.nejm.org/doi/suppl/10.1056/NEJMoa1905593/suppl_file/nejmoa1905593_protocol.pdf |
| Juul SE, Comstock BA, Wadhawan R, Mayock DE, Courtney SE, Robinson T, et al. A Randomized Trial of Erythropoietin for Neuroprotection in Preterm Infants. N Engl J Med. 2020;382(3):233-43. <https://doi.org/10.1056/NEJMoa1907423>  **A trial protocol was also made available as a supplementary material to the main trial report.** | Juul SE, Mayock DE, Comstock BA, Heagerty PJ. Neuroprotective potential of erythropoietin in neonates; design of a randomized trial. Matern Health Neonatol Perinatol. 2015;1:27. <https://doi.org/10.1186/s40748-015-0028-z> |
| Madhi SA, Polack FP, Piedra PA, Munoz FM, Trenholme AA, Simões EAF, et al. Respiratory Syncytial Virus Vaccination during Pregnancy and Effects in Infants. N Engl J Med. 2020;383(5):426-39. <https://doi.org/10.1056/NEJMoa1908380> | Protocol for: Madhi SA, Polack FP, Piedra PA, et al. Respiratory syncytial virus vaccination during pregnancy and effects in infants. N Engl J Med 2020;383:426-39. DOI: 10.1056/NEJMoa1908380. Available at: https://www.nejm.org/doi/suppl/10.1056/NEJMoa1908380/suppl_file/nejmoa1908380_protocol.pdf |
| Griffin MP, Yuan Y, Takas T, Domachowske JB, Madhi SA, Manzoni P, et al. Single-Dose Nirsevimab for Prevention of RSV in Preterm Infants. N Engl J Med. 2020;383(5):415-25. <https://doi.org/10.1056/NEJMoa1913556> | Protocol for: Griffin MP, Yuan Y, Takas T, et al. Single-dose nirsevimab for prevention of RSV in preterm infants. N Engl J Med 2020;383:415-25. DOI: 10.1056/NEJMoa1913556/ Available at: <https://www.nejm.org/doi/suppl/10.1056/NEJMoa1913556/suppl_file/nejmoa1913556_protocol.pdf> |
| Sparks TN, Lianoglou BR, Adami RR, Pluym ID, Holliman K, Duffy J, et al. Exome Sequencing for Prenatal Diagnosis in Nonimmune Hydrops Fetalis. New England Journal of Medicine. 2020;383(18):1746-56. <https://doi.org/doi:10.1056/NEJMoa2023643> | Not available |
| Kirpalani H, Bell EF, Hintz SR, Tan S, Schmidt B, Chaudhary AS, et al. Higher or Lower Hemoglobin Transfusion Thresholds for Preterm Infants. N Engl J Med. 2020;383(27):2639-51. <https://doi.org/10.1056/NEJMoa2020248> | Protocol for: Kirpalani H, Bell EF, Hintz SR, et al. Higher or lower hemoglobin transfusion thresholds for preterm infants. N Engl J Med 2020;383:2639-51. DOI: 10.1056/NEJMoa2020248. Available at: <https://www.nejm.org/doi/suppl/10.1056/NEJMoa2020248/suppl_file/nejmoa2020248_protocol.pdf> |
| The WHO ACTION Trials Collaborators. Antenatal Dexamethasone for Early Preterm Birth in Low-Resource Countries. New England Journal of Medicine. 2020;383(26):2514-25. <https://doi.org/doi:10.1056/NEJMoa2022398>  **A trial protocol was also made available as a supplementary material to the main trial report.** | WHO ACTION Trials Collaborators. The World Health Organization ACTION-I (Antenatal CorTicosteroids for Improving Outcomes in preterm Newborns) Trial: a multi-country, multi-centre, two-arm, parallel, double-blind, placebo-controlled, individually randomized trial of antenatal corticosteroids for women at risk of imminent birth in the early preterm period in hospitals in low-resource countries. Trials. 2019;20(1):507. <https://doi.org/10.1186/s13063-019-3488-z> |
| Pejovic NJ, Myrnerts Höök S, Byamugisha J, Alfvén T, Lubulwa C, Cavallin F, et al. A Randomized Trial of Laryngeal Mask Airway in Neonatal Resuscitation. N Engl J Med. 2020;383(22):2138-47. <https://doi.org/10.1056/NEJMoa2005333>  **A trial protocol was also made available as a supplementary material to the main trial report.** | Pejovic NJ, Myrnerts Höök S, Byamugisha J, Alfvén T, Lubulwa C, Cavallin F, et al. Neonatal resuscitation using a supraglottic airway device for improved mortality and morbidity outcomes in a low-income country: study protocol for a randomized trial. Trials. 2019;20(1):444. <https://doi.org/10.1186/s13063-019-3455-8> |
| Hillier TA, Pedula KL, Ogasawara KK, Vesco KK, Oshiro CES, Lubarsky SL, et al. A Pragmatic, Randomized Clinical Trial of Gestational Diabetes Screening. N Engl J Med. 2021;384(10):895-904. <https://doi.org/10.1056/NEJMoa2026028>  **A trial protocol was also made available as a supplementary material to the main trial report.** | Pedula KL, Hillier TA, Ogasawara KK, Vesco KK, Lubarsky S, Oshiro CES, et al. A randomized pragmatic clinical trial of gestational diabetes screening (ScreenR2GDM): Study design, baseline characteristics, and protocol adherence. Contemp Clin Trials. 2019;85:105829. <https://doi.org/10.1016/j.cct.2019.105829> |
| Sentilhes L, Sénat MV, Lous ML, Winer N, Rozenberg P, Kayem G, et al. Tranexamic Acid for the Prevention of Blood Loss after Cesarean Delivery. New England Journal of Medicine. 2021;384(17):1623-34. <https://doi.org/doi:10.1056/NEJMoa2028788>  **A trial protocol was also made available as a supplementary material to the main trial report.** | Sentilhes L, Daniel V, Deneux-Tharaux C. TRAAP2 - TRAnexamic Acid for Preventing postpartum hemorrhage after cesarean delivery: a multicenter randomized, doubleblind, placebo- controlled trial - a study protocol. BMC Pregnancy Childbirth. 2020;20(1):63. <https://doi.org/10.1186/s12884-019-2718-4> |
| Deprest JA, Nicolaides KH, Benachi A, Gratacos E, Ryan G, Persico N, et al. Randomized Trial of Fetal Surgery for Severe Left Diaphragmatic Hernia. N Engl J Med. 2021;385(2):107-18. <https://doi.org/10.1056/NEJMoa2027030> | Protocol for: Deprest JA, Nicolaides KH, Benachi A, et al. Randomized trial of fetal surgery for severe left diaphragmatic hernia. N Engl J Med 2021;385:107-18. DOI: 10.1056/NEJMoa2027030 . Available at: <https://www.nejm.org/doi/suppl/10.1056/NEJMoa2027030/suppl_file/nejmoa2027030_protocol.pdf> |
| Arya S, Naburi H, Kawaza K, Newton S, Anyabolu CH, Bergman N, et al. Immediate "Kangaroo Mother Care" and Survival of Infants with Low Birth Weight. N Engl J Med. 2021;384(21):2028-38. <https://doi.org/10.1056/NEJMoa2026486>  **A trial protocol was also made available as a supplementary material to the main trial report.** | WHO Immediate KMC Study Group. Impact of continuous Kangaroo Mother Care initiated immediately after birth (iKMC) on survival of newborns with birth weight between 1.0 to < 1.8 kg: study protocol for a randomized controlled trial. Trials. 2020;21(1):280. <https://doi.org/10.1186/s13063-020-4101-1> |
| Deprest JA, Benachi A, Gratacos E, Nicolaides KH, Berg C, Persico N, et al. Randomized Trial of Fetal Surgery for Moderate Left Diaphragmatic Hernia. N Engl J Med. 2021;385(2):119-29. <https://doi.org/10.1056/NEJMoa2026983> | Supplement to: Deprest JA, Benachi A, Gratacos E, et al. Randomized trial of fetal surgery for moderate left diaphragmatic hernia. N Engl J Med 2021;384:119-29. DOI: 10.1056/NEJMoa2026983 . Available at: <https://www.nejm.org/doi/suppl/10.1056/NEJMoa2026983/suppl_file/nejmoa2026983_protocol.pdf> |
| Hodgson KA, Owen LS, Kamlin COF, Roberts CT, Newman SE, Francis KL, et al. Nasal High-Flow Therapy during Neonatal Endotracheal Intubation. N Engl J Med. 2022;386(17):1627-37. <https://doi.org/10.1056/NEJMoa2116735>  **A trial protocol was also made available as a supplementary material to the main trial report.** | Hodgson KA, Owen LS, Kamlin CO, Roberts CT, Donath SM, Davis PG, et al. A multicentre, randomised trial of stabilisation with nasal high flow during neonatal endotracheal intubation (the SHINE trial): a study protocol. BMJ Open. 2020;10(10):e039230. <https://doi.org/10.1136/bmjopen-2020-039230> |
| Watterberg KL, Walsh MC, Li L, Chawla S, D'Angio CT, Goldberg RN, et al. Hydrocortisone to Improve Survival without Bronchopulmonary Dysplasia. N Engl J Med. 2022;386(12):1121-31. <https://doi.org/10.1056/NEJMoa2114897> | Protocol for: Watterberg KL, Walsh MC, Li L, et al. Hydrocortisone to improve survival without bronchopulmonary dysplasia. N Engl J Med 2022;386:1121-31. DOI: 10.1056/NEJMoa2114897 . Available at: <https://www.nejm.org/doi/suppl/10.1056/NEJMoa2114897/suppl_file/nejmoa2114897_protocol.pdf> |
| Tita AT, Szychowski JM, Boggess K, Dugoff L, Sibai B, Lawrence K, et al. Treatment for Mild Chronic Hypertension during Pregnancy. N Engl J Med. 2022;386(19):1781-92. <https://doi.org/10.1056/NEJMoa2201295> | Protocol for: Tita AT, Szychowski JM, Boggess K, et al. Treatment for mild chronic hypertension during pregnancy. N Engl J Med 2022;386:1781-92. DOI: 10.1056/NEJMoa2201295. Available at: <https://www.nejm.org/doi/suppl/10.1056/NEJMoa2201295/suppl_file/nejmoa2201295_protocol.pdf> |
| Wu YW, Comstock BA, Gonzalez FF, Mayock DE, Goodman AM, Maitre NL, et al. Trial of Erythropoietin for Hypoxic-Ischemic Encephalopathy in Newborns. N Engl J Med. 2022;387(2):148-59. <https://doi.org/10.1056/NEJMoa2119660>.  **A trial protocol was also made available as a supplementary material to the main trial report.** | Juul SE, Comstock BA, Heagerty PJ, Mayock DE, Goodman AM, Hauge S, et al. High-Dose Erythropoietin for Asphyxia and Encephalopathy (HEAL): A Randomized Controlled Trial - Background, Aims, and Study Protocol. Neonatology. 2018;113(4):331-8. <https://doi.org/10.1159/000486820> |
| Clasen TF, Chang HH, Thompson LM, Kirby MA, Balakrishnan K, Díaz-Artiga A, et al. Liquefied Petroleum Gas or Biomass for Cooking and Effects on Birth Weight. N Engl J Med. 2022;387(19):1735-46. <https://doi.org/10.1056/NEJMoa2206734>  **A trial protocol was also made available as a supplementary material to the main trial report.** | Clasen T, Checkley W, Peel JL, Balakrishnan K, McCracken JP, Rosa G, et al. Design and Rationale of the HAPIN Study: A Multicountry Randomized Controlled Trial to Assess the Effect of Liquefied Petroleum Gas Stove and Continuous Fuel Distribution. Environ Health Perspect. 2020;128(4):47008. <https://doi.org/10.1289/ehp6407> |
| Crowther CA, Samuel D, McCowan LME, Edlin R, Tran T, McKinlay CJ. Lower versus Higher Glycemic Criteria for Diagnosis of Gestational Diabetes. N Engl J Med. 2022;387(7):587-98. <https://doi.org/10.1056/NEJMoa2204091>  **A trial protocol was also made available as a supplementary material to the main trial report.** | Crowther C, McCowan L, Rowan J, Edlin R, McKinlay C. Lower versus higher diagnostic criteria for the detection of gestational diabetes for reducing maternal and perinatal morbidity: study protocol for the GEMS randomised trial. BMC Pregnancy and Childbirth. 2020;20:547. <https://doi.org/10.1186/s12884-020-03252-9> |
| Bloomfield FH, Jiang Y, Harding JE, Crowther CA, Cormack BE. Early Amino Acids in Extremely Preterm Infants and Neurodisability at 2 Years. N Engl J Med. 2022;387(18):1661-72. <https://doi.org/10.1056/NEJMoa2204886>  **A trial protocol was also made available as a supplementary material to the main trial report.** | Bloomfield FH, Crowther CA, Harding JE, Conlon CA, Jiang Y, Cormack BE. The ProVIDe study: the impact of protein intravenous nutrition on development in extremely low birthweight babies. BMC Pediatr. 2015;15:100. <https://doi.org/10.1186/s12887-015-0411-y> |
| Hughes BL, Clifton RG, Rouse DJ, Saade GR, Dinsmoor MJ, Reddy UM, et al. A Trial of Hyperimmune Globulin to Prevent Congenital Cytomegalovirus Infection. N Engl J Med. 2021;385(5):436-44. <https://doi.org/10.1056/NEJMoa1913569> | Protocol for: Hughes BL, Clifton RG, Rouse DJ, et al. A trial of hyperimmune globulin to prevent congenital  cytomegalovirus infection. N Engl J Med 2021;385:436-44. DOI: 10.1056/NEJMoa1913569. Available at: <https://www.nejm.org/doi/suppl/10.1056/NEJMoa1913569/suppl_file/nejmoa1913569_protocol.pdf> |
| Collins CT, Makrides M, McPhee AJ, Sullivan TR, Davis PG, Thio M, et al. Docosahexaenoic Acid and Bronchopulmonary Dysplasia in Preterm Infants. N Engl J Med. 2017;376(13):1245-55. <https://doi.org/10.1056/NEJMoa1611942>  **A trial protocol was also made available as a supplementary material to the main trial report.** | Collins CT, Gibson RA, Makrides M, McPhee AJ, Sullivan TR, Davis PG, et al. The N3RO trial: a randomised controlled trial of docosahexaenoic acid to reduce bronchopulmonary dysplasia in preterm infants < 29 weeks' gestation. BMC Pediatr. 2016;16:72. <https://doi.org/10.1186/s12887-016-0611-0> |
| Bines JE, At Thobari J, Satria CD, Handley A, Watts E, Cowley D, et al. Human Neonatal Rotavirus Vaccine (RV3-BB) to Target Rotavirus from Birth. N Engl J Med. 2018;378(8):719-30. <https://doi.org/10.1056/NEJMoa1706804> | Protocol for: Bines JE, At Thobari J, Satria CD, et al. Human neonatal rotavirus vaccine (RV3-BB) to target rotavirus from birth. N Engl J Med 2018;378:719-30. DOI: 10.1056/NEJMoa1706804 . Available at: <https://www.nejm.org/doi/suppl/10.1056/NEJMoa1706804/suppl_file/nejmoa1706804_protocol.pdf> |
| Sagedal LR, Øverby NC, Bere E, Torstveit MK, Lohne-Seiler H, Småstuen M, et al. Lifestyle intervention to limit gestational weight gain: the Norwegian Fit for Delivery randomised controlled trial. Bjog. 2017;124(1):97-109. <https://doi.org/10.1111/1471-0528.13862> | Sagedal LR, Øverby NC, Lohne-Seiler H, Bere E, Torstveit MK, Henriksen T, et al. Study protocol: fit for delivery - can a lifestyle intervention in pregnancy result in measurable health benefits for mothers and newborns? A randomized controlled trial. BMC Public Health. 2013;13(1):132. <https://doi.org/10.1186/1471-2458-13-132> |
| Logtenberg S, Oude Rengerink K, Verhoeven C, Freeman L, Van Den Akker E, Godfried M, et al. Labour pain with remifentanil patient-controlled analgesia versus epidural analgesia: a randomised equivalence trial. BJOG: An International Journal of Obstetrics & Gynaecology. 2017;124(4):652-60. <https://doi.org/10.1111/1471-0528.14181> | Not available |
| Rasouli M, Atashsokhan G, Keramat A, Khosravi A, Fooladi E, Mousavi S. The impact of motivational interviewing on participation in childbirth preparation classes and having a natural delivery: a randomised trial. BJOG: An International Journal of Obstetrics & Gynaecology. 2017;124(4):631-9. <https://doi.org/10.1111/1471-0528.14397> | Not available |
| Yefet E, Taha H, Salim R, Hasanein J, Carmeli Y, Schwartz N, et al. Fixed time interval compared with on-demand oral analgesia protocols for post-caesarean pain: a randomised controlled trial. BJOG: An International Journal of Obstetrics & Gynaecology. 2017;124(7):1063-70. <https://doi.org/10.1111/1471-0528.14546> | Not available |
| Qian G, Xu X, Chen L, Xia S, Wang A, Chuai Y, et al. The effect of maternal low flow oxygen administration during the second stage of labour on umbilical cord artery pH: a randomised controlled trial. BJOG: An International Journal of Obstetrics & Gynaecology. 2017;124(4):678-85. <https://doi.org/10.1111/1471-0528.14418> | Not available |
| van Baaren GJ, Broekhuijsen K, van Pampus MG, Ganzevoort W, Sikkema JM, Woiski MD, et al. An economic analysis of immediate delivery and expectant monitoring in women with hypertensive disorders of pregnancy, between 34 and 37 weeks of gestation (HYPITAT-II). Bjog. 2017;124(3):453-61. <https://doi.org/10.1111/1471-0528.13957> | Langenveld J, Broekhuijsen K, van Baaren GJ, van Pampus MG, van Kaam AH, Groen H, et al. Induction of labour versus expectant monitoring for gestational hypertension or mild pre-eclampsia between 34 and 37 weeks' gestation (HYPITAT-II): a multicentre, open-label randomised controlled trial. BMC Pregnancy Childbirth. 2011;11:50. <https://doi.org/10.1186/1471-2393-11-50> |
| Fransen A, Van De Ven J, Schuit E, Van Tetering A, Mol B, Oei S. Simulation-based team training for multi-professional obstetric care teams to improve patient outcome: a multicentre, cluster randomised controlled trial. BJOG: An International Journal of Obstetrics & Gynaecology. 2017;124(4):641-50. <https://doi.org/10.1111/1471-0528.14369> | van de Ven J, Houterman S, Steinweg RAJQ, Scherpbier AJJA, Wijers W, Mol BWJ, et al. Reducing errors in health care: cost-effectiveness of multidisciplinary team training in obstetric emergencies (TOSTI study); a randomised controlled trial. BMC Pregnancy and Childbirth. 2010;10(1):59. <https://doi.org/10.1186/1471-2393-10-59> |
| Lain S, Roberts C, Bond D, Smith J, Morris J. An economic evaluation of planned immediate versus delayed birth for preterm prelabour rupture of membranes: findings from the PPROMT randomised controlled trial. BJOG: An International Journal of Obstetrics & Gynaecology. 2017;124(4):623-30. <https://doi.org/10.1111/1471-0528.14302> | Morris JM, Roberts CL, Crowther CA, Buchanan SL, Henderson-Smart DJ, Salkeld G. Protocol for the immediate delivery versus expectant care of women with preterm prelabour rupture of the membranes close to term (PPROMT) Trial [ISRCTN44485060]. BMC Pregnancy Childbirth. 2006;6:9. <https://doi.org/10.1186/1471-2393-6-9> |
| Tan P, Alzergany M, Adlan AS, Noor Azmi M, Omar S. Immediate compared with on-demand maternal full feeding after planned caesarean delivery: a randomised trial. BJOG: An International Journal of Obstetrics & Gynaecology. 2017;124(1):123-31. <https://doi.org/10.1111/1471-0528.14211> | Not available |
| Korjamo R, Mentula M, Heikinheimo O. Immediate versus delayed initiation of the levonorgestrel-releasing intrauterine system following medical termination of pregnancy – 1 year continuation rates: a randomised controlled trial. BJOG: An International Journal of Obstetrics & Gynaecology. 2017;124(13):1957-64. <https://doi.org/https://doi.org/10.1111/1471-0528.14802> | Not available |
| Willcox J, Wilkinson S, Lappas M, Ball K, Crawford D, McCarthy E, et al. A mobile health intervention promoting healthy gestational weight gain for women entering pregnancy at a high body mass index: the txt4two pilot randomised controlled trial. BJOG: An International Journal of Obstetrics & Gynaecology. 2017;124(11):1718-28 <https://doi.org/10.1111/1471-0528.14552> | Willcox JC, Campbell KJ, McCarthy EA, Wilkinson SA, Lappas M, Ball K, et al. Testing the feasibility of a mobile technology intervention promoting healthy gestational weight gain in pregnant women (txt4two) - study protocol for a randomised controlled trial. Trials. 2015;16(1):209. <https://doi.org/10.1186/s13063-015-0730-1> |
| Shaw KA, Lerma K, Shaw JG, Scrivner KJ, Hugin M, Hopkins FW, et al. Preoperative effects of mifepristone for dilation and evacuation after 19 weeks of gestation: a randomised controlled trial. Bjog. 2017;124(13):1973-81. <https://doi.org/10.1111/1471-0528.14900> | Not available |
| Maaløe N, Housseine N, Meguid T, Nielsen BB, Jensen A, Khamis RS, et al. Effect of locally tailored labour management guidelines on intrahospital stillbirths and birth asphyxia at the referral hospital of Zanzibar: a quasi-experimental pre-post study (The PartoMa study). Bjog. 2018;125(2):235-45. <https://doi.org/10.1111/1471-0528.14933> | Not available |
| Korjamo R, Mentula M, Heikinheimo O. Expulsions and adverse events following immediate and later insertion of a levonorgestrel-releasing intrauterine system after medical termination of late first- and second-trimester pregnancy: a randomised controlled trial. BJOG: An International Journal of Obstetrics & Gynaecology. 2017;124(13):1965-72. <https://doi.org/https://doi.org/10.1111/1471-0528.14813> | Not available |
| Ten Eikelder M, Van Baaren GJ, Oude Rengerink K, Jozwiak M, De Leeuw J, Kleiverda G, et al. Comparing induction of labour with oral misoprostol or Foley catheter at term: cost-effectiveness analysis of a randomised controlled multi-centre non-inferiority trial. BJOG: An International Journal of Obstetrics & Gynaecology. 2018;125(3):375-83. <https://doi.org/10.1111/1471-0528.14706> | ten Eikelder MLG, Neervoort F, Rengerink KO, Jozwiak M, de Leeuw J-W, de Graaf I, et al. Induction of labour with a Foley catheter or oral misoprostol at term: the PROBAAT-II study, a multicentre randomised controlled trial. BMC Pregnancy and Childbirth. 2013;13(1):67<https://doi.org/10.1186/1471-2393-13-67> |
| Vigil-Degracia P, Ludmir J, Ng J, Reyes-Tejada O, Nova C, Beltré A, et al. Is there benefit to continue magnesium sulphate postpartum in women receiving magnesium sulphate before delivery? A randomised controlled study. BJOG: An International Journal of Obstetrics & Gynaecology. 2018;125(10):1304-11. <https://doi.org/10.1111/1471-0528.15320> | Not available |
| Madsen A, Dow M, Lohse C, Tessmer‐Tuck J. Absorbable subcuticular staples versus suture for caesarean section closure: a randomised clinical trial. BJOG: An International Journal of Obstetrics & Gynaecology. 2019;126(4):502-10. <https://doi.org/10.1111/1471-0528.15532> | Not available |
| Luton D, Mitanchez D, Winer N, Muller F, Gallot D, Perrotin F, et al. A randomised controlled trial of amnioexchange for fetal gastroschisis. BJOG: An International Journal of Obstetrics & Gynaecology. 2019;126(10):1233-41. <https://doi.org/10.1111/1471-0528.15804> | Not available |
| Smith V, Begley C, Newell J, Higgins S, Murphy D, White M, et al. Admission cardiotocography versus intermittent auscultation of the fetal heart in low‐risk pregnancy during evaluation for possible labour admission – a multicentre randomised trial: the ADCAR trial. BJOG: An International Journal of Obstetrics & Gynaecology. 2019;126(1):114-21. <https://doi.org/10.1111/1471-0528.15448> | Not available |
| Bellad M, Hoffman M, Mallapur A, Charantimath U, Katageri G, Ganachari M, et al. Clindamycin to reduce preterm birth in a low resource setting: a randomised placebo-controlled clinical trial. BJOG: An International Journal of Obstetrics & Gynaecology. 2018;125(12):1601-9. <https://doi.org/10.1111/1471-0528.15290> | Not available |
| Hyldig N, Vinter C, Kruse M, Mogensen O, Bille C, Sorensen J, et al. Prophylactic incisional negative pressure wound therapy reduces the risk of surgical site infection after caesarean section in obese women: a pragmatic randomised clinical trial. BJOG: An International Journal of Obstetrics & Gynaecology. 2019;126(5):628-35. <https://doi.org/10.1111/1471-0528.15413> | Not available |
| Goldenberg R, Nathan R, Swanson D, Saleem S, Mirza W, Esamai F, et al. Routine antenatal ultrasound in low- and middle-income countries: first look - a cluster randomised trial. BJOG: An International Journal of Obstetrics & Gynaecology. 2018;125(12):1591-9. <https://doi.org/10.1111/1471-0528.15287> | McClure EM, Nathan RO, Saleem S, Esamai F, Garces A, Chomba E, et al. First look: a cluster-randomized trial of ultrasound to improve pregnancy outcomes in low income country settings. BMC Pregnancy and Childbirth. 2014;14(1):73. <https://doi.org/10.1186/1471-2393-14-73> |
| Unger J, Ronen K, Perrier T, Derenzi B, Slyker J, Drake A, et al. Short message service communication improves exclusive breastfeeding and early postpartum contraception in a low‐ to middle‐income country setting: a randomised trial. BJOG: An International Journal of Obstetrics & Gynaecology. 2018;125(12):1620-9. <https://doi.org/10.1111/1471-0528.15337> | Not available |
| Romijn A, Ravelli A, Bruijne M, Twisk J, Wagner C, Groot C, et al. Effect of a cluster randomised team training intervention on adverse perinatal and maternal outcomes: a stepped wedge study. BJOG: An International Journal of Obstetrics & Gynaecology. 2019;126(7):907-14. <https://doi.org/10.1111/1471-0528.15611> | Not available |
| Wallström T, Strandberg M, Gemzell-Danielsson K, Pilo C, Jarnbert-Pettersson H, Friman-Mathiasson M, et al. Slow-release vaginal insert of misoprostol versus orally administrated solution of misoprostol for the induction of labour in primiparous term pregnant women: a randomised controlled trial. Bjog. 2019;126(9):1148-55. <https://doi.org/10.1111/1471-0528.15796> | Not available |
| Anger H, Dabash R, Durocher J, Hassanein N, Ononge S, Frye L, et al. The effectiveness and safety of introducing condom‐catheter uterine balloon tamponade for postpartum haemorrhage at secondary level hospitals in Uganda, Egypt and Senegal: a stepped wedge, cluster‐randomised trial. BJOG: An International Journal of Obstetrics & Gynaecology. 2019;126(13):1612-21. <https://doi.org/10.1111/1471-0528.15903> | Not available |
| Groom K, McCowan L, Mackay L, Lee A, Gardener G, Unterscheider J, et al. STRIDER NZAus: a multicentre randomised controlled trial of sildenafil therapy in early‐onset fetal growth restriction. BJOG: An International Journal of Obstetrics & Gynaecology. 2019. <https://doi.org/10.1111/1471-0528.15658> | Pels A, Kenny LC, Alfirevic Z, Baker PN, von Dadelszen P, Gluud C, et al. STRIDER (Sildenafil TheRapy in dismal prognosis early onset fetal growth restriction): an international consortium of randomised placebo-controlled trials. BMC Pregnancy and Childbirth. 2017;17(1):440. <https://doi.org/10.1186/s12884-017-1594-z> |
| Nijman T, van Baaren GJ, van Vliet E, Kok M, Gyselaers W, Porath MM, et al. Cost effectiveness of nifedipine compared with atosiban in the treatment of threatened preterm birth (APOSTEL III trial). Bjog. 2019;126(7):875-83. <https://doi.org/10.1111/1471-0528.15625> | van Vliet EO, Schuit E, Heida KY, Opmeer BC, Kok M, Gyselaers W, et al. Nifedipine versus atosiban in the treatment of threatened preterm labour (Assessment of Perinatal Outcome after Specific Tocolysis in Early Labour: APOSTEL III-Trial). BMC Pregnancy Childbirth. 2014;14:93. <https://doi.org/10.1186/1471-2393-14-93> |
| Akselsson A, Lindgren H, Georgsson S, Pettersson K, Steineck G, Skokic V, et al. Mindfetalness to increase women’s awareness of fetal movements and pregnancy outcomes: a cluster‐randomised controlled trial including 39 865 women. BJOG: An International Journal of Obstetrics & Gynaecology. 2020;127(7):829-37. <https://doi.org/10.1111/1471-0528.16104> | Rådestad I, Akselsson A, Georgsson S, Lindgren H, Pettersson K, Steineck G. Rationale, study protocol and the cluster randomization process in a controlled trial including 40,000 women investigating the effects of mindfetalness. Sex Reprod Healthc. 2016;10:56-61. <https://doi.org/10.1016/j.srhc.2016.10.004> |
| Belloeil V, Tessier Cazeneuve C, Leclercq A, Mercier MB, Legendre G, Corroenne R. Impact of music therapy before first-trimester instrumental termination of pregnancy: a randomised controlled trial. Bjog. 2020;127(6):738-45. <https://doi.org/10.1111/1471-0528.16102> | Not available |
| Ahmed A, Williams DJ, Cheed V, Middleton LJ, Ahmad S, Wang K, et al. Pravastatin for early-onset pre-eclampsia: a randomised, blinded, placebo-controlled trial. Bjog. 2020;127(4):478-88. <https://doi.org/10.1111/1471-0528.16013> | Ahmed A. Clinical Trial Protocol. The StAmP Trial: A Proof of Principle, Double-Blind, Randomised Placebo-Controlled, Multi Centre Trial of pravaStatin to Ameliorate Early Onset Pre-eclampsia. Version 7.0. 2013. [Available at: <https://pmc.ncbi.nlm.nih.gov/articles/instance/7063986/bin/BJO-127-478-s003.pdf>] |
| Bick D, Taylor C, Bhavnani V, Healey A, Seed P, Roberts S, et al. Lifestyle information and commercial weight management groups to support maternal postnatal weight management and positive lifestyle behaviour: the SWAN feasibility randomised controlled trial. BJOG: An International Journal of Obstetrics & Gynaecology. 2020;127(5):636-45. <https://doi.org/10.1111/1471-0528.16043> | SWAN Feasibility Trial. Supporting Women with Postnatal Weight MAnagemeNt. SWAN Feasibility Trial Protocol. Available at:. <https://njl-admin.nihr.ac.uk/document/download/2012000> |
| Wolf H, Brok J, Henriksen T, Greisen G, Salvig J, Pryds O, et al. Antenatal magnesium sulphate for the prevention of cerebral palsy in infants born preterm: a double‐blind, randomised, placebo‐controlled, multi‐centre trial. BJOG: An International Journal of Obstetrics & Gynaecology. 2020;127(10):1217-25. <https://doi.org/10.1111/1471-0528.16239> | Not available |
| Beckmann M, Gibbons K, Flenady V, Kumar S. Induction of labour using prostaglandin E(2) as an inpatient versus balloon catheter as an outpatient: a multicentre randomised controlled trial. Bjog. 2020;127(5):571-9. <https://doi.org/10.1111/1471-0528.16030> | Not available |
| Tan P, Rohani E, Lim M, Win S, Omar S. A randomised trial of caesarean wound coverage: exposed versus dressed. BJOG: An International Journal of Obstetrics & Gynaecology. 2020;127(10):1250-8. <https://doi.org/10.1111/1471-0528.16228> | Not available |
| Ngai FW, Wong PC, Chung KF, Chau PH, Hui PW. Effect of couple‐based cognitive behavioural intervention on prevention of postnatal depression: multisite randomised controlled trial. BJOG: An International Journal of Obstetrics & Gynaecology. 2020;127(4):500-7. <https://doi.org/10.1111/1471-0528.15862> | Ngai FW, Wong PW-C, Chau P-H, Chung K-F. The Effect of a Father Inclusive Psychoeducation Program on Postnatal Depression: a Randomized Controlled Trial: ClinicalTrials.gov; 2022 [Available from: https://cdn.clinicaltrials.gov/large-docs/40/NCT02010840/Prot_SAP_000.pdf. |
| Slade P, West H, Thomson G, Lane S, Spiby H, Edwards R, et al. STRAWB2 (Stress and Wellbeing After Childbirth): a randomised controlled trial of targeted self‐help materials to prevent post‐traumatic stress disorder following childbirth. BJOG: An International Journal of Obstetrics & Gynaecology. 2020;127(7):886-96. <https://doi.org/10.1111/1471-0528.16163> | STRAWB2 team. Preventing Post Traumatic Stress Disorder: the Stress and  Wellbeing after Childbirth Study (STRAWB2). Protocol Version 5.0. Available at: <https://doi.org/10.1186/ISRCTN44832384> |
| Sangkomkamhang U, Kongwattanakul K, Kietpeerakool C, Thinkhamrop J, Wannasiri P, Khunpradit S, et al. Restrictive versus routine episiotomy among Southeast Asian term pregnancies: a multicentre randomised controlled trial. BJOG: An International Journal of Obstetrics & Gynaecology. 2020;127(3):397-403. <https://doi.org/10.1111/1471-0528.15982> | Cannot access protocol |
| Husain S, Allotey J, Drymoussi Z, Wilks M, Fernandez‐Felix B, Whiley A, et al. Effects of oral probiotic supplements on vaginal microbiota during pregnancy: a randomised, double‐blind, placebo‐controlled trial with microbiome analysis. BJOG: An International Journal of Obstetrics & Gynaecology. 2020;127(2):275-84. <https://doi.org/10.1111/1471-0528.15675> | Not available |
| Stegwee S, Voet L, Ben A, Leeuw R, Ven P, Duijnhoven R, et al. Effect of single‐ versus double‐layer uterine closure during caesarean section on postmenstrual spotting (2Close): multicentre, double‐blind, randomised controlled superiority trial. BJOG: An International Journal of Obstetrics & Gynaecology. 2021;128(5):866-78. <https://doi.org/10.1111/1471-0528.16472> | Stegwee SI, Jordans IPM, van der Voet LF, Bongers MY, de Groot CJM, Lambalk CB, et al. Single- versus double-layer closure of the caesarean (uterine) scar in the prevention of gynaecological symptoms in relation to niche development – the 2Close study: a multicentre randomised controlled trial. BMC Pregnancy and Childbirth. 2019;19(1):85. <https://doi.org/10.1186/s12884-019-2221-y> |
| Gurol‐Urganci I, Bidwell P, Sevdalis N, Silverton L, Novis V, Freeman R, et al. Impact of a quality improvement project to reduce the rate of obstetric anal sphincter injury: a multicentre study with a stepped‐wedge design. BJOG: An International Journal of Obstetrics & Gynaecology. 2021;128(3):584-92. <https://doi.org/10.1111/1471-0528.16396> | Bidwell P, Thakar R, Sevdalis N, Silverton L, Novis V, Hellyer A, et al. A multi-centre quality improvement project to reduce the incidence of obstetric anal sphincter injury (OASI): study protocol. BMC Pregnancy and Childbirth. 2018;18(1):331. <https://doi.org/10.1186/s12884-018-1965-0> |
| Tan P, Abdussyukur S, Lim B, Win S, Omar S. Twelve‐hour fasting compared with expedited oral intake in the initial inpatient management of hyperemesis gravidarum: a randomised trial. BJOG: An International Journal of Obstetrics & Gynaecology. 2020;127(11):1430-7. <https://doi.org/10.1111/1471-0528.16290> | Not available |
| Hautakangas T, Uotila J, Huhtala H, Palomäki O. Intrauterine versus external tocodynamometry in monitoring labour: a randomised controlled clinical trial. BJOG: An International Journal of Obstetrics & Gynaecology. 2020;127(13):1677-86. <https://doi.org/10.1111/1471-0528.16343> | Not available |
| Choi SJ, Kwak D, Kil K, Kim SC, Kwon JY, Kim Y, et al. Vaginal compared with intramuscular progestogen for preventing preterm birth in high‐risk pregnant women (VICTORIA study): a multicentre, open‐label randomised trial and meta‐analysis. BJOG: An International Journal of Obstetrics & Gynaecology. 2020;127(13):1646-54. <https://doi.org/10.1111/1471-0528.16365> | Not available |
| Abramowitz L, Mandelbrot L, Bourgeois Moine A, Tohic A, Carne Carnavalet C, Poujade O, et al. Caesarean section in the second delivery to prevent anal incontinence after asymptomatic obstetric anal sphincter injury: the EPIC multicentre randomised trial. BJOG: An International Journal of Obstetrics & Gynaecology. 2021;128(4):685-93. <https://doi.org/10.1111/1471-0528.16452> | Not available |
| Ducloy‐Bouthors A, Mercier F, Grouin J, Bayoumeu F, Corouge J, Le Gouez A, et al. Early and systematic administration of fibrinogen concentrate in postpartum haemorrhage following vaginal delivery: the FIDEL randomised controlled trial. BJOG: An International Journal of Obstetrics & Gynaecology. 2021;128(11):1814-23 <https://doi.org/10.1111/1471-0528.16699>  **A trial protocol was also made available as a supplementary material to the main trial report.** | Ducloy-Bouthors AS, Mignon A, Huissoud C, Grouin JM, Mercier FJ. Fibrinogen concentrate as a treatment for postpartum haemorrhage-induced coagulopathy: A study protocol for a randomised multicentre controlled trial. The fibrinogen in haemorrhage of DELivery (FIDEL) trial. Anaesth Crit Care Pain Med. 2016;35(4):293-8. <https://doi.org/10.1016/j.accpm.2015.10.011> |
| Lori JR, Munro-Kramer ML, Liu H, McGlasson KL, Zhang X, Lee H, et al. Increasing facility delivery through maternity waiting homes for women living far from a health facility in rural Zambia: a quasi-experimental study. Bjog. 2021;128(11):1804-12. <https://doi.org/10.1111/1471-0528.16755> | Scott NA, Kaiser JL, Vian T, Bonawitz R, Fong RM, Ngoma T, et al. Impact of maternity waiting homes on facility delivery among remote households in Zambia: protocol for a quasiexperimental, mixed-methods study. BMJ Open. 2018;8(8):e022224. <https://doi.org/10.1136/bmjopen-2018-022224> |
| Acosta‐Manzano P, Leopold‐Posch B, Simmons D, Devlieger R, Galjaard S, Corcoy R, et al. The unexplored role of sedentary time and physical activity in glucose and lipid metabolism‐related placental mRNAs in pregnant women who are obese: the DALI lifestyle randomised controlled trial. BJOG: An International Journal of Obstetrics &amp; Gynaecology. 2022;129(5):708-21. <https://doi.org/10.1111/1471-0528.16945> | Jelsma JGM, van Poppel MNM, Galjaard S, Desoye G, Corcoy R, Devlieger R, et al. DALI: Vitamin D and lifestyle intervention for gestational diabetes mellitus (GDM) prevention: an European multicentre, randomised trial – study protocol. BMC Pregnancy and Childbirth. 2013;13(1):142. <https://doi.org/10.1186/1471-2393-13-142> |
| Flenady V, Gardener G, Ellwood D, Coory M, Weller M, Warrilow K, et al. My Baby’s Movements: a stepped‐wedge cluster‐randomised controlled trial of a fetal movement awareness intervention to reduce stillbirths. BJOG: An International Journal of Obstetrics &amp; Gynaecology. 2022;129(1):29-41. <https://doi.org/10.1111/1471-0528.16944> | Flenady V, Gardener G, Boyle FM, Callander E, Coory M, East C, et al. My Baby’s Movements: a stepped wedge cluster randomised controlled trial to raise maternal awareness of fetal movements during pregnancy study protocol. BMC Pregnancy and Childbirth. 2019;19(1):430. <https://doi.org/10.1186/s12884-019-2575-1> |
| de Wolff MG, Ladekarl M, Sparholt L, Lykke JA. Rebozo and External Cephalic Version in breech presentation (RECEIVE): A randomised controlled study. Bjog. 2022;129(10):1666-75. <https://doi.org/10.1111/1471-0528.17111> | Not available |
| Alberico S, Erenbourg A, Hod M, Yogev Y, Hadar E, Neri F, et al. Immediate delivery or expectant management in gestational diabetes at term: the GINEXMAL randomised controlled trial. BJOG: An International Journal of Obstetrics & Gynaecology. 2017;124(4):669-77. <https://doi.org/10.1111/1471-0528.14389> | Maso G, Alberico S, Wiesenfeld U, Ronfani L, Erenbourg A, Hadar E, et al. "GINEXMAL RCT: Induction of labour versus expectant management in gestational diabetes pregnancies". BMC Pregnancy Childbirth. 2011;11:31. <https://doi.org/10.1186/1471-2393-11-31> |
| Nelson H, O'Brien S, Burnard S, Mayer M, Alvarez M, Knowlden J, et al. Intramuscular oxytocin versus Syntometrine®versus carbetocin for prevention of primary postpartum haemorrhage after vaginal birth: a randomised double‐blinded clinical trial of effectiveness, side effects and quality of life. BJOG: An International Journal of Obstetrics &amp; Gynaecology. 2021;128(7):1236-46. <https://doi.org/10.1111/1471-0528.16622> | van der Nelson H, O'Brien S, Lenguerrand E, Marques E, Alvarez M, Mayer M, et al. Intramuscular oxytocin versus oxytocin/ergometrine versus carbetocin for prevention of primary postpartum haemorrhage after vaginal birth: study protocol for a randomised controlled trial (the IMox study). Trials. 2019;20(1):4. <https://doi.org/10.1186/s13063-018-3109-2> |
| Bender W, Levine L, Durnwald C. Text Message-Based Breastfeeding Support Compared With Usual Care: A Randomized Controlled Trial. Obstet Gynecol. 2022;140(5):853-60. <https://doi.org/10.1097/aog.0000000000004961> | Not available |
| Chin J, McGrath M, Lokken E, Upegui CD, Prager S, Micks E. Ketamine Compared With Fentanyl for Surgical Abortion: A Randomized Controlled Trial. Obstet Gynecol. 2022;140(3):461-9. <https://doi.org/10.1097/aog.0000000000004903> | [protocol] Ketamine versus Fentanyl for Surgical Abortions: A Randomized Controlled Noninferiority Trial. NCT NUMBER: 04871425: Clinical Trials.gov; 2021 [Available from: <https://cdn.clinicaltrials.gov/large-docs/25/NCT04871425/Prot_SAP_002.pdf>.] |
| Saad AF, Gavara R, Senguttuvan RN, Goncharov AD, Berry M, Eid J, et al. Outpatient Compared With Inpatient Preinduction Cervical Ripening Using a Synthetic Osmotic Dilator: A Randomized Clinical Trial. Obstet Gynecol. 2022;140(4):584-90. <https://doi.org/10.1097/aog.0000000000004942> | Not available |
| Anselem O, Jouannic JM, Winer N, Bouchghoul H, Vivanti AJ, Quibel T, et al. Cervical Dilators Used Concurrently With Misoprostol to Shorten Labor in Second-Trimester Termination of Pregnancy: A Randomized Controlled Trial. Obstet Gynecol. 2022;140(3):453-60. <https://doi.org/10.1097/aog.0000000000004887> | Not available |
| Bruno AM, Allshouse AA, Campbell HM, Branch DW, Lim MY, Silver RM, et al. Weight-Based Compared With Fixed-Dose Enoxaparin Prophylaxis After Cesarean Delivery: A Randomized Controlled Trial. Obstet Gynecol. 2022;140(4):575-83. <https://doi.org/10.1097/aog.0000000000004937> | Not available |
| Masse N, Dexter F, Wong CA. Prophylactic Methylergonovine and Oxytocin Compared With Oxytocin Alone in Patients Undergoing Intrapartum Cesarean Birth: A Randomized Controlled Trial. Obstet Gynecol. 2022;140(2):181-6. <https://doi.org/10.1097/aog.0000000000004857> | Masse N. A Randomized Controlled Trial to Assess the Effectiveness of Multimodal Prophylactic Uterotonics in Patients Undergoing Non-Elective Cesarean Sections after a Trial of Labor [protocol]. NCT Number: 03904446 ClinicalTrials.gov2019 [Available from: <https://cdn.clinicaltrials.gov/large-docs/46/NCT03904446/SAP_001.pdf>. |
| Kim CS, Dragoman M, Porsch L, Markowitz J, Lunde B, Stoffels G, et al. Same-Day Compared With Overnight Cervical Preparation Before Dilation and Evacuation Between 16 and 19 6/7 Weeks of Gestation: A Randomized Controlled Trial. Obstet Gynecol. 2022;139(6):1141-4. <https://doi.org/10.1097/aog.0000000000004790> | Not available |
| Crouthamel B, Economou N, Averbach S, Rible R, Kully G, Meckstroth K, et al. Effect of Paracervical Block Volume on Pain Control for Dilation and Aspiration: A Randomized Controlled Trial. Obstet Gynecol. 2022;140(2):234-42. <https://doi.org/10.1097/aog.0000000000004862> | Effect of Paracervical Block Volume on Pain Control for Dilation and Curettage. [protocol] NCT #03636451: ClinicalTrials.gov; 2022 [Available from: <https://cdn.clinicaltrials.gov/large-docs/51/NCT03636451/Prot_SAP_000.pdf>. |
| Kurata NB, Ghatnekar RJ, Mercer E, Chin JM, Kaneshiro B, Yamasato KS. Transcutaneous Electrical Nerve Stimulation for Post-Cesarean Birth Pain Control: A Randomized Controlled Trial. Obstet Gynecol. 2022;140(2):174-80. <https://doi.org/10.1097/aog.0000000000004798> | Not available |
| Gavara R, Saad AF, Wapner RJ, Saade G, Fu A, Barrow R, et al. Cervical Ripening Efficacy of Synthetic Osmotic Cervical Dilator Compared With Oral Misoprostol at Term: A Randomized Controlled Trial. Obstet Gynecol. 2022;139(6):1083-91. <https://doi.org/10.1097/aog.0000000000004799> | Gavara R, Wapner R, Saad A. Comparison of Misoprostol Ripening Efficacy With Dilapan. Version 3.1 dated September 4, 2020: ClinicalTrials.gov; 2020 [Available from: https://cdn.clinicaltrials.gov/large-docs/36/NCT03670836/Prot_002.pdf. |
| Rolland de Souza AS, Marques de Souza Lima M. Pessary Plus Progesterone to Prevent Preterm Birth in Women With Short Cervixes: A Randomized Controlled Trial. Obstet Gynecol. 2022;139(5):937. <https://doi.org/10.1097/aog.0000000000004784> | Not available |
| Qureshey EJ, Chauhan SP, Wagner SM, Batiste O, Chen HY, Ashimi S, et al. Educational Multimedia Tool Compared With Routine Care for the Uptake of Postpartum Long-Acting Reversible Contraception in Individuals With High-Risk Pregnancies: A Randomized Controlled Trial. Obstet Gynecol. 2022;139(4):571-8. <https://doi.org/10.1097/aog.0000000000004718> | Not available |
| Shaw KA, Lerma K, Hughes T, Hastings C, Fok WK, Blumenthal PD. A Comparison of Paracervical Block Volumes Before Osmotic Dilator Placement: A Randomized Controlled Trial. Obstet Gynecol. 2021;138(3):443-8. <https://doi.org/10.1097/aog.0000000000004485> | Fok WK, Shaw KA, Blumenthal PD. 12 vs. 20 mL paracervical block for pain-control during cervical preparation for dilation and evacuation: A single-blinded randomized controlled trial. NCT03356145 ClinicalTrials.gov; 2018 [Available from: <https://cdn.clinicaltrials.gov/large-docs/45/NCT03356145/Prot_SAP_000.pdf>. |
| Lerma K, Goldthwaite LM, Blumenthal PD, Shaw KA. Transcutaneous Electrical Nerve Stimulation for Pain Management of Aspiration Abortion up to 83 Days of Gestation: A Randomized Controlled Trial. Obstet Gynecol. 2021;138(3):417-25. <https://doi.org/10.1097/aog.0000000000004502> | Transcutaneous Electrical Nerve Stimulation (TENS) for Pain Control During First Trimester Abortion: A Blinded Randomized Controlled Trial . Study Protocol and Statistical Analysis Plan. NCT03187002: ClinicalTrials.gov; 2019 [Available from: <https://cdn.clinicaltrials.gov/large-docs/02/NCT03187002/Prot_SAP_000.pdf>. |
| Subramaniam A, Ye Y, Mbah R, Mbunwe DM, Pekwarake S, Bunwi EY, et al. Single Dose of Oral Azithromycin With or Without Amoxicillin to Prevent Peripartum Infection in Laboring, High-Risk Women in Cameroon: A Randomized Controlled Trial. Obstet Gynecol. 2021;138(5):703-13. <https://doi.org/10.1097/aog.0000000000004565>  **A trial protocol was also made available as a supplementary material to the main trial report.** | Tita AT. Single oral dose of azithromycin1 gm with or without amoxicillin2 gm to prevent peripartum infection and sepsis in laboring high-risk women: 3-Arm RCT. [protocol] MISP Protocol and Procedures. : ClinicalTrials.gov; 2018 [Available from: <https://cdn.clinicaltrials.gov/large-docs/97/NCT03248297/Prot_SAP_000.pdf> |
| Verhaeghe C, Corroenne R, Spiers A, Descamps P, Gascoin G, Bouet PE, et al. Delivery Mode After Manual Rotation of Occiput Posterior Fetal Positions: A Randomized Controlled Trial. Obstet Gynecol. 2021;137(6):999-1006. <https://doi.org/10.1097/aog.0000000000004386> | Verhaeghe C, Parot-Schinkel E, Bouet PE, Madzou S, Biquard F, Gillard P, et al. The impact of manual rotation of the occiput posterior position on spontaneous vaginal delivery rate: study protocol for a randomized clinical trial (RMOS). Trials. 2018;19(1):109. <https://doi.org/10.1186/s13063-018-2497-7> |
| Son M, Roy A, Stetson BT, Grady NT, Vanecko MC, Bond N, et al. High-Dose Compared With Standard-Dose Oxytocin Regimens to Augment Labor in Nulliparous Women: A Randomized Controlled Trial. Obstet Gynecol. 2021;137(6):991-8. <https://doi.org/10.1097/aog.0000000000004399> | Son M RA, Stetson BT, Grady NT, Vanecko MC, Bond N, et al. . PROTOCOL TITLE: A randomized double blind clinical trial comparing oxytocin low-dose and high-dose regimens for labor augmentation. Version 4, 2020. From: High-dose compared with standard-dose oxytocin regimens to augment labor in nulliparous women: a randomized controlled trial. Obstet Gynecol 2021;137. [Available from: <https://cdn-links.lww.com/permalink/aog/c/aog_137_6_2021_04_05_son_21-92_sdc1.pdf> |
| Hawkins JS, Wells CE, Casey BM, McIntire DD, Leveno KJ. Nifedipine for Acute Tocolysis of Preterm Labor: A Placebo-Controlled Randomized Trial. Obstet Gynecol. 2021;138(1):73-8. <https://doi.org/10.1097/aog.0000000000004436> | Wells E, Nelson D, McIntire DD, Leveno KJ. Protocol: Nifedipine for Acute Tocolysis of Preterm Labor. Version 8.0: ClinicalTrials.gov; 2018 [Available from: <https://cdn.clinicaltrials.gov/large-docs/33/NCT02132533/Prot_SAP_000.pdf>. |
| Hamdaoui N, Cardinale C, Fabre C, Baumstarck K, Agostini A. Pain Associated With Cervical Priming for First-Trimester Surgical Abortion: A Randomized Controlled Trial. Obstet Gynecol. 2021;137(6):1055-60. <https://doi.org/10.1097/aog.0000000000004376> | Not available |
| Allanson ER, Copson S, Spilsbury K, Criddle S, Jennings B, Doherty DA, et al. Pretreatment With Mifepristone Compared With Misoprostol Alone for Delivery After Fetal Death Between 14 and 28 Weeks of Gestation: A Randomized Controlled Trial. Obstet Gynecol. 2021;137(5):801-9. <https://doi.org/10.1097/aog.0000000000004344> | Not available |
| Davis EM, Abebe KZ, Simhan HN, Catalano P, Costacou T, Comer D, et al. Perinatal Outcomes of Two Screening Strategies for Gestational Diabetes Mellitus: A Randomized Controlled Trial. Obstet Gynecol. 2021;138(1):6-15. <https://doi.org/10.1097/aog.0000000000004431> | Abebe KZ, Scifres C, Simhan HN, Day N, Catalano P, Bodnar LM, et al. Comparison of Two Screening Strategies for Gestational Diabetes (GDM(2)) Trial: Design and rationale. Contemp Clin Trials. 2017;62:43-9. <https://doi.org/10.1016/j.cct.2017.08.012> |
| Mokhtari NB, Saeed H, Kawakita T, Huang JC, Iqbal SN. Educational Video on Pain Management and Subsequent Opioid Use After Cesarean Delivery: A Randomized Controlled Trial. Obstet Gynecol. 2021;138(2):253-9. <https://doi.org/10.1097/aog.0000000000004468> | Not available |
| Porter P, Muirhead F, Brisbane J, Schneider B, Choveaux J, Bear N, et al. Accuracy, Clinical Utility, and Usability of a Wireless Self-Guided Fetal Heart Rate Monitor. Obstet Gynecol. 2021;137(4):673-81. <https://doi.org/10.1097/aog.0000000000004322> | The HeraBEAT USA™ Trial. Functionality and Acceptability of a Medical Grade, Smartphone based, Fetal Heart Rate Monitor for Self-Administration by Low Risk Pregnant Women. ClinicalTrials.gov. 2021. Available from: <https://cdn.clinicaltrials.gov/large-docs/15/NCT04232215/Prot_SAP_000.pdf> |
| Grossman D, Baba CF, Kaller S, Biggs MA, Raifman S, Gurazada T, et al. Medication Abortion With Pharmacist Dispensing of Mifepristone. Obstet Gynecol. 2021;137(4):613-22. <https://doi.org/10.1097/aog.0000000000004312> | Grossman D. Research Protocol: Alternative Provision of Medication Abortion via Pharmacy Dispensing. : ClinicalTrials.gov; 2020 [Available from: <https://cdn.clinicaltrials.gov/large-docs/57/NCT03320057/Prot_006.pdf>. |
| Balki M, Downey K, Walker A, Seaward G, Carvalho JCA. Prophylactic Administration of Uterotonics to Prevent Postpartum Hemorrhage in Women Undergoing Cesarean Delivery for Arrest of Labor: A Randomized Controlled Trial. Obstet Gynecol. 2021;137(3):505-13. <https://doi.org/10.1097/aog.0000000000004288> | Not available |
| Goldman AR, Porsch L, Hintermeister A, Dragoman M. Transcutaneous Electrical Nerve Stimulation to Reduce Pain With Medication Abortion: A Randomized Controlled Trial. Obstet Gynecol. 2021;137(1):100-7. <https://doi.org/10.1097/aog.0000000000004208> | Not available |
| Kuhlmann MJ, Spencer N, Garcia-Jasso C, Singh P, Abdelwahab M, Vaughn M, et al. Foley Bulb Insertion by Blind Placement Compared With Direct Visualization: A Randomized Controlled Trial. Obstet Gynecol. 2021;137(1):139-45. <https://doi.org/10.1097/aog.0000000000004182> | Not available |
| Brookfield KF, Tuel K, Rincon M, Vinson A, Caughey AB, Carvalho B. Alternate Dosing Protocol for Magnesium Sulfate in Obese Women With Preeclampsia: A Randomized Controlled Trial. Obstet Gynecol. 2020;136(6):1190-4. <https://doi.org/10.1097/aog.0000000000004137> | Protocol Title: A Randomized Study of an Alternate Dosing Protocol for Magnesium Sulfate in Obese Preeclamptic Women: ClinicalTrials.gov; 2015 [Available from: <https://cdn.clinicaltrials.gov/large-docs/39/NCT02835339/Prot_SAP_000.pdf> |
| Adhikari EH, Nelson DB, McIntire DD, Leveno KJ. Foley Bulb Added to an Oral Misoprostol Induction Protocol: A Cluster Randomized Trial. Obstet Gynecol. 2020;136(5):953-61. <https://doi.org/10.1097/aog.0000000000004123> | Adhikari EH. Study Title: Induction of labor with Oral misoprostol alone versus Combined Oral misoprostol with Foley bulb: a Cluster Randomized Trial. NCT03407625 [protocol]: ClinicalTrials.gov; 2017 [Available from: <https://cdn.clinicaltrials.gov/large-docs/25/NCT03407625/Prot_SAP_000.pdf>. |
| Ausbeck EB, Jauk VC, Xue Y, Files P, Kuper SG, Subramaniam A, et al. Outpatient Foley Catheter for Induction of Labor in Nulliparous Women: A Randomized Controlled Trial. Obstet Gynecol. 2020;136(3):597-606. <https://doi.org/10.1097/aog.0000000000004041> | Not available |
| Phipps MG, Ware CF, Stout RL, Raker CA, Zlotnick C. Reducing the Risk for Postpartum Depression in Adolescent Mothers: A Randomized Controlled Trial. Obstet Gynecol. 2020;136(3):613-21. <https://doi.org/10.1097/aog.0000000000004003> | Not available |
| Ganer Herman H, Kleiner I, Tairy D, Gonen N, Ben Zvi M, Kovo M, et al. Effect of Digital Step Counter Feedback on Mobility After Cesarean Delivery: A Randomized Controlled Trial. Obstet Gynecol. 2020;135(6):1345-52. <https://doi.org/10.1097/aog.0000000000003879> | Not available |
| Mireault D, Loubert C, Drolet P, Tordjman L, Godin N, Richebé P, et al. Uterine Exteriorization Compared With In Situ Repair of Hysterotomy After Cesarean Delivery: A Randomized Controlled Trial. Obstet Gynecol. 2020;135(5):1145-51. <https://doi.org/10.1097/aog.0000000000003821> | Not available |
| Blake-Lamb T, Boudreau AA, Matathia S, Perkins ME, Roche B, Cheng ER, et al. Association of the First 1,000 Days Systems-Change Intervention on Maternal Gestational Weight Gain. Obstet Gynecol. 2020;135(5):1047-57. <https://doi.org/10.1097/aog.0000000000003752> | Blake-Lamb T, Boudreau AA, Matathia S, Tiburcio E, Perkins ME, Roche B, et al. Strengthening integration of clinical and public health systems to prevent maternal-child obesity in the First 1,000Days: A Collective Impact approach. Contemp Clin Trials. 2018;65:46-52. <https://doi.org/10.1016/j.cct.2017.12.001> |
| Chen M, Liu X, You Y, Wang X, Li T, Luo H, et al. Internal Iliac Artery Balloon Occlusion for Placenta Previa and Suspected Placenta Accreta: A Randomized Controlled Trial. Obstet Gynecol. 2020;135(5):1112-9. <https://doi.org/10.1097/aog.0000000000003792> | Not available |
| Bleicher I, Dikopoltsev E, Kadour-Ferro E, Sammour R, Gonen R, Sagi S, et al. Double-Balloon Device for 6 Compared With 12 Hours for Cervical Ripening: A Randomized Controlled Trial. Obstet Gynecol. 2020;135(5):1153-60. <https://doi.org/10.1097/aog.0000000000003804> | Not available |
| Colwill AC, Alton K, Bednarek PH, Bayer LL, Jensen JT, Garg B, et al. Cannabinoids for Pain Control During Medical Abortion: A Randomized Controlled Trial. Obstet Gynecol. 2020;135(6):1289-95. <https://doi.org/10.1097/aog.0000000000003850> | Alton K. Official Title: Cannabinoids for Pain Control during Medical Abortion. NCT No. 03604341 [protocol]: ClinicalTrials.gov; 2019 [Available from: <https://cdn.clinicaltrials.gov/large-docs/41/NCT03604341/Prot_SAP_000.pdf> |
| Creinin MD, Hou MY, Dalton L, Steward R, Chen MJ. Mifepristone Antagonization With Progesterone to Prevent Medical Abortion: A Randomized Controlled Trial. Obstet Gynecol. 2020;135(1):158-65. . <https://doi.org/10.1097/aog.0000000000003620> | A Randomized Trial of Mifepristone Antagonization with High-Dose Progesterone to Prevent Medical Abortion. NCT03774745: ClinicalTrials.gov; 2019 [Available from: <https://cdn.clinicaltrials.gov/large-docs/45/NCT03774745/Prot_SAP_000.pdf> |
| Lassey SC, Little SE, Saadeh M, Patton N, Farber MK, Bateman BT, et al. Cephalic Elevation Device for Second-Stage Cesarean Delivery: A Randomized Controlled Trial. Obstet Gynecol. 2020;135(4):879-84. <https://doi.org/10.1097/aog.0000000000003746> | Not available |
| Barney EZ, Pedro CD, Gamez BH, Fuller ME, Dominguez JE, Habib AS. Ropivacaine and Ketorolac Wound Infusion for Post-Cesarean Delivery Analgesia: A Randomized Controlled Trial. Obstet Gynecol. 2020;135(2):427-35. <https://doi.org/10.1097/aog.0000000000003601> | Impact of local anesthetic wound infiltration on postoperative pain following Cesarean delivery. NCT02829944 [protocol] 2016 [Available from: <https://cdn.clinicaltrials.gov/large-docs/44/NCT02829944/Prot_SAP_001.pdf>. |
| Penfield CA, McNulty JA, Oakes MC, Nageotte MP. Ibuprofen and Postpartum Blood Pressure in Women With Hypertensive Disorders of Pregnancy: A Randomized Controlled Trial. Obstet Gynecol. 2019;134(6):1219-26. <https://doi.org/10.1097/aog.0000000000003553> | Penfield CA, McNulty J, Nageotte M. A randomized controlled trial on the effects of NSAIDs on postpartum blood pressure in patients with hypertensive disorders of pregnancy. : ClinicalTrials.gov; 2016 [Available from: <https://cdn.clinicaltrials.gov/large-docs/67/NCT03011567/Prot_SAP_000.pdf>.] |
| Colwill AC, Bayer LL, Bednarek P, Garg B, Jensen JT, Edelman AB. Opioid Analgesia for Medical Abortion: A Randomized Controlled Trial. Obstet Gynecol. 2019;134(6):1163-70. <https://doi.org/10.1097/aog.0000000000003576> | Colwill A. Official Title: Opioid Analgesia for Medical Abortion: A Randomized Controlled Trial [protocol] NCT No: NCT03139240: ClinicalTrials.gov; 2017 [Available from: <https://cdn.clinicaltrials.gov/large-docs/40/NCT03139240/Prot_SAP_000.pdf> |
| Hussamy DJ, Wortman AC, McIntire DD, Leveno KJ, Casey BM, Roberts SW. Closed Incision Negative Pressure Therapy in Morbidly Obese Women Undergoing Cesarean Delivery: A Randomized Controlled Trial. Obstet Gynecol. 2019;134(4):781-9. <https://doi.org/10.1097/aog.0000000000003465> | Not available |
| Braginsky L, Javellana M, Cleveland E, Elue R, Wang C, Boyle D, et al. Tissue Adhesive Compared With Sterile Strips After Cesarean Delivery: A Randomized Controlled Trial. Obstet Gynecol. 2019;134(2):295-301. <https://doi.org/10.1097/aog.0000000000003367> | Tissue Adhesive Compared With Sterile Strips After Cesarean Delivery: A Randomized Controlled Trial [protocol]. NCT02838017: ClinicalTrials.gov; 2017 [Available from: <https://cdn.clinicaltrials.gov/large-docs/17/NCT02838017/Prot_SAP_000.pdf> |
| Gray BA, Hagey JM, Crabtree D, Wynn C, Weber JM, Pieper CF, et al. Gabapentin for Perioperative Pain Management for Uterine Aspiration: A Randomized Controlled Trial. Obstet Gynecol. 2019;134(3):611-9. <https://doi.org/10.1097/aog.0000000000003398> | Gray B, McElligott K, Deans L. Gabapentin as an adjunct to perioperative pain management regimens for uterine aspiration: a randomized controlled trial for a multicenter prospective meta-analysis: ClinicalTrials.gov; 2016 [Available from: <https://cdn.clinicaltrials.gov/large-docs/10/NCT02725710/Prot_SAP_000.pdf>. |
| Manber R, Bei B, Simpson N, Asarnow L, Rangel E, Sit A, et al. Cognitive Behavioral Therapy for Prenatal Insomnia: A Randomized Controlled Trial. Obstet Gynecol. 2019;133(5):911-9. <https://doi.org/10.1097/aog.0000000000003216> | Not publicly accessible |
| Hamel MS, Kanno LM, Has P, Beninati MJ, Rouse DJ, Werner EF. Intrapartum Glucose Management in Women With Gestational Diabetes Mellitus: A Randomized Controlled Trial. Obstet Gynecol. 2019;133(6):1171-7. <https://doi.org/10.1097/aog.0000000000003257> | Not available |
| Dang VQ, Nguyen LK, Pham TD, He YTN, Vu KN, Phan MTN, et al. Pessary Compared With Vaginal Progesterone for the Prevention of Preterm Birth in Women With Twin Pregnancies and Cervical Length Less Than 38 mm: A Randomized Controlled Trial. Obstet Gynecol. 2019;133(3):459-67. <https://doi.org/10.1097/aog.0000000000003136> | Not publicly accessible |
| Whitehouse K, Tschann M, Soon R, Davis J, Micks E, Salcedo J, et al. Effects of Prophylactic Oxytocin on Bleeding Outcomes in Women Undergoing Dilation and Evacuation: A Randomized Controlled Trial. Obstet Gynecol. 2019;133(3):484-91. <https://doi.org/10.1097/aog.0000000000003104> | A randomized double-blinded controlled trial comparing dilation and evacuation outcomes 2 with and without oxytocin use. [protocol] NCT02083809: ClinicalTrials.gov; 2014 [Available from: <https://cdn.clinicaltrials.gov/large-docs/09/NCT02083809/Prot_SAP_000.pdf>. |
| Ashimi Balogun O, Sibai BM, Pedroza C, Blackwell SC, Barrett TL, Chauhan SP. Serial Third-Trimester Ultrasonography Compared With Routine Care in Uncomplicated Pregnancies: A Randomized Controlled Trial. Obstet Gynecol. 2018;132(6):1358-67. <https://doi.org/10.1097/aog.0000000000002970> | Ashimi Balogun O, Roberts R, Hutchinson M, Mendez-Figueroa H, Chauhan SP, Sibai M. Determination of Abnormal Fetal Growth or Amniotic Fluid with Third Trimester Ultrasounds in Uncomplicated Pregnancies: A Randomized Trial (UP Trial). NCT02702999: ClinicalTrials.gov; 2016 [Available from: <https://cdn.clinicaltrials.gov/large-docs/99/NCT02702999/Prot_SAP_000.pdf>. |
| van Kempen LEM, van Teeffelen AS, de Ruigh AA, Oepkes D, Haak MC, van Leeuwen E, et al. Amnioinfusion Compared With No Intervention in Women With Second-Trimester Rupture of Membranes: A Randomized Controlled Trial. Obstet Gynecol. 2019;133(1):129-36. <https://doi.org/10.1097/aog.0000000000003003> | van Teeffelen AS, van der Ham DP, Willekes C, Al Nasiry S, Nijhuis JG, van Kuijk S, et al. Midtrimester preterm prelabour rupture of membranes (PPROM): expectant management or amnioinfusion for improving perinatal outcomes (PPROMEXIL - III trial). BMC Pregnancy Childbirth. 2014;14:128. <https://doi.org/10.1186/1471-2393-14-128> |
| Cowett AA, Ali R, Cooper MA, Evans M, Conzuelo G, Cremer M. Timing of Etonogestrel Implant Insertion After Dilation and Evacuation: A Randomized Controlled Trial. Obstet Gynecol. 2018;131(5):856-62. <https://doi.org/10.1097/aog.0000000000002590> | Cremer M. Immediate vs. delayed insertion of Nexplanon after termination of pregnancy over 14-weeks gestation. MISP Protocol for NAPA study.Version 1. : ClinicalTrials.gov; 2015 [Available from: https://cdn.clinicaltrials.gov/large-docs/19/NCT02037919/Prot_SAP_000.pdf.] |
| Thaxton L, Pitotti J, Espey E, Teal S, Sheeder J, Singh RH. Nitrous Oxide Compared With Intravenous Sedation for Second-Trimester Abortion: A Randomized Controlled Trial. Obstet Gynecol. 2018;132(5):1192-7. <https://doi.org/10.1097/aog.0000000000002915> | Thaxton L, Singh R, Espey E. Nitrous Oxide versus IV Sedation for Anesthesia (NOVIA) [Protocol Version 5.0]: ClinicalTrials.gov; 2017 [Available from: <https://cdn.clinicaltrials.gov/large-docs/90/NCT02755090/Prot_SAP_000.pdf>.] |
| Prabhu M, Clapp MA, McQuaid-Hanson E, Ona S, OʼDonnell T, James K, et al. Liposomal Bupivacaine Block at the Time of Cesarean Delivery to Decrease Postoperative Pain: A Randomized Controlled Trial. Obstet Gynecol. 2018;132(1):70-8.  <https://doi.org/10.1097/aog.0000000000002649> | Prabhu M CM, McQuaid-Hanson E, Ona S, OʼDonnell T, James K, et al. . *Appendix 1. Study Protocol.* Liposomal Bupivacaine Block at the Time of Cesarean Delivery to Decrease Postoperative Pain: A Randomized Controlled Trial. Obstet Gynecol. 2018;132(1):70-8. 2018. <https://doi.org/https://cdn-links.lww.com/permalink/aog/b/aog_132_1_2018_04_24_prabhu_17-2446_sdc1.pdf> |
| Kuper SG, Jauk VC, George DM, Edwards RK, Szychowski JM, Mazzoni SE, et al. Outpatient Foley Catheter for Induction of Labor in Parous Women: A Randomized Controlled Trial. Obstet Gynecol. 2018;132(1):94-101. <https://doi.org/10.1097/aog.0000000000002678> | Kuper SG. Outpatient Foley for Starting Induction of Labor at TErm (OFFSITE); Randomized-Controlled Study Protocol. Study Protocol and Statistical Analysis Plan. NCT02756689. July 30, 2018. Version 1.1: ClinicalTrials.gov; 2018 [Available from: <https://cdn.clinicaltrials.gov/large-docs/89/NCT02756689/Prot_SAP_000.pdf> |
| Cruz-Melguizo S, San-Frutos L, Martínez-Payo C, Ruiz-Antorán B, Adiego-Burgos B, Campillos-Maza JM, et al. Cervical Pessary Compared With Vaginal Progesterone for Preventing Early Preterm Birth: A Randomized Controlled Trial. Obstet Gynecol. 2018;132(4):907-15. <https://doi.org/10.1097/aog.0000000000002884> | Cabrera-García L, Cruz-Melguizo S, Ruiz-Antorán B, Torres F, Velasco A, Martínez-Payo C, et al. Evaluation of two treatment strategies for the prevention of preterm birth in women identified as at risk by ultrasound (PESAPRO Trial): study protocol for a randomized controlled trial. Trials. 2015;16:427. <https://doi.org/10.1186/s13063-015-0964-y> |
| Viteri OA, Alrais MA, Pedroza C, Hutchinson M, Chauhan SP, Blackwell SC, et al. Torsemide for Prevention of Persistent Postpartum Hypertension in Women With Preeclampsia: A Randomized Controlled Trial. Obstet Gynecol. 2018;132(5):1185-91. <https://doi.org/10.1097/aog.0000000000002941> | Viteri OA, et al. . Torsemide for the Prevention of Persistent Postpartum Hypertension in Preeclamptic Women: A Randomized, Placebo-Control trial. Study Protocol. : ClinicalTrials.gov; 2016 [Available from: <https://cdn.clinicaltrials.gov/large-docs/51/NCT02813551/Prot_SAP_000.pdf>. |
| Wihbey KA, Joyce EM, Spalding ZT, Jones HJ, MacKenzie TA, Evans RH, et al. Prophylactic Negative Pressure Wound Therapy and Wound Complication After Cesarean Delivery in Women With Class II or III Obesity: A Randomized Controlled Trial. Obstet Gynecol. 2018;132(2):377-84. <https://doi.org/10.1097/aog.0000000000002744> | Not available |
| Subramaniam A, Blanchard CT, Erickson BK, Szychowski J, Leath CA, Biggio JR, et al. Feasibility of Complete Salpingectomy Compared With Standard Postpartum Tubal Ligation at Cesarean Delivery: A Randomized Controlled Trial. Obstet Gynecol. 2018;132(1):20-7. <https://doi.org/10.1097/aog.0000000000002646> | Not available |
| Osmundson SS, Raymond BL, Kook BT, Lam L, Thompson EB, Schornack LA, et al. Individualized Compared With Standard Postdischarge Oxycodone Prescribing After Cesarean Birth: A Randomized Controlled Trial. Obstet Gynecol. 2018;132(3):624-30. <https://doi.org/10.1097/aog.0000000000002782> | Osmundson S, Richardson M, Lam L. A Randomized Controlled Trial to Reduce Excess Opioids After Cesarean Delivery [protocol]: ClinicalTrials.gov; 2018 [Available from: <https://cdn.clinicaltrials.gov/large-docs/25/NCT03168425/Prot_SAP_000.pdf>. |
| Garcia C, Moskowitz OM, Chisholm CA, Duska LR, Warren AL, Lyons GR, et al. Salpingectomy Compared With Tubal Ligation at Cesarean Delivery: A Randomized Controlled Trial. Obstet Gynecol. 2018;132(1):29-34. <https://doi.org/10.1097/aog.0000000000002674> | Not available |
| Friedlander EB, Soon R, Salcedo J, Davis J, Tschann M, Kaneshiro B. Prophylactic Pregabalin to Decrease Pain During Medication Abortion: A Randomized Controlled Trial. Obstet Gynecol. 2018;132(3):612-8. <https://doi.org/10.1097/aog.0000000000002787> | Not available |
| Kennelly MA, Ainscough K, Lindsay KL, O'Sullivan E, Gibney ER, McCarthy M, et al. Pregnancy Exercise and Nutrition With Smartphone Application Support: A Randomized Controlled Trial. Obstet Gynecol. 2018;131(5):818-26. <https://doi.org/10.1097/aog.0000000000002582> | Kennelly MA, Ainscough K, Lindsay K, Gibney E, Mc Carthy M, McAuliffe FM. Pregnancy, exercise and nutrition research study with smart phone app support (Pears): Study protocol of a randomized controlled trial. Contemp Clin Trials. 2016;46:92-9. <https://doi.org/10.1016/j.cct.2015.11.018> |
| Hermans FJR, Schuit E, Bekker MN, Woiski M, de Boer MA, Sueters M, et al. Cervical Pessary After Arrested Preterm Labor: A Randomized Controlled Trial. Obstet Gynecol. 2018;132(3):741-9. <https://doi.org/10.1097/aog.0000000000002798> | Hermans FJ, Schuit E, Opmeer BC, Oudijk MA, Bekker M, Woiski M, et al. Effectiveness of a cervical pessary for women who did not deliver 48 h after threatened preterm labor (Assessment of perinatal outcome after specific treatment in early labor: Apostel VI trial). BMC Pregnancy Childbirth. 2016;16(1):154. <https://doi.org/10.1186/s12884-016-0935-7> |
| Al-Ibraheemi Z, Brustman L, Bimson BE, Porat N, Rosenn B. Misoprostol With Foley Bulb Compared With Misoprostol Alone for Cervical Ripening: A Randomized Controlled Trial. Obstet Gynecol. 2018;131(1):23-9. <https://doi.org/10.1097/aog.0000000000002403> | Not available |
| Mackeen AD, Durie DE, Lin M, Huls CK, Qureshey E, Paglia MJ, et al. Foley Plus Oxytocin Compared With Oxytocin for Induction After Membrane Rupture: A Randomized Controlled Trial. Obstet Gynecol. 2018;131(1):4-11. <https://doi.org/10.1097/aog.0000000000002374> | Not available |
| Buresch AM, Van Arsdale A, Ferzli M, Sahasrabudhe N, Sun M, Bernstein J, et al. Comparison of Subcuticular Suture Type for Skin Closure After Cesarean Delivery: A Randomized Controlled Trial. Obstet Gynecol. 2017;130(3):521-6. <https://doi.org/10.1097/aog.0000000000002200> | Not available |
| Peccei A, Blake-Lamb T, Rahilly D, Hatoum I, Bryant A. Intensive Prenatal Nutrition Counseling in a Community Health Setting: A Randomized Controlled Trial. Obstet Gynecol. 2017;130(2):423-32. <https://doi.org/10.1097/aog.0000000000002134> | Not available |
| Hughes BL, Gans KM, Raker C, Hipolito ER, Rouse DJ. A Brief Prenatal Intervention of Behavioral Change to Reduce the Risk of Maternal Cytomegalovirus: A Randomized Controlled Trial. Obstet Gynecol. 2017;130(4):726-34. <https://doi.org/10.1097/aog.0000000000002216> | Not available |
| Soon R, Tschann M, Salcedo J, Stevens K, Ahn HJ, Kaneshiro B. Paracervical Block for Laminaria Insertion Before Second-Trimester Abortion: A Randomized Controlled Trial. Obstet Gynecol. 2017;130(2):387-92. <https://doi.org/10.1097/aog.0000000000002149> | Not available |
| Prabhu M, McQuaid-Hanson E, Hopp S, Burns SM, Leffert LR, Landau R, et al. A Shared Decision-Making Intervention to Guide Opioid Prescribing After Cesarean Delivery. Obstet Gynecol. 2017;130(1):42-6. <https://doi.org/10.1097/aog.0000000000002094> | Not available |
| Mendez-Figueroa H, Schuster M, Maggio L, Pedroza C, Chauhan SP, Paglia MJ. Gestational Diabetes Mellitus and Frequency of Blood Glucose Monitoring: A Randomized Controlled Trial. Obstet Gynecol. 2017;130(1):163-70. <https://doi.org/10.1097/aog.0000000000002101> | Not available |
| Schoen CN, Grant G, Berghella V, Hoffman MK, Sciscione A. Intracervical Foley Catheter With and Without Oxytocin for Labor Induction: A Randomized Controlled Trial. Obstetrics & Gynecology. 2017;129(6). <https://journals.lww.com/greenjournal/fulltext/2017/06000/intracervical_foley_catheter_with_and_without.14.aspx> | Not available |
| Carmo L, Braga GC, Ferriani RA, Quintana SM, Vieira CS. Timing of Etonogestrel-Releasing Implants and Growth of Breastfed Infants: A Randomized Controlled Trial. Obstet Gynecol. 2017;130(1):100-7. <https://doi.org/10.1097/aog.0000000000002092> | Not available |
| Facchinetti F, Vergani P, Di Tommaso M, Marozio L, Acaia B, Vicini R, et al. Progestogens for Maintenance Tocolysis in Women With a Short Cervix: A Randomized Controlled Trial. Obstet Gynecol. 2017;130(1):64-70. <https://doi.org/10.1097/aog.0000000000002065> | Not available |
| Daly N, Farren M, McKeating A, OʼKelly R, Stapleton M, Turner MJ. A Medically Supervised Pregnancy Exercise Intervention in Obese Women: A Randomized Controlled Trial. Obstet Gynecol. 2017;130(5):1001-10. <https://doi.org/10.1097/aog.0000000000002267> | Not available |
| Lappen JR, Myers SA, Bolen N, Mercer BM, Chien EKS. Maternal Pulse Pressure and the Risk of Postepidural Complications: A Randomized Controlled Trial. Obstet Gynecol. 2017;130(6):1366-76. <https://doi.org/10.1097/aog.0000000000002326> | Not available |
| Shen X, Li Y, Xu S, Wang N, Fan S, Qin X, et al. Epidural Analgesia During the Second Stage of Labor: A Randomized Controlled Trial. Obstet Gynecol. 2017;130(5):1097-103. <https://doi.org/10.1097/aog.0000000000002306> | Not available |
| Nunes I, Ayres-de-Campos D, Ugwumadu A, Amin P, Banfield P, Nicoll A, et al. Central Fetal Monitoring With and Without Computer Analysis: A Randomized Controlled Trial. Obstet Gynecol. 2017;129(1):83-90. <https://doi.org/10.1097/aog.0000000000001799> | Ayres-de-Campos D, Ugwumadu A, Banfield P, Lynch P, Amin P, Horwell D, et al. A randomised clinical trial of intrapartum fetal monitoring with computer analysis and alerts versus previously available monitoring. BMC Pregnancy and Childbirth. 2010;10(1):71. <https://doi.org/10.1186/1471-2393-10-71> |
| Baumann P, Gotta V, Adzikah S, Bernet V. Accuracy of a Novel Transcutaneous PCO2 and PO2 Sensor with Optical PO2 Measurement in Neonatal Intensive Care: A Single-Centre Prospective Clinical Trial. Neonatology. 2022;119(2):230-7. <https://doi.org/10.1159/000521809> | Not available |
| Kurtom W, Dormishian A, Jain D, Schott A, Aguilar AC, Grieb G, et al. Effect of the Target Range on Arterial Oxygen Saturation Stability in Extremely Premature Infants. Neonatology. 2022;119(5):638-43. <https://doi.org/10.1159/000525271> | Not available |
| Rodríguez-López J, De la Cruz Bértolo J, García-Lara NR, Núñez Vecino JL, Soriano-Ramos M, Asla Elorriaga I, et al. Mother's Bed Incline and Desaturation Episodes in Healthy Term Newborns during Early Skin-to-Skin Contact: A Multicenter Randomized Controlled Trial. Neonatology. 2021;118(6):702-9. <https://doi.org/10.1159/000519387> | Rodríguez López J, García Lara NR, López Maestro M, De la Cruz Bértolo J, Martínez Ávila JC, Vento M, et al. What is the impact of mother’s bed incline on episodes of decreased oxygen saturation in healthy newborns in skin-to-skin contact after delivery: Study protocol for a randomized controlled trial. Trials. 2019;20(1):179. <https://doi.org/10.1186/s13063-019-3256-0> |
| Angoa G, Pronovost E, Ndiaye A, Lavoie PM, Lemyre B, Mohamed I, et al. Effect of Maternal Docosahexaenoic Acid Supplementation on Very Preterm Infant Growth: Secondary Outcome of a Randomized Clinical Trial. Neonatology. 2022;119(3):377-85. <https://doi.org/10.1159/000524147> | Marc I, Piedboeuf B, Lacaze-Masmonteil T, Fraser W, Mâsse B, Mohamed I, et al.  MOBYDIck Trial Supplement 1  Trial Protocol and SAP.  Supplement to Effect of Maternal Docosahexaenoic Acid Supplementation on Bronchopulmonary Dysplasia-Free Survival in Breastfed Preterm Infants: A Randomized Clinical Trial. Jama. 2020;324(2):157-67. <https://doi.org/10.1001/jama.2020.8896>. Available at: <https://pmc.ncbi.nlm.nih.gov/articles/instance/7361648/bin/jama-324-157-s001.pdf> |
| Treussart C, Decobert F, Tauzin M, Bourgoin L, Danan C, Dassieu G, et al. Patient-Ventilator Synchrony in Extremely Premature Neonates during Non-Invasive Neurally Adjusted Ventilatory Assist or Synchronized Intermittent Positive Airway Pressure: A Randomized Crossover Pilot Trial. Neonatology. 2022;119(3):386-93. <https://doi.org/10.1159/000524327> | Treussart C, Decobert F, Tauzin M, Bourgoin L, Danan C, Dassieu G, et al. Supplementary Material for: Patient-Ventilator Synchrony in Extremely Premature Neonates during Non-Invasive Neurally Adjusted Ventilatory Assist or Synchronized Intermittent Positive Airway Pressure: A Randomized Crossover Pilot. Neonatology. 2022;119(3):386-93. <https://doi.org/10.1159/000524327> |
| Zhu X, Feng Z, Liu C, Shi L, Shi Y, Ramanathan R. Nasal High-Frequency Oscillatory Ventilation in Preterm Infants with Moderate Respiratory Distress Syndrome: A Multicenter Randomized Clinical Trial. Neonatology. 2021;118(3):325-31. <https://doi.org/10.1159/000515226> | Zhu X-W, Shi Y, Shi L-P, Liu L, Xue J, Ramanathan R, et al. Non-invasive high-frequency oscillatory ventilation versus nasal continuous positive airway pressure in preterm infants with respiratory distress syndrome: Study protocol for a multi-center prospective randomized controlled trial. Trials. 2018;19(1):319. <https://doi.org/10.1186/s13063-018-2673-9> |
| Schindler T, Smyth J, Bolisetty S, Michalowski J, Mallitt KA, Singla A, et al. Early PARacetamol (EPAR) Trial: A Randomized Controlled Trial of Early Paracetamol to Promote Closure of the Ductus Arteriosus in Preterm Infants. Neonatology. 2021;118(3):274-81. <https://doi.org/10.1159/000515415> | Schindler T, Smyth J, Bolisetty S, Michalowski J, Lui K. Early PARacetamol (EPAR) trial: a study protocol for a randomised controlled trial of early paracetamol to promote closure of the ductus arteriosus in preterm infants. BMJ Open. 2019;9(10):e031428. <https://doi.org/10.1136/bmjopen-2019-031428> |
| Bach KP, Kuschel CA, Patterson N, Skwish H, Huth S, Phua HH, et al. Effect of Bias Gas Flow on Tracheal Cytokine Concentrations in Ventilated Extremely Preterm Infants: A Randomized Controlled Trial. Neonatology. 2021;118(3):332-9. <https://doi.org/10.1159/000515364> | Not available |
| Kallio M, Mahlman M, Koskela U, Aikio O, Suo-Palosaari M, Pokka T, et al. NIV NAVA versus Nasal CPAP in Premature Infants: A Randomized Clinical Trial. Neonatology. 2019;116(4):380-4. <https://doi.org/10.1159/000502341> | Not available |
| Nunes CR, Procianoy RS, Corso AL, Silveira RC. Use of Azithromycin for the Prevention of Lung Injury in Mechanically Ventilated Preterm Neonates: A Randomized Controlled Trial. Neonatology. 2020;117(4):522-8. <https://doi.org/10.1159/000509462> | Not available |
| Estay AS, Mariani GL, Alvarez CA, Milet B, Agost D, Avila CP, et al. Randomized Controlled Trial of Nonsynchronized Nasal Intermittent Positive Pressure Ventilation versus Nasal CPAP after Extubation of VLBW Infants. Neonatology. 2020;117(2):193-9. <https://doi.org/10.1159/000506164> | Not available |
| Rana N, Kc A, Målqvist M, Subedi K, Andersson O. Effect of Delayed Cord Clamping of Term Babies on Neurodevelopment at 12 Months: A Randomized Controlled Trial. Neonatology. 2019;115(1):36-42. <https://doi.org/10.1159/000491994> | Not available |
| Nuñez-Ramiro A, Benavente-Fernández I, Valverde E, Cordeiro M, Blanco D, Boix H, et al. Topiramate plus Cooling for Hypoxic-Ischemic Encephalopathy: A Randomized, Controlled, Multicenter, Double-Blinded Trial. Neonatology. 2019;116(1):76-84. <https://doi.org/10.1159/000499084> | Not available |
| Bremond-Gignac D, Jacqz-Aigrain E, Abdoul H, Daruich A, Beresniak A, Baud O, et al. Ophthalmic Insert versus Eye Drops for Mydriasis in Neonates: A Randomized Clinical Trial. Neonatology. 2019;115(2):142-8. <https://doi.org/10.1159/000493723> | Not available |
| Nangia S, Vadivel V, Thukral A, Saili A. Early Total Enteral Feeding versus Conventional Enteral Feeding in Stable Very-Low-Birth-Weight Infants: A Randomised Controlled Trial. Neonatology. 2019;115(3):256-62. <https://doi.org/10.1159/000496015> | Not available |
| Cavallin F, Galeazzo B, Loretelli V, Madella S, Pizzolato M, Visentin S, et al. Delayed Cord Clamping versus Early Cord Clamping in Elective Cesarean Section: A Randomized Controlled Trial. Neonatology. 2019;116(3):252-9. <https://doi.org/10.1159/000500325> | Not available |
| Murki S, Singh J, Khant C, Kumar Dash S, Oleti TP, Joy P, et al. High-Flow Nasal Cannula versus Nasal Continuous Positive Airway Pressure for Primary Respiratory Support in Preterm Infants with Respiratory Distress: A Randomized Controlled Trial. Neonatology. 2018;113(3):235-41. <https://doi.org/10.1159/000484400> | Not available |
| Bensouda B, Mandel R, Mejri A, Lachapelle J, St-Hilaire M, Ali N. Temperature Probe Placement during Preterm Infant Resuscitation: A Randomised Trial. Neonatology. 2018;113(1):27-32. <https://doi.org/10.1159/000480537> | Not available |
| Strunk T, Pupala S, Hibbert J, Doherty D, Patole S. Topical Coconut Oil in Very Preterm Infants: An Open-Label Randomised Controlled Trial. Neonatology. 2018;113(2):146-51. <https://doi.org/10.1159/000480538> | Not available |
| Beker F, Opie G, Noble E, Jiang Y, Bloomfield FH. Smell and Taste to Improve Nutrition in Very Preterm Infants: A Randomized Controlled Pilot Trial. Neonatology. 2017;111(3):260-6. <https://doi.org/10.1159/000450883> | Not available |
| Wanous AA, Wey A, Rudser KD, Roberts KD. Feasibility of Laryngeal Mask Airway Device Placement in Neonates. Neonatology. 2017;111(3):222-7. <https://doi.org/10.1159/000450691> | Not available |
| Roofthooft DWE, Simons SHP, van Lingen RA, Tibboel D, van den Anker JN, Reiss IKH, et al. Randomized Controlled Trial Comparing Different Single Doses of Intravenous Paracetamol for Placement of Peripherally Inserted Central Catheters in Preterm Infants. Neonatology. 2017;112(2):150-8. <https://doi.org/10.1159/000468975> | Not available |
| Ibrahim T, Li Wei C, Bautista D, Sriram B, Xiangzhen Fay L, Rajadurai VS. Saline Enemas versus Glycerin Suppositories to Promote Enteral Feeding in Premature Infants: A Pilot Randomized Controlled Trial. Neonatology. 2017;112(4):347-53. <https://doi.org/10.1159/000477999> | Not available |
| Lin YJ, Chen CM, Rehan VK, Florens A, Wu SY, Tsai ML, et al. Randomized Trial to Compare Renal Function and Ductal Response between Indomethacin and Ibuprofen Treatment in Extremely Low Birth Weight Infants. Neonatology. 2017;111(3):195-202. <https://doi.org/10.1159/000450822> | Not available |
| Clements J, Christensen PM, Meyer M. A randomised trial comparing weaning from CPAP alone with weaning using heated humidified high flow nasal cannula in very preterm infants: the CHiPS study. Arch Dis Child Fetal Neonatal Ed. 2022;108(1):63-8. <https://doi.org/10.1136/archdischild-2021-323636> | Not available |
| Nair V, Lal MK, Gillone J, Kannan Loganathan P, Bachman TE. Comparison of volume guarantee and volume-controlled ventilation both using closed loop inspired oxygen in preterm infants: a randomised crossover study (CLIO-VG study). Archives of Disease in Childhood - Fetal and Neonatal Edition. 2022;107(2):161. <https://doi.org/10.1136/archdischild-2021-321712> | Not available |
| Heo JS, Kim EK, Kim SY, Song IG, Yoon YM, Cho H, et al. Direct swallowing training and oral sensorimotor stimulation in preterm infants: a randomised controlled trial. Arch Dis Child Fetal Neonatal Ed. 2022;107(2):166-73. <https://doi.org/10.1136/archdischild-2021-321945> | Kim E-K. The effects of direct swallowing training and oral sensorimotor stimulation in preterm infants [ Protocol for Clinical Trial ]. NCT02508571: ClinicalTrials.gov; 2015 [Available from: https://cdn.clinicaltrials.gov/large-docs/71/NCT02508571/Prot_SAP_000.pdf. |
| Sharma A, Kulkarni S, Thukral A, Sankar MJ, Agarwal R, Deorari AK, et al. Aqueous chlorhexidine 1% versus 2% for neonatal skin antisepsis: a randomised non-inferiority trial. Arch Dis Child Fetal Neonatal Ed. 2021;106(6):643-8. <https://doi.org/10.1136/archdischild-2020-321174> | Not available |
| Murphy MC, Jenkinson A, Coveney J, McCarthy LK, CPF OD. Randomised study of heart rate measurement in preterm newborns with ECG plus pulse oximetry versus oximetry alone. Arch Dis Child Fetal Neonatal Ed. 2021;106(4):438-41. <https://doi.org/10.1136/archdischild-2020-320892> | Not available |
| Falk M, Gunnarsdottir K, Baldursdottir S, Donaldsson S, Jonsson B, Drevhammar T. Interface leakage during neonatal CPAP treatment: a randomised, cross-over trial. Arch Dis Child Fetal Neonatal Ed. 2021;106(6):663-7. <https://doi.org/10.1136/archdischild-2021-321579> | Not available |
| Dijkman KP, Mohns T, Dieleman JP, van Pul C, Goos TG, Reiss IK, et al. Predictive Intelligent Control of Oxygenation (PRICO) in preterm infants on high flow nasal cannula support: a randomised cross-over study. Arch Dis Child Fetal Neonatal Ed. 2021;106(6):621-6. <https://doi.org/10.1136/archdischild-2020-320728> | Not available |
| Dargaville PA, Marshall AP, Ladlow OJ, Bannink C, Jayakar R, Eastwood-Sutherland C, et al. Automated control of oxygen titration in preterm infants on non-invasive respiratory support. Arch Dis Child Fetal Neonatal Ed. 2022;107(1):39-44. <https://doi.org/10.1136/archdischild-2020-321538> | Not available |
| Salverda HH, Cramer SJE, Witlox R, Gale TJ, Dargaville PA, Pauws SC, et al. Comparison of two devices for automated oxygen control in preterm infants: a randomised crossover trial. Arch Dis Child Fetal Neonatal Ed. 2022;107(1):20-5. <https://doi.org/10.1136/archdischild-2020-321387> | Not available |
| Cavallin F, Doglioni N, Allodi A, Battajon N, Vedovato S, Capasso L, et al. Thermal management with and without servo-controlled system in preterm infants immediately after birth: a multicentre, randomised controlled study. Arch Dis Child Fetal Neonatal Ed. 2021;106(6):572-7. <https://doi.org/10.1136/archdischild-2020-320567> | Not available |
| Jardine L, Lui K, Liley HG, Schindler T, Fink J, Asselin J, et al. Trial of aerosolised surfactant for preterm infants with respiratory distress syndrome. Arch Dis Child Fetal Neonatal Ed. 2022;107(1):51-5. <https://doi.org/10.1136/archdischild-2021-321645> | Not available |
| Dempsey EM, Barrington KJ, Marlow N, O'Donnell CPF, Miletin J, Naulaers G, et al. Hypotension in Preterm Infants (HIP) randomised trial. Arch Dis Child Fetal Neonatal Ed. 2021;106(4):398-403. <https://doi.org/10.1136/archdischild-2020-320241> | Not available |
| Danielo Jouhier M, Boscher C, Roze JC, Cailleau N, Chaligne F, Legrand A, et al. Osteopathic manipulative treatment to improve exclusive breast feeding at 1 month. Arch Dis Child Fetal Neonatal Ed. 2021;106(6):591-5. <https://doi.org/10.1136/archdischild-2020-319219> | Not available |
| Kannan Loganathan P, O'Shea JE, Harikumar C, Brigham JC, Rabi Y, Gupta S. Effect of opaque wraps for pulse oximeter sensors: randomised cross-over trial. Arch Dis Child Fetal Neonatal Ed. 2021;106(1):57-61. <https://doi.org/10.1136/archdischild-2020-319049> | Not available |
| Viscardi RM, Terrin ML, Magder LS, Davis NL, Dulkerian SJ, Waites KB, et al. Randomised trial of azithromycin to eradicate Ureaplasma in preterm infants. Arch Dis Child Fetal Neonatal Ed. 2020;105(6):615-22. <https://doi.org/10.1136/archdischild-2019-318122> | Viscardi RM. Azithromycin to Prevent Bronchopulmonary Dsyplasia in Ureaplasmainfected Preterms. A Phase IIb randomized, placebo-controlled, double-blind trial of azithromycin to eradicate Ureaplasma respiratory tract infection in preterm infants [protocol]. Version 5.0: ClinicalTrails.gov; 2017 [Available from: <https://cdn.clinicaltrials.gov/large-docs/34/NCT01778634/Prot_SAP_000.pdf>. |
| de Kort EHM, Prins SA, Reiss IKM, Willemsen SP, Andriessen P, van Weissenbruch MM, et al. Propofol for endotracheal intubation in neonates: a dose-finding trial. Arch Dis Child Fetal Neonatal Ed. 2020;105(5):489-95. <https://doi.org/10.1136/archdischild-2019-318474> | Not available |
| Rochon ME, Lodygensky G, Tabone L, Essouri S, Morneau S, Sinderby C, et al. Continuous neurally adjusted ventilation: a feasibility study in preterm infants. Arch Dis Child Fetal Neonatal Ed. 2020;105(6):640-5. <https://doi.org/10.1136/archdischild-2019-318660> | Not available |
| Jensen EA, Zhang H, Feng R, Dysart K, Nilan K, Munson DA, et al. Individualising care in severe bronchopulmonary dysplasia: a series of N-of-1 trials comparing transpyloric and gastric feeding. Arch Dis Child Fetal Neonatal Ed. 2020;105(4):399-404. <https://doi.org/10.1136/archdischild-2019-317148> | Not available |
| Schwarz CE, Kidszun A, Bieder NS, Franz AR, König J, Mildenberger E, et al. Is faster better? A randomised crossover study comparing algorithms for closed-loop automatic oxygen control. Arch Dis Child Fetal Neonatal Ed. 2020;105(4):369-74. <https://doi.org/10.1136/archdischild-2019-317029> | Not available |
| Aker K, Støen R, Eikenes L, Martinez-Biarge M, Nakken I, Håberg AK, et al. Therapeutic hypothermia for neonatal hypoxic-ischaemic encephalopathy in India (THIN study): a randomised controlled trial. Arch Dis Child Fetal Neonatal Ed. 2020;105(4):405-11. <https://doi.org/10.1136/archdischild-2019-317311> | Not available |
| Pereira SS, Sinha AK, Morris JK, Wertheim DF, Shah DK, Kempley ST. Blood pressure intervention levels in preterm infants: pilot randomised trial. Arch Dis Child Fetal Neonatal Ed. 2019;104(3):F298-f305. <https://doi.org/10.1136/archdischild-2017-314159> | Not available |
| Abiramalatha T, Mathew SK, Mathew BS, Shabeer MP, Arulappan G, Kumar M, et al. Continuous infusion versus intermittent bolus doses of fentanyl for analgesia and sedation in neonates: an open-label randomised controlled trial. Arch Dis Child Fetal Neonatal Ed. 2019;104(4):F433-f9. <https://doi.org/10.1136/archdischild-2018-315345> | Not available |
| Vain NE, Satragno DS, Gordillo JE, Fernandez AL, Carrolli G, Romero NP, et al. Postpartum use of oxytocin and volume of placental transfusion: a randomised controlled trial. Arch Dis Child Fetal Neonatal Ed. 2020;105(1):14-7. <https://doi.org/10.1136/archdischild-2018-316649> | Not available |
| Minocchieri S, Berry CA, Pillow JJ. Nebulised surfactant to reduce severity of respiratory distress: a blinded, parallel, randomised controlled trial. Arch Dis Child Fetal Neonatal Ed. 2019;104(3):F313-f9. <https://doi.org/10.1136/archdischild-2018-315051> | Not available |
| Reynolds PR, Miller TL, Volakis LI, Holland N, Dungan GC, Roehr CC, et al. Randomised cross-over study of automated oxygen control for preterm infants receiving nasal high flow. Arch Dis Child Fetal Neonatal Ed. 2019;104(4):F366-f71. <https://doi.org/10.1136/archdischild-2018-315342> | Not available |
| Liew Z, Fenton AC, Harigopal S, Gopalakaje S, Brodlie M, O'Brien CJ. Physiological effects of high-flow nasal cannula therapy in preterm infants. Arch Dis Child Fetal Neonatal Ed. 2020;105(1):87-93. <https://doi.org/10.1136/archdischild-2018-316773> | Not available |
| El-Naggar W, Simpson D, Hussain A, Armson A, Dodds L, Warren A, et al. Cord milking versus immediate clamping in preterm infants: a randomised controlled trial. Arch Dis Child Fetal Neonatal Ed. 2019;104(2):F145-f50. <https://doi.org/10.1136/archdischild-2018-314757> | Not available |
| Dekker J, Lopriore E, van Zanten HA, Tan R, Hooper SB, Te Pas AB. Sedation during minimal invasive surfactant therapy: a randomised controlled trial. Arch Dis Child Fetal Neonatal Ed. 2019;104(4):F378-f83. <https://doi.org/10.1136/archdischild-2018-315015> | Not available |
| de Vries LS, Groenendaal F, Liem KD, Heep A, Brouwer AJ, van 't Verlaat E, et al. Treatment thresholds for intervention in posthaemorrhagic ventricular dilation: a randomised controlled trial. Arch Dis Child Fetal Neonatal Ed. 2019;104(1):F70-f5. <https://doi.org/10.1136/archdischild-2017-314206> | Not available |
| Klotz D, Schneider H, Schumann S, Mayer B, Fuchs H. Non-invasive high-frequency oscillatory ventilation in preterm infants: a randomised controlled cross-over trial. Arch Dis Child Fetal Neonatal Ed. 2018;103(4):F1-f5. <https://doi.org/10.1136/archdischild-2017-313190> | Not available |
| Gill I, Stafford A, Murphy MC, Geoghegan AR, Crealey M, Laffan E, et al. Randomised trial of estimating oral endotracheal tube insertion depth in newborns using weight or vocal cord guide. Arch Dis Child Fetal Neonatal Ed. 2018;103(4):F312-f6. <https://doi.org/10.1136/archdischild-2017-312798> | Not available |
| Schmölzer GM, M OR, Fray C, van Os S, Cheung PY. Chest compression during sustained inflation versus 3:1 chest compression:ventilation ratio during neonatal cardiopulmonary resuscitation: a randomised feasibility trial. Arch Dis Child Fetal Neonatal Ed. 2018;103(5):F455-f60. <https://doi.org/10.1136/archdischild-2017-313037> | Not available |
| Milési C, Baleine J, Mura T, Benito-Castro F, Ferragu F, Thiriez G, et al. Nasal midazolam vs ketamine for neonatal intubation in the delivery room: a randomised trial. Arch Dis Child Fetal Neonatal Ed. 2018;103(3):F221-f6. <https://doi.org/10.1136/archdischild-2017-312808> | Not available |
| Kieran EA, O'Sullivan A, Miletin J, Twomey AR, Knowles SJ, O'Donnell CPF. 2% chlorhexidine-70% isopropyl alcohol versus 10% povidone-iodine for insertion site cleaning before central line insertion in preterm infants: a randomised trial. Arch Dis Child Fetal Neonatal Ed. 2018;103(2):F101-f6. <https://doi.org/10.1136/archdischild-2016-312193> | Not available |
| Sanghvi KP, Kabra NS, Padhi P, Singh U, Dash SK, Avasthi BS. Prophylactic propranolol for prevention of ROP and visual outcome at 1 year (PreROP trial). Arch Dis Child Fetal Neonatal Ed. 2017;102(5):F389-f94. <https://doi.org/10.1136/archdischild-2016-311548> | Not available |
| Ibrahim NR, Kheng TH, Nasir A, Ramli N, Foo JLK, Syed Alwi SH, et al. Two-hourly versus 3-hourly feeding for very low birthweight infants: a randomised controlled trial. Arch Dis Child Fetal Neonatal Ed. 2017;102(3):F225-f9. <https://doi.org/10.1136/archdischild-2015-310246> | Not available |
| Duley L, Dorling J, Pushpa-Rajah A, Oddie SJ, Yoxall CW, Schoonakker B, et al. Randomised trial of cord clamping and initial stabilisation at very preterm birth. Arch Dis Child Fetal Neonatal Ed. 2018;103(1):F6-f14. <https://doi.org/10.1136/archdischild-2016-312567> | Bradshaw LE, Pushpa-Rajah A, Dorling J, Mitchell EJ, Duley L. Cord pilot trial: update to randomised trial protocol. Trials. 2015;16:407. <https://doi.org/10.1186/s13063-015-0936-2> |
| Glackin SJ, O'Sullivan A, George S, Semberova J, Miletin J. High flow nasal cannula versus NCPAP, duration to full oral feeds in preterm infants: a randomised controlled trial. Arch Dis Child Fetal Neonatal Ed. 2017;102(4):F329-f32. <https://doi.org/10.1136/archdischild-2016-311388> | Not available |
| Edwards AD, Redshaw ME, Kennea N, Rivero-Arias O, Gonzales-Cinca N, Nongena P, et al. Effect of MRI on preterm infants and their families: a randomised trial with nested diagnostic and economic evaluation. Arch Dis Child Fetal Neonatal Ed. 2018;103(1):F15-f21. <https://doi.org/10.1136/archdischild-2017-313102> | Not available |
| Sweet DG, Turner MA, Straňák Z, Plavka R, Clarke P, Stenson BJ, et al. A first-in-human clinical study of a new SP-B and SP-C enriched synthetic surfactant (CHF5633) in preterm babies with respiratory distress syndrome. Arch Dis Child Fetal Neonatal Ed. 2017;102(6):F497-f503. <https://doi.org/10.1136/archdischild-2017-312722> | Not available |
| Ngan AY, Cheung PY, Hudson-Mason A, O'Reilly M, van Os S, Kumar M, et al. Using exhaled CO(2) to guide initial respiratory support at birth: a randomised controlled trial. Arch Dis Child Fetal Neonatal Ed. 2017;102(6):F525-f31. <https://doi.org/10.1136/archdischild-2016-312286> | Not available |
| lottier GK, Wheeler KI, Ali SK, Fathabadi OS, Jayakar R, Gale TJ, et al. Clinical evaluation of a novel adaptive algorithm for automated control of oxygen therapy in preterm infants on non-invasive respiratory support. Arch Dis Child Fetal Neonatal Ed. 2017;102(1):F37-f43. <https://doi.org/10.1136/archdischild-2016-310647> | Not available |
| Baddock SA, Tipene-Leach D, Williams SM, Tangiora A, Jones R, Iosua E, et al. Wahakura Versus Bassinet for Safe Infant Sleep: A Randomized Trial. Pediatrics. 2017;139(2). <https://doi.org/10.1542/peds.2016-0162> | Tipene-Leach D, Baddock S, Williams S, Jones R, Tangiora A, Abel S, et al. Methodology and recruitment for a randomised controlled trial to evaluate the safety of wahakurafor infant bedsharing. BMC Pediatrics. 2014;14(1):240. <https://doi.org/10.1186/1471-2431-14-240> |
| Oei JL, Saugstad OD, Lui K, Wright IM, Smyth JP, Craven P, et al. Targeted Oxygen in the Resuscitation of Preterm Infants, a Randomized Clinical Trial. Pediatrics. 2017;139(1). <https://doi.org/10.1542/peds.2016-1452> | Not available |
| Gras-Le Guen C, Caille A, Launay E, Boscher C, Godon N, Savagner C, et al. Dry Care Versus Antiseptics for Umbilical Cord Care: A Cluster Randomized Trial. Pediatrics. 2017;139(1). <https://doi.org/10.1542/peds.2016-1857> | Not available |
| Taylor BJ, Gray AR, Galland BC, Heath A-LM, Lawrence J, Sayers RM, et al. Targeting Sleep, Food, and Activity in Infants for Obesity Prevention: An RCT. Pediatrics. 2017;139(3). <https://doi.org/10.1542/peds.2016-2037> | Taylor BJ, Heath A-LM, Galland BC, Gray AR, Lawrence JA, Sayers RM, et al. Prevention of Overweight in Infancy (POI.nz) study: a randomised controlled trial of sleep, food and activity interventions for preventing overweight from birth. BMC Public Health. 2011;11(1):942. <https://doi.org/10.1186/1471-2458-11-942> |
| Washio Y, Humphreys M, Colchado E, Sierra-Ortiz M, Zhang Z, Collins BN, et al. Incentive-based Intervention to Maintain Breastfeeding Among Low-income Puerto Rican Mothers. Pediatrics. 2017;139(3):e20163119. <https://doi.org/10.1542/peds.2016-3119> | Not available |
| Williams FLR, Ogston S, Hume R, Watson J, Stanbury K, Willatts P, et al. Supplemental Iodide for Preterm Infants and Developmental Outcomes at 2 Years: An RCT. Pediatrics. 2017;139(5). <https://doi.org/10.1542/peds.2016-3703> | Williams F, Hume R, Ogston S, Brocklehurst P, Morgan K, Juszczak E. A summary of the iodine supplementation study protocol (I2S2): a UK multicentre randomised controlled trial in preterm infants. Neonatology. 2014;105(4):282-9. <https://doi.org/10.1159/000358247-> ***could not access- was not included in review*** |
| Galderisi A, Facchinetti A, Steil GM, Ortiz-Rubio P, Cavallin F, Tamborlane WV, et al. Continuous Glucose Monitoring in Very Preterm Infants: A Randomized Controlled Trial. Pediatrics. 2017;140(4). <https://doi.org/10.1542/peds.2017-1162> | Not available |
| Glanz JM, Wagner NM, Narwaney KJ, Kraus CR, Shoup JA, Xu S, et al. Web-based Social Media Intervention to Increase Vaccine Acceptance: A Randomized Controlled Trial. Pediatrics. 2017;140(6). <https://doi.org/10.1542/peds.2017-1117> | Not available |
| Sharara-Chami R, Lakissian Z, Charafeddine L, Milad N, El-Hout Y. Combination Analgesia for Neonatal Circumcision: A Randomized Controlled Trial. Pediatrics. 2017;140(6). <https://doi.org/10.1542/peds.2017-1935> | Not available |
| Trang S, Zupancic JAF, Unger S, Kiss A, Bando N, Wong S, et al. Cost-Effectiveness of Supplemental Donor Milk Versus Formula for Very Low Birth Weight Infants. Pediatrics. 2018;141(3). <https://doi.org/10.1542/peds.2017-0737> | Unger S, Gibbins S, Zupancic J, O'Connor DL. DoMINO: Donor milk for improved neurodevelopmental outcomes. BMC Pediatr. 2014;14:123. <https://doi.org/10.1186/1471-2431-14-123> |
| Cavallin F, Segafredo G, Pizzol D, Massavon W, Lusiani M, Wingi O, et al. Thermal Effect of a Woolen Cap in Low Birth Weight Infants During Kangaroo Care. Pediatrics. 2018;141(6). <https://doi.org/10.1542/peds.2017-3073> | Trevisanuto D, Putoto G, Pizzol D, Serena T, Manenti F, Varano S, et al. Is a woolen cap effective in maintaining normothermia in low-birth-weight infants during kangaroo mother care? Study protocol for a randomized controlled trial. Trials. 2016;17(1):265. <https://doi.org/10.1186/s13063-016-1387-0> |
| Bembich S, Cont G, Causin E, Paviotti G, Marzari P, Demarini S. Infant Analgesia With a Combination of Breast Milk, Glucose, or Maternal Holding. Pediatrics. 2018;142(3). <https://doi.org/10.1542/peds.2017-3416> | Not available |
| Goldfeld S, Price A, Smith C, Bruce T, Bryson H, Mensah F, et al. Nurse Home Visiting for Families Experiencing Adversity: A Randomized Trial. Pediatrics. 2019;143(1). <https://doi.org/10.1542/peds.2018-1206> | Goldfeld S, Price A, Bryson H, Bruce T, Mensah F, Orsini F, et al. ‘right@home’: a randomised controlled trial of sustained nurse home visiting from pregnancy to child age 2 years, versus usual care, to improve parent care, parent responsivity and the home learning environment at 2 years. BMJ Open. 2017;7(3):e013307. <https://doi.org/10.1136/bmjopen-2016-013307> |
| Kumar J, Dutta S, Sundaram V, Saini SS, Sharma RR, Varma N. Platelet Transfusion for PDA Closure in Preterm Infants: A Randomized Controlled Trial. Pediatrics. 2019;143(5). <https://doi.org/10.1542/peds.2018-2565> | Not available |
| Sharpe C, Reiner GE, Davis SL, Nespeca M, Gold JJ, Rasmussen M, et al. Levetiracetam Versus Phenobarbital for Neonatal Seizures: A Randomized Controlled Trial. Pediatrics. 2020;145(6). <https://doi.org/10.1542/peds.2019-3182> | Haas R. University of California, San Diego (UCSD) and Children’s Hospital and Health Center (CHHC). IRB Protocol Application. RESEARCH PLAN ClinicalTrials.gov; 2018 [Available from: <https://cdn.clinicaltrials.gov/large-docs/67/NCT01720667/Prot_001.pdf>. |
| Cummings JJ, Gerday E, Minton S, Katheria A, Albert G, Flores-Torres J, et al. Aerosolized Calfactant for Newborns With Respiratory Distress: A Randomized Trial. Pediatrics. 2020;146(5). <https://doi.org/10.1542/peds.2019-3967> | ONY Inc. Comparison of Aerosol Delivery of Infasurf to Usual Care in Spontaneously Breathing RDS Patients. [protocol Version 1.3]: ClinicalTrials.gov; 2020 [Available from: <https://cdn.clinicaltrials.gov/large-docs/66/NCT03058666/Prot_SAP_000.pdf>.] |
| Uchiyama A, Okazaki K, Kondo M, Oka S, Motojima Y, Namba F, et al. Randomized Controlled Trial of High-Flow Nasal Cannula in Preterm Infants After Extubation. Pediatrics. 2020;146(6). <https://doi.org/10.1542/peds.2020-1101> | Not available |
| Rakshasbhuvankar AA, Simmer K, Patole SK, Stoecklin B, Nathan EA, Clarke MW, et al. Enteral Vitamin A for Reducing Severity of Bronchopulmonary Dysplasia: A Randomized Trial. Pediatrics. 2021;147(1). <https://doi.org/10.1542/peds.2020-009985> | Rakshasbhuvankar A, Patole S, Simmer K, Pillow JJ. Enteral vitamin A for reducing severity of bronchopulmonary dysplasia in extremely preterm infants: a randomised controlled trial. BMC Pediatrics. 2017;17(1):204. <https://doi.org/10.1186/s12887-017-0958-x> |
| Holte K, Ersdal H, Eilevstjønn J, Gomo Ø, Klingenberg C, Thallinger M, et al. Positive End-Expiratory Pressure in Newborn Resuscitation Around Term: A Randomized Controlled Trial. Pediatrics. 2020;146(4). <https://doi.org/10.1542/peds.2020-0494> | Not available |
| Little EE, Cioffi CC, Bain L, Legare CH, Hahn-Holbrook J. An Infant Carrier Intervention and Breastfeeding Duration: A Randomized Controlled Trial. Pediatrics. 2021;148(1). <https://doi.org/10.1542/peds.2020-049717> | Not available |
| Gaden TS, Ghetti C, Kvestad I, Bieleninik Ł, Stordal AS, Assmus J, et al. Short-term Music Therapy for Families With Preterm Infants: A Randomized Trial. Pediatrics. 2022;149(2). <https://doi.org/10.1542/peds.2021-052797> | Ghetti C, Bieleninik Ł, Hysing M, Kvestad I, Assmus J, Romeo R, et al. Longitudinal Study of music Therapy's Effectiveness for Premature infants and their caregivers (LongSTEP): protocol for an international randomised trial. BMJ Open. 2019;9(8):e025062. <https://doi.org/10.1136/bmjopen-2018-025062> |
| Sasidharan R, Gupta N, Yadav B, Chawla D, Singh K, Kumarendu Singh A. 25% Dextrose Versus 24% Sucrose for Heel Lancing in Preterm Infants: A Noninferiority RCT. Pediatrics. 2022;149(5). <https://doi.org/10.1542/peds.2021-054618> | Not available |
| Gupta K, Amboiram P, Balakrishnan U, C A, Abiramalatha T, Devi U. Dextrose Gel for Neonates at Risk With Asymptomatic Hypoglycemia: A Randomized Clinical Trial. Pediatrics. 2022;149(6). <https://doi.org/10.1542/peds.2021-050733> | Not available |
| Treyvaud K, Eeles AL, Spittle AJ, Lee KJ, Cheong JLY, Shah P, et al. Preterm Infant Outcomes at 24 Months After Clinician-Supported Web-Based Intervention. Pediatrics. 2022;150(4). <https://doi.org/10.1542/peds.2021-055398> | Not available |
| Xu X, Yonkers KA, Ruger JP. Economic evaluation of a behavioral intervention versus brief advice for substance use treatment in pregnant women: results from a randomized controlled trial. BMC Pregnancy Childbirth. 2017;17(1):83. <https://doi.org/10.1186/s12884-017-1260-5> | Not available |
| Hayman M, Reaburn P, Browne M, Vandelanotte C, Alley S, Short CE. Feasibility, acceptability and efficacy of a web-based computer-tailored physical activity intervention for pregnant women - the Fit4Two randomised controlled trial. BMC Pregnancy Childbirth. 2017;17(1):96. <https://doi.org/10.1186/s12884-017-1277-9> | Not available |
| Bhavi SB, Jaju PB. Intravenous iron sucrose v/s oral ferrous fumarate for treatment of anemia in pregnancy. A randomized controlled trial. BMC Pregnancy Childbirth. 2017;17(1):137. <https://doi.org/10.1186/s12884-017-1313-9> | Not available |
| Duncan LG, Cohn MA, Chao MT, Cook JG, Riccobono J, Bardacke N. Benefits of preparing for childbirth with mindfulness training: a randomized controlled trial with active comparison. BMC Pregnancy and Childbirth. 2017;17(1):140. <https://doi.org/10.1186/s12884-017-1319-3> | Not available |
| Vaz JDS, Farias DR, Adegboye ARA, Nardi AE, Kac G. Omega-3 supplementation from pregnancy to postpartum to prevent depressive symptoms: a randomized placebo-controlled trial. BMC Pregnancy Childbirth. 2017;17(1):180. <https://doi.org/10.1186/s12884-017-1365-x> | Not available |
| Gomez PP, Nelson AR, Asiedu A, Addo E, Agbodza D, Allen C, et al. Accelerating newborn survival in Ghana through a low-dose, high-frequency health worker training approach: a cluster randomized trial. BMC Pregnancy and Childbirth. 2018;18(1):72. <https://doi.org/10.1186/s12884-018-1705-5> | Not available |
| Mdoe PF, Ersdal HL, Mduma ER, Perlman JM, Moshiro R, Wangwe PT, et al. Intermittent fetal heart rate monitoring using a fetoscope or hand held Doppler in rural Tanzania: a randomized controlled trial. BMC Pregnancy and Childbirth. 2018;18(1):134. <https://doi.org/10.1186/s12884-018-1746-9> | Not available |
| Olson CM, Groth SW, Graham ML, Reschke JE, Strawderman MS, Fernandez ID. The effectiveness of an online intervention in preventing excessive gestational weight gain: the e-moms roc randomized controlled trial. BMC Pregnancy and Childbirth. 2018;18(1):148. <https://doi.org/10.1186/s12884-018-1767-4> | Fernandez ID, Groth SW, Reschke JE, Graham ML, Strawderman M, Olson CM. eMoms: Electronically-mediated weight interventions for pregnant and postpartum women. Study design and baseline characteristics. Contemp Clin Trials. 2015;43:63-74. <https://doi.org/10.1016/j.cct.2015.04.013> |
| Simmons D, Nema J, Parton C, Vizza L, Robertson A, Rajagopal R, et al. The treatment of booking gestational diabetes mellitus (TOBOGM) pilot randomised controlled trial. BMC Pregnancy and Childbirth. 2018;18(1):151. <https://doi.org/10.1186/s12884-018-1809-y> | Not available |
| Daley A, Riaz M, Lewis S, Aveyard P, Coleman T, Manyonda I, et al. Physical activity for antenatal and postnatal depression in women attempting to quit smoking: randomised controlled trial. BMC Pregnancy Childbirth. 2018;18(1):156. <https://doi.org/10.1186/s12884-018-1784-3> | Ussher M, Aveyard P, Manyonda I, Lewis S, West R, Lewis B, et al. Physical activity as an aid to smoking cessation during pregnancy (LEAP) trial: study protocol for a randomized controlled trial. Trials. 2012;13:186. <https://doi.org/10.1186/1745-6215-13-186> |
| Qian XW, Drzymalski DM, Lv CC, Guo FH, Wang LY, Chen XZ. The ED(50) and ED(95) of oxytocin infusion rate for maintaining uterine tone during elective caesarean delivery: a dose-finding study. BMC Pregnancy Childbirth. 2019;20(1):6. <https://doi.org/10.1186/s12884-019-2692-x> | Not available |
| Liu JX, Shen J, Wilson N, Janumpalli S, Stadler P, Padian N. Conditional cash transfers to prevent mother-to-child transmission in low facility-delivery settings: evidence from a randomised controlled trial in Nigeria. BMC Pregnancy and Childbirth. 2019;19(1):32. <https://doi.org/10.1186/s12884-019-2172-3> | Not available |
| Charles D, Anger H, Dabash R, Darwish E, Ramadan MC, Mansy A, et al. Intramuscular injection, intravenous infusion, and intravenous bolus of oxytocin in the third stage of labor for prevention of postpartum hemorrhage: a three-arm randomized control trial. BMC Pregnancy Childbirth. 2019;19(1):38. <https://doi.org/10.1186/s12884-019-2181-2> | Not available |
| Altazan AD, Redman LM, Burton JH, Beyl RA, Cain LE, Sutton EF, et al. Mood and quality of life changes in pregnancy and postpartum and the effect of a behavioral intervention targeting excess gestational weight gain in women with overweight and obesity: a parallel-arm randomized controlled pilot trial. BMC Pregnancy Childbirth. 2019;19(1):50. <https://doi.org/10.1186/s12884-019-2196-8> | Clifton RG, Evans M, Cahill AG, Franks PW, Gallagher D, Phelan S, et al. Design of lifestyle intervention trials to prevent excessive gestational weight gain in women with overweight or obesity. Obesity (Silver Spring). 2016;24(2):305-13. <https://doi.org/10.1002/oby.21330> |
| Jose A, Mahey R, Sharma JB, Bhatla N, Saxena R, Kalaivani M, et al. Comparison of ferric Carboxymaltose and iron sucrose complex for treatment of iron deficiency anemia in pregnancy- randomised controlled trial. BMC Pregnancy Childbirth. 2019;19(1):54. <https://doi.org/10.1186/s12884-019-2200-3> | Not available |
| Asadzadeh L, Jafari E, Kharaghani R, Taremian F. Effectiveness of midwife-led brief counseling intervention on post-traumatic stress disorder, depression, and anxiety symptoms of women experiencing a traumatic childbirth: a randomized controlled trial. BMC Pregnancy Childbirth. 2020;20(1):142. <https://doi.org/10.1186/s12884-020-2826-1> | Not available. |
| Clarke M, Devane D, Gross MM, Morano S, Lundgren I, Sinclair M, et al. OptiBIRTH: a cluster randomised trial of a complex intervention to increase vaginal birth after caesarean section. BMC Pregnancy and Childbirth. 2020;20(1):143. <https://doi.org/10.1186/s12884-020-2829-y> | Clarke M, Savage G, Smith V, Daly D, Devane D, Gross MM, et al. Improving the organisation of maternal health service delivery and optimising childbirth by increasing vaginal birth after caesarean section through enhanced women-centred care (OptiBIRTH trial): study protocol for a randomised controlled trial (ISRCTN10612254). Trials. 2015;16:542. <https://doi.org/10.1186/s13063-015-1061-y> |
| de Araújo CAL, de Sousa Oliveira L, de Gusmão IMB, Guimarães A, Ribeiro M, Alves JGB. Magnesium supplementation and preeclampsia in low-income pregnant women - a randomized double-blind clinical trial. BMC Pregnancy Childbirth. 2020;20(1):208. <https://doi.org/10.1186/s12884-020-02877-0> | Alves JGB, de Araújo CAFL, Pontes IEA, Guimarães AC, Ray JG. The BRAzil MAGnesium (BRAMAG) trial: a randomized clinical trial of oral magnesium supplementation in pregnancy for the prevention of preterm birth and perinatal and maternal morbidity. BMC Pregnancy and Childbirth. 2014;14(1):222. <https://doi.org/10.1186/1471-2393-14-222>. |
| Arthur C, Di Corleto E, Ballard E, Kothari A. A randomized controlled trial of daily weighing in pregnancy to control gestational weight gain. BMC Pregnancy and Childbirth. 2020;20(1):223. <https://doi.org/10.1186/s12884-020-02884-1> | Not available |
| Seiiedi-Biarag L, Mirghafourvand M, Esmaeilpour K, Hasanpour S. A randomized controlled clinical trial of the effect of supportive counseling on mental health in Iranian mothers of premature infants. BMC Pregnancy Childbirth. 2021;21(1):6. <https://doi.org/10.1186/s12884-020-03502-w> | Not available |
| Thongchan S, Phupong V. Oral dydrogesterone as an adjunctive therapy in the management of preterm labor: a randomized, double blinded, placebo-controlled trial. BMC Pregnancy Childbirth. 2021;21(1):90. <https://doi.org/10.1186/s12884-021-03562-6> | Not available |
| Hong JGS, Tan PC, Kamarudin M, Omar SZ. Prophylactic metformin after antenatal corticosteroids (PROMAC): a double blind randomized controlled trial. BMC Pregnancy Childbirth. 2021;21(1):138. <https://doi.org/10.1186/s12884-021-03628-5> | Prophylactic Metformin After Antenatal Corticosteroids (2022). Title: Prophylactic Metformin after Antenatal Corticosteroids (Promac): A Double Blind Randomised Controlled Trial. Trial Protocol. ISRCTN. Available at: <https://doi.org/10.1186/ISRCTN10156101> |
| Hadizadeh-Talasaz F, Ghoreyshi F, Mohammadzadeh F, Rahmani R. Effect of shared decision making on mode of delivery and decisional conflict and regret in pregnant women with previous cesarean section: a randomized clinical trial. BMC Pregnancy Childbirth. 2021;21(1):144. <https://doi.org/10.1186/s12884-021-03615-w> | Not available |
| Sonoda N, Takahata K, Tarumi W, Shinohara K, Horiuchi S. Changes in the cortisol and oxytocin levels of first-time pregnant women during interaction with an infant: a randomized controlled trial. BMC Pregnancy and Childbirth. 2021;21(1):162. <https://doi.org/10.1186/s12884-021-03609-8> | Not available |
| Landry MA, Kumaran K, Tyebkhan JM, Levesque V, Spinella M. Mindful Kangaroo Care: mindfulness intervention for mothers during skin-to-skin care: a randomized control pilot study. BMC Pregnancy Childbirth. 2022;22(1):35. <https://doi.org/10.1186/s12884-021-04336-w> | Not available |
| Sarmiento I, Paredes-Solís S, de Jesús García A, Maciel Paulino N, Serrano de los Santos FR, Legorreta-Soberanis J, et al. Safe birth in cultural safety in southern Mexico: a pragmatic non-inferiority cluster-randomised controlled trial. BMC Pregnancy and Childbirth. 2022;22(1):43. <https://doi.org/10.1186/s12884-021-04344-w> | Sarmiento I, Paredes-Solís S, Andersson N, Cockcroft A. Safe Birth and Cultural Safety in southern Mexico: study protocol for a randomised controlled trial. Trials. 2018;19(1):354. <https://doi.org/10.1186/s13063-018-2712-6> |
| Veringa-Skiba IK, Ziemer K, de Bruin EI, de Bruin EJ, Bögels SM. Mindful awareness as a mechanism of change for natural childbirth in pregnant women with high fear of childbirth: a randomised controlled trial. BMC Pregnancy and Childbirth. 2022;22(1):47. <https://doi.org/10.1186/s12884-022-04380-0> | Not available |
| Ebrahimian A, Bilandi RR, Bilandī MRR, Sabzeh Z. Comparison of the effectiveness of virtual reality and chewing mint gum on labor pain and anxiety: a randomized controlled trial. BMC Pregnancy Childbirth. 2022;22(1):49. <https://doi.org/10.1186/s12884-021-04359-3> | Not available |
| Sharifi N, Bahri N, Hadizadeh-Talasaz F, Azizi H, Nezami H, Tohidinik HR. A randomized clinical trial on the effect of foot reflexology performed in the fourth stage of labor on uterine afterpain. BMC Pregnancy Childbirth. 2022;22(1):57. <https://doi.org/10.1186/s12884-022-04376-w> | Sharifi N, Bahri N, Hadizadeh-Talasaz F, Azizi H, Nezami H. The effect of foot reflexology in the fourth stage of labor on postpartum hemorrhage and after pain: Study protocol for a randomized controlled trial. Advances in Integrative Medicine. 2021;8(1):63-7. <https://doi.org/https://doi.org/10.1016/j.aimed.2020.06.004>. |
| Cabana MD, McKean M, Caughey AB, Fong L, Lynch S, Wong A, et al. Early Probiotic Supplementation for Eczema and Asthma Prevention: A Randomized Controlled Trial. Pediatrics. 2017;140(3). <https://doi.org/10.1542/peds.2016-3000> | Cabana MD, McKean M, Wong AR, Chao C, Caughey AB. Examining the hygiene hypothesis: the Trial of Infant Probiotic Supplementation. Paediatr Perinat Epidemiol. 2007;21 Suppl 3:23-8. <https://doi.org/10.1111/j.1365-3016.2007.00881.x> |
| Schwarz CE, Kreutzer KB, Langanky L, Wolf NS, Braun W, O'Sullivan MP, et al. Randomised crossover trial comparing algorithms and averaging times for automatic oxygen control in preterm infants. Arch Dis Child Fetal Neonatal Ed. 2022;107(4):425-30. <https://doi.org/10.1136/archdischild-2021-322096> | Not available |
| Foglia EE, Ades A, Hedrick HL, Rintoul N, Munson DA, Moldenhauer J, et al. Initiating resuscitation before umbilical cord clamping in infants with congenital diaphragmatic hernia: a pilot feasibility trial. Arch Dis Child Fetal Neonatal Ed. 2020;105(3):322-6. <https://doi.org/10.1136/archdischild-2019-317477> | Foglia E, Munson D, Rintoul N, Ades A, Hedrick H, Gebb J, et al. Delayed Cord Clamping for Intubation and Gentle Ventilation in Infants with Congenital Diaphragmatic Hernia: A Pilot Feasibility Trial [protocol]: ClinicalTrials.gov; 2018 [Available from: <https://cdn.clinicaltrials.gov/large-docs/33/NCT03314233/Prot_SAP_000.pdf> |
| Hunt K, Dassios T, Ali K, Greenough A. Volume targeting levels and work of breathing in infants with evolving or established bronchopulmonary dysplasia. Arch Dis Child Fetal Neonatal Ed. 2019;104(1):F46-f9. <https://doi.org/10.1136/archdischild-2017-314308> | Greenough A, Lingam I. Protocol for Optimisation of Neonatal Ventilation: Determining the appropriate level of volume guarantee for infants with evolving bronchopulmonary dysplasia. Version 3.0. Optimisation of neonatal ventilation. : ISRCTN; 2016 [Available from: <https://doi.org/10.1186/ISRCTN17041826>. |
| van der Pol LM, Tromeur C, Bistervels IM, Ni Ainle F, van Bemmel T, Bertoletti L, et al. Pregnancy-Adapted YEARS Algorithm for Diagnosis of Suspected Pulmonary Embolism. N Engl J Med. 2019;380(12):1139-49. <https://doi.org/10.1056/NEJMoa1813865> | Huisman MV, Klok FA, van der Pol LM. (2019) *Protocol for: van der Pol LM, Tromeur C, Bistervels IM, et al. Pregnancy-adapted YEARS algorithm for diagnosis*  *of suspected pulmonary embolism*. New England Journal of Medicine. 2019;380(1139-49). <https://doi.org/https://www.nejm.org/doi/suppl/10.1056/NEJMoa1813865/suppl_file/nejmoa1813865_protocol.pdf> |
| Jegathesan T, Campbell DM, Ray JG, Shah V, Berger H, Hayeems RZ, et al. Transcutaneous versus Total Serum Bilirubin Measurements in Preterm Infants. Neonatology. 2021;118(4):443-53. <https://doi.org/10.1159/000516648> | Not available |
| Lorenz L, Dawson JA, Jones H, Jacobs SE, Cheong JL, Donath SM, et al. Skin-to-skin care in preterm infants receiving respiratory support does not lead to physiological instability. Arch Dis Child Fetal Neonatal Ed. 2017;102(4):F339-f44. <https://doi.org/10.1136/archdischild-2016-311752> | Not available |
| Sien ME, Robinson AL, Hu HH, Nitkin CR, Hall AS, Files MG, et al. Feasibility of and experience using a portable MRI scanner in the neonatal intensive care unit. Arch Dis Child Fetal Neonatal Ed. 2023;108(1):45-50. <https://doi.org/10.1136/archdischild-2022-324200> | Not available |
| Lorenz L, Dawson JA, Jones H, Jacobs SE, Cheong JL, Donath SM, et al. Skin-to-skin care in preterm infants receiving respiratory support does not lead to physiological instability. Arch Dis Child Fetal Neonatal Ed. 2017;102(4):F339-f44. <https://doi.org/10.1136/archdischild-2016-311752> | Not available |
| Sharvit M, Klein Z, Silber M, Pomeranz M, Agizim R, Schonman R, et al. Intra-amniotic digoxin for feticide between 21 and 30 weeks of gestation: a prospective study. Bjog. 2019;126(7):885-9. <https://doi.org/10.1111/1471-0528.15640> | Not available |
| Belfort MA, Olutoye OO, Cass DL, Olutoye OA, Cassady CI, Mehollin-Ray AR, et al. Feasibility and Outcomes of Fetoscopic Tracheal Occlusion for Severe Left Diaphragmatic Hernia. Obstet Gynecol. 2017;129(1):20-9. <https://doi.org/10.1097/aog.0000000000001749> | Not available |
| D'Alton ME, Rood KM, Smid MC, Simhan HN, Skupski DW, Subramaniam A, et al. Intrauterine Vacuum-Induced Hemorrhage-Control Device for Rapid Treatment of Postpartum Hemorrhage. Obstet Gynecol. 2020;136(5):882-91. <https://doi.org/10.1097/aog.0000000000004138> | D’Alton ME. Clinical Investigation Plan (Protocol) PEARLE: Prospective, Single Arm, Pivotal Clinical Trial Designed to Assess the Safety and Effectiveness of the Jada™ System In Treating Primary Postpartum Hemorrhage. ClinicalTrials.gov. 2019. Available at: <https://cdn.clinicaltrials.gov/large-docs/73/NCT02883673/Prot_SAP_000.pdf> |
| Baschat AA, Rosner M, Millard SE, Murphy JD, Blakemore KJ, Keiser AM, et al. Single-Center Outcome of Fetoscopic Tracheal Balloon Occlusion for Severe Congenital Diaphragmatic Hernia. Obstet Gynecol. 2020;135(3):511-21. <https://doi.org/10.1097/aog.0000000000003692> | Not available |
| de Araújo CAL, Ray JG, Figueiroa JN, Alves JG. BRAzil magnesium (BRAMAG) trial: a double-masked randomized clinical trial of oral magnesium supplementation in pregnancy. BMC Pregnancy Childbirth. 2020;20(1):234. <https://doi.org/10.1186/s12884-020-02935-7> | Alves JGB, de Araújo CAFL, Pontes IEA, Guimarães AC, Ray JG. The BRAzil MAGnesium (BRAMAG) trial: a randomized clinical trial of oral magnesium supplementation in pregnancy for the prevention of preterm birth and perinatal and maternal morbidity. BMC Pregnancy and Childbirth. 2014;14(1):222. <https://doi.org/10.1186/1471-2393-14-222> |
| van der Zee-van den Berg AI, Boere-Boonekamp MM, Groothuis-Oudshoorn CGM, MJ IJ, Haasnoot-Smallegange RME, Reijneveld SA. Post-Up Study: Postpartum Depression Screening in Well-Child Care and Maternal Outcomes. Pediatrics. 2017;140(4). <https://doi.org/10.1542/peds.2017-0110> | Not available |
