# Supplementary material for: Patient and public involvement (PPI) reporting in maternal and neonatal clinical trials: an exploratory review
Source: Trials. 2026 Mar 6;27:300. doi: 10.1186/s13063-026-09580-z (PMC13081287; doi:10.1186/s13063-026-09580-z)
Supplement: Supplementary file 4 — Additional file 4. Characteristics of included trials (n = 352). [file 13063_2026_9580_MOESM4_ESM.docx]

Additional file 4: Characteristics of included trials (n=352)

| **Characteristics of included trials** | **Total trials (n=352)**  **n (%)** |
| --- | --- |
| **Countries** |  |
| Multi-national | 46 (13%) |
| *North America* |  |
| US | 90 (26%) |
| Canada | 10 (3%) |
| *South America* |  |
| Brazil | 6 (2%) |
| Argentina | 1 (<1%) |
| Mexico | 1 (<1%) |
| *Europe* |  |
| UK | 31 (9%) |
| Netherlands | 18 (5%) |
| France | 18 (5%) |
| Ireland | 9 (3%) |
| Sweden | 5 (1%) |
| Denmark | 5 (1%) |
| Italy | 5 (1%) |
| Spain | 3 (1%) |
| Finland | 3 (1%) |
| Germany | 3 (1%) |
| Norway | 2 (1%) |
| Switzerland | 1 (<1%) |
| *Africa* |  |
| South Africa | 3 (1%) |
| Tanzania | 3 (1%) |
| Uganda | 2 (1%) |
| Cameroon | 1 (<1%) |
| Egypt | 1 (<1%) |
| Ghana | 1 (<1%) |
| Kenya | 1 (<1%) |
| Nigeria | 1 (<1%) |
| Zambia | 1 (<1%) |
| *Asia* |  |
| India | 17 (5%) |
| Iran | 6 (2%) |
| China | 5 (1%) |
| Hong Kong, China | 1 (<1%) |
| Israel | 5 (1%) |
| Malaysia | 5 (1%) |
| Bangladesh | 1 (<1%) |
| Indonesia | 1 (<1%) |
| Japan | 2 (1%) |
| Lebanon | 1 (<1%) |
| Nepal | 1 (<1%) |
| Singapore | 1 (<1%) |
| Vietnam | 1 (<1%) |
| South Korea | 2 (1%) |
| Thailand | 3 (1%) |
| *Other* |  |
| Australia | 24 (7%) |
| New Zealand | 5 (1%) |
| **Health conditions or topic of interest investigated** |  |
| **Maternal trials** | **Total maternal trials (n=233)**  **n (%)** |
| **Antenatal conditions** |  |
| Termination of Pregnancy (TOP) | 24 (10%) |
| Miscarriage management or prevention | 5 (2%) |
| Preeclampsia | 12 (5%) |
| Gestational diabetes mellitus | 10 (4%) |
| Gestational weight gain | 8 (3%) |
| Maternal and neonatal mortality, pregnancy and birth problems | 6 (3%) |
| Hypertension | 4 (2%) |
| Stillbirth | 3 (1%) |
| Venous thromboembolism (VTE) | 3 (1%) |
| Anaemia | 2 (<1%) |
| Glycaemic control | 2 (<1%) |
| Vaginal health (biome, bacterial vaginosis) | 2 (<1%) |
| Hyperemesis gravidarum | 1 (<1%) |
| Insomnia | 1 (<1%) |
| Intrahepatic cholestasis of pregnancy | 1 (<1%) |
| Hypothyroidism or hypothyroxinaemia | 1 (<1%) |
| **Viruses and infections** |  |
| Cytomegalovirus | 3 (1%) |
| HIV infection/ complications | 3 (1%) |
| Malaria | 1 (<1%) |
| Hepatitis B Chronic infection | 1 (<1%) |
| Respiratory syncytial virus (RSV) | 1 (<1%) |
| **Fetal/neonatal-related conditions** |  |
| Foetal growth / low birth weight | 5 (2%) |
| Congenital Diaphragmatic Hernia | 3 (1%) |
| Communicable Diseases/ childhood vaccinations | 1 (<1%) |
| Cerebral Palsy | 1 (<1%) |
| Hydrops Fetalis | 1 (<1%) |
| Gastroschisis | 1 (<1%) |
| Obesity | 1 (<1%) |
| Respiratory distress syndrome | 1 (<1%) |
| **Behavioural - related conditions** |  |
| Breastfeeding | 4 (2%) |
| Pregnancy anxiety and stress/ tokophobia | 4 (2%) |
| Smoking Cessation in Pregnancy | 3 (1%) |
| Sedentary behaviour | 1 (<1%) |
| Substance abuse | 1 (<1%) |
| **Labour and birth - related conditions** |  |
| Preterm labour / birth | 14 (6%) |
| Induction of labour | 11 (5%) |
| Pain management | 10 (4%) |
| Caesarean section, complications, and use for prevention | 9 (4%) |
| Surgical/Operative birth infection | 9 (4%) |
| Fetal presentation and monitoring, complications | 8 (3%) |
| Mode of birth  *VBAC (n=2)*  *spontaneous vaginal birth (n=1)*  *Operative birth (n=1)*  *Participation in childbirth preparation classes and mode of delivery (n=1)* | 5 (2%) |
| Contraception  *Contraception (n=4)*  *Sterilisation (n=1)* | 5 (2%) |
| Cervical ripening | 4 (2%) |
| Birth injury prevention  *Obstetric anal sphincter injury (n=1)*  *Perineal injury (n=2)*  *Obstetric complication (n=1)* | 4 (2%) |
| Preterm prelabour rupture of membranes (PPROM) | 2 (<1%) |
| Place of birth | 2 (<1%) |
| Post-term birth, Prolonged pregnancy | 2 (<1%) |
| Peripartum infection | 1 (<1%) |
| Placenta accreta | 1 (<1%) |
| Placental transfusion | 1 (<1%) |
| **Postpartum interventions** |  |
| Postpartum haemorrhage | 14 (6%) |
| Postpartum depression | 4 (2%) |
| Other mental health interventions  *Post-traumatic stress disorder (PTSD) (n=2)*  *Mental health (n=1)*  *Stress, anxiety, and depression (n=1)* | 4 (2%) |
| Postnatal weight management | 1 (<1%) |
| Bonding | 1 (<1%) |
| **Neonatal trials** | **Total neonatal trials**  **(n=119)** |
| **General** | **n (%)** |
| Breastfeeding/Feeding | 8 (7%) |
| Pain management | 6 (5%) |
| Cord clamping/ cord care | 6 (5%) |
| Low birth weight | 3 (3%) |
| Neonatal Hypothermia | 3 (3%) |
| Congenital Diaphragmatic Hernia | 2 (2%) |
| Stillbirth/ Neonatal death | 2 (2%) |
| Sudden and Unexpected Postnatal Collapse | 1 (1%) |
| NICU adverse events (AE) | 1 (1%) |
| Hyperbilirubinemia | 1 (1%) |
| Prematurity- child development | 1 (1%) |
| Preterm birth - mother-infant bonding | 1 (1%) |
| Transient Hypothyroxinaemia | 1 (1%) |
| Neonatal abstinence syndrome | 1 (1%) |
| **Cardiovascular/Blood conditions** |  |
| Patent ductus arteriosus (PDA) | 3 (3%) |
| Hypoglycaemia | 3 (3%) |
| Foetal heart monitoring | 2 (2%) |
| Post haemorrhagic ventricular dilation | 1 (1%) |
| Circulatory support (blood pressure level) | 1 (1%) |
| Blood gas monitoring | 1 (1%) |
| Neonatal bloodstream infections (BSI) | 1 (1%) |
| Sepsis | 1 (1%) |
| Severe thrombocytopenia | 1 (1%) |
| **Respiratory issues** |  |
| Neonatal respiratory conditions | 38 (32%) |
| Neonatal ventilation | 6 (5%) |
| Neonatal resuscitation | 3 (3%) |
| **Neurological conditions** |  |
| Neurodevelopmental impairment (NDI) | 3 (3%) |
| Neonatal hypoxic-ischemic encephalopathy (HIE) | 3 (3%) |
| Cerebral oxygenation; | 2 (2%) |
| Cerebral Palsy | 1 (1%) |
| Neonatal seizures | 1 (1%) |
| Conditions Requiring a Brain MRI, Head Computerized Tomography, or Head Ultrasound | 1 (1%) |
| Brain injury prevention | 1 (1%) |
| **Skin conditions** |  |
| Eczema | 2 (2%) |
| Neonatal skin antisepsis | 1 (1%) |
| Skin integrity | 1 (1%) |
| **Infection** |  |
| Infection | 1 (1%) |
| Rotavirus gastroenteritis | 1 (1%) |
| **Retinal conditions** |  |
| Retinopathy of prematurity (ROP) | 2 (2%) |
| Retinal Anomalies | 1 (1%) |
