# Supplementary material for: Patient and public involvement (PPI) reporting in maternal and neonatal clinical trials: an exploratory review
Source: Trials. 2026 Mar 6;27:300. doi: 10.1186/s13063-026-09580-z (PMC13081287; doi:10.1186/s13063-026-09580-z)
Supplement: Supplementary file 5 — Additional file 5. PPI reporting in trial reports by journal and year of publication. [file 13063_2026_9580_MOESM5_ESM.docx]

Additional file 5: PPI reporting in trial reports by journal and year of publication

| **Year** | ***The BMJ***  ***(n=14)***  ***n (%)*** | ***The Lancet***  ***(n=32)***  ***n (%)*** | ***NEJM***  ***(n=42)***  ***n (%)*** | ***BJOG***  ***(n=53)***  ***n (%)*** | ***Obstetrics & Gynecology***  ***(n=82)***  ***n (%)*** | ***BMC Pregnancy & Childbirth***  ***(n=30)***  ***n (%)*** | ***Pediatrics***  ***(n=26)***  ***n (%)*** | ***Archives in Disease-Fetal & Neonatal Edition***  ***(n=48)***  ***n (%)*** | ***Neonatology***  ***(n=25)***  ***n (%)*** | **ALL JOURNALS**  ***(n=352)***  ***n (%)*** |
| --- | --- | --- | --- | --- | --- | --- | --- | --- | --- | --- |
| ***2017*** |  |  |  |  |  |  |  |  |  |  |
| Total trial reports published | 2 | 6 | 6 | 14 | 15 | 5 | 11 | 7 | 5 | 70 |
| Total trials that reported PPI | 1 (50%) | 1 (17%) | - | - | - | - | - | - | - | 2 (3%) |
| ***2018*** |  |  |  |  |  |  |  |  |  |  |
| Total trial reports published | 2 | 4 | 7 | 6 | 16 | 5 | 3 | 8 | 3 | 54 |
| Total trials that reported PPI | 1 (50%) | 3 (75%) | - | 2 (33%) | - | - | - | 1 (13%) | - | 7 (13%) |
| ***2019*** |  |  |  |  |  |  |  |  |  |  |
| Total trial reports published | 3 | 11 | 8 | 10 | 10 | 5 | 2 | 8 | 6 | 63 |
| Total trials that reported PPI | 2 (67%) | 4 (36%) | 5 (63%) | 2 (20%) | - | - | - | - | - | 13 (21%) |
| ***2020*** |  |  |  |  |  |  |  |  |  |  |
| Total trial reports published | - | 4 | 8 | 14 | 15 | 5 | 4 | 9 | 2 | 61 |
| Total trials that reported PPI | - | 2 (50%) | - | 6 (43%) | - | 1 (20%) | - | 1 (11%) | - | 10 (16%) |
| ***2021*** |  |  |  |  |  |  |  |  |  |  |
| Total trial reports published | 5 | 1 | 6 | 6 | 15 | 5 | 2 | 8 | 5 | 53 |
| Total trials that reported PPI | 2 (40%) | - | 2 (33%) | 3 (50%) | - | - | - | - | 1 (20%) | 8 (15%) |
| ***2022*** |  |  |  |  |  |  |  |  |  |  |
| Total trial reports published | 2 | 6 | 7 | 3 | 12 | 5 | 4 | 8 | 4 | 51 |
| Total trials that reported PPI | 1 (50%) | 2 (33%) | 1 (14%) | 2 (67%) | - | - | 1 (25%) | - | - | 7 (14%) |
| **Total trials reports that reported PPI** |  |  |  |  |  |  |  |  |  |  |
|  | 7 (50%) | 12 (38%) | 8 (19%) | 15 (28%) | 0 (0%) | 1 (3%) | 1 (4%) | 2 (4%) | 1 (4%) | 47 (13%) |
| The symbol ‘ – ‘ indicates ‘0’ | | | | | | | | | |  |
