# Supplementary material for: Patient and public involvement (PPI) reporting in maternal and neonatal clinical trials: an exploratory review
Source: Trials. 2026 Mar 6;27:300. doi: 10.1186/s13063-026-09580-z (PMC13081287; doi:10.1186/s13063-026-09580-z)
Supplement: Supplementary file 6 — Additional file 6. Conditions investigated in trials that reported PPI (n = 48). [file 13063_2026_9580_MOESM6_ESM.docx]

Additional file 6: Conditions investigated in trials that reported PPI (n=48)

| **Health condition/ Area under evaluation** | **Total maternal trials reporting PPI**  **(n=36)**  **n (%)** | **Proportion by total trial reporting PPI (n=48)**  **n (%)** |
| --- | --- | --- |
| ***Maternal trials*** |  |  |
| Miscarriage | 4 (11%) | 4 (8%) |
| Preeclampsia | 4 (11%) | 4 (8%) |
| Stillbirth | 2 (6%) | 2 (4%) |
| Surgical/Operative birth infection | 2 (6%) | 2 (4%) |
| Birth injury prevention | 2 (6%) | 2 (4%) |
| Caesarean section | 2 (6%) | 2 (4%) |
| Congenital Diaphragmatic Hernia | 2 (6%) | 2 (4%) |
| Maternal and neonatal mortality, pregnancy and birth problems | 2 (6%) | 2 (4%) |
| Postpartum haemorrhage | 2 (6%) | 2 (4%) |
| Mode of birth  *VBAC (n=1)*  *Spontaneous vaginal birth (n=1)* | 2 (6%) | 2 (4%) |
| Preterm birth | 1 (3%) | 1 (2%) |
| Fetal monitoring | 1 (3%) | 1 (2%) |
| Gestational diabetes mellitus | 1 (3%) | 1 (2%) |
| Induction of labour | 1 (3%) | 1 (2%) |
| Intrahepatic cholestasis of pregnancy | 1 (3%) | 1 (2%) |
| Pain management | 1 (3%) | 1 (2%) |
| Postnatal weight management | 1 (3%) | 1 (2%) |
| Postpartum depression | 1 (3%) | 1 (2%) |
| Post-term birth, Prolonged pregnancy | 1 (3%) | 1 (2%) |
| Smoking Cessation in Pregnancy | 1 (3%) | 1 (2%) |
| Traumatic childbirth – PTSD | 1 (3%) | 1 (2%) |
| Vaginal health | 1 (3%) | 1 (2%) |
| ***Health condition/ Area under evaluation*** | ***Neonatal trials that reported PPI***  ***(n=12)***  ***% (n)*** | **Proportion by total trial reporting PPI (n=48)**  ***n (%)*** |
| ***Neonatal trials*** |  |  |
| Neonatal respiratory conditions | 2 (17%) | 2 (4%) |
| Feeding | 1 (8%) | 1 (2%) |
| Anaesthesia intubation | 1 (8%) | 1 (2%) |
| Cord clamping | 1 (8%) | 1 (2%) |
| Eczema | 1 (8%) | 1 (2%) |
| Infection | 1 (8%) | 1 (2%) |
| Mother-infant bonding | 1 (8%) | 1 (2%) |
| NICU adverse events (AE) | 1 (8%) | 1 (2%) |
| Pain management | 1 (8%) | 1 (2%) |
| Patent ductus arteriosus (PDA) | 1 (8%) | 1 (2%) |
| Severe thrombocytopenia | 1 (8%) | 1 (2%) |
