# Supplementary material for: Patient and public involvement (PPI) reporting in maternal and neonatal clinical trials: an exploratory review
Source: Trials. 2026 Mar 6;27:300. doi: 10.1186/s13063-026-09580-z (PMC13081287; doi:10.1186/s13063-026-09580-z)
Supplement: Supplementary file 7 — Additional file 7. Charities/consumer organisations involved in PPI, as reported in 24 trial reports. [file 13063_2026_9580_MOESM7_ESM.docx]

Additional file 7: Charities/consumer organisations involved in PPI, as reported in 24 trial reports

|  |  |
| --- | --- |
|  | **Total %**  **(n=24)** |
| ***Country***  ***Organisation*** | **n (%)** |
| **UK** |  |
| National Childbirth Trust (NCT) | 4 (17%) |
| BLISS | 3 (13%) |
| Miscarriage Association | 3 (13%) |
| Action on Pre‐eclampsia | 2 (8%) |
| CDH UK | 2 (8%) |
| Tommy’s | 2 (8%) |
| Support for the Sick Newborn and their Parents (SSNAP) | 2 (8%) |
| AIMS | 1 (4%) |
| Birth Trauma Association | 1 (4%) |
| CHARM (Charity for Research into Miscarriage) | 1 (4%) |
| ICP Support | 1 (4%) |
| Scottish Early Pregnancy Network (SEPN) | 1 (4%) |
| Twins And Multiple Births Association (TAMBA) | 1 (4%) |
| **France** |  |
| Association SOS Prema | 1 (4%) |
| **Netherlands** |  |
| Parents’ organisation (VOC) | 1 (4%) |
| Unnamed gynaecological patients’ association | 1 (4%) |
| **Australia** |  |
| Life’s Little Treasures | 1 (4%) |
| Maternity Choices Australia | 1 (4%) |
| Running for Premature Babies | 1 (4%) |
| Stillbirth Foundation Australia | 1 (4%) |
| **Australia and New Zealand** |  |
| Perinatal Society of Australia and New Zealand (PSANZ)’s Interdisciplinary Maternal and Perinatal Australasian Collaborative Trials (IMPACT) Network | 1 (4%) |
