# Supplementary material for: Patient and public involvement (PPI) reporting in maternal and neonatal clinical trials: an exploratory review
Source: Trials. 2026 Mar 6;27:300. doi: 10.1186/s13063-026-09580-z (PMC13081287; doi:10.1186/s13063-026-09580-z)
Supplement: Supplementary file 8 — Additional file 8. The PRISMA 2020 reporting checklist. [file 13063_2026_9580_MOESM8_ESM.docx]

The PRISMA 2020 reporting checklist

|  | Item Description | Location (or reason for not reporting) |
| --- | --- | --- |
| **Title and Abstract** |  |  |
| [1. Title](https:/resources.equator-network.org/guidelines/prisma/items/title.html) | Identify the report as a systematic review. | Exploratory review – Title |
| [2. Abstract](https:/resources.equator-network.org/guidelines/prisma/items/abstract.html) | Include all items from the *PRISMA 2020 for Abstracts* checklist. | Abstract; Page 2  Not all items applicable for this review (for example, risk of bias assessment) |
| **Introduction** |  |  |
| [3. Rationale](https:/resources.equator-network.org/guidelines/prisma/items/rationale.html) | Describe the rationale for the review in the context of existing knowledge. | Background, Paragraphs 2 - 4 |
| [4. Objectives](https:/resources.equator-network.org/guidelines/prisma/items/objectives.html) | Provide an explicit statement of the objective(s) or question(s) the review addresses. | Background, paragraph 4 |
| **Methods** |  |  |
| [5. Eligibility criteria](https:/resources.equator-network.org/guidelines/prisma/items/eligibility-criteria.html) | Specify the inclusion and exclusion criteria for the review and how studies were grouped for the syntheses. | Materials and methods section, inclusion criteria |
| [6. Information sources](https:/resources.equator-network.org/guidelines/prisma/items/information-sources.html) | Specify all databases, registers, websites, organisations, reference lists, and other sources searched or consulted to identify studies. Specify the date when each source was last searched or consulted   - Specify the date when each source (such as database, register, website, organisation) wa… | Materials and methods section, ‘under search strategy’ and ‘search of journals’;  Supplementary File 1 |
| [8. Search](https:/resources.equator-network.org/guidelines/prisma/items/search.html) | Present the full search strategies for all databases, registers, and websites, including any filters and limits used   - Provide the full line by line search strategy as run in each database with a sophisticated interface (such as Ovid), or the sequence of terms that were used to search si… | Supplementary file 1 |
| [8. Selection Process](https:/resources.equator-network.org/guidelines/prisma/items/selection-process.html) | Specify the methods used to decide whether a study met the inclusion criteria of the review, including how many reviewers screened each record and each report retrieved, whether they worked independently, and, if applicable, details of automation tools used in the process. | Materials and methods section, under ‘search strategy’ and ‘search of journals’; |
| [9. Data collection process](https:/resources.equator-network.org/guidelines/prisma/items/data-collection-process.html) | Specify the methods used to collect data from reports, including how many reviewers collected data from each report, whether they worked independently, any processes for obtaining or confirming data from study investigators, and, if applicable, details of automation tools used in the process   - R… | Materials and methods section, under ‘search strategy’ and ‘search of journals’;  Page 21 (Author’s contributions) |
| 10. Data Items |  |  |
| [10a. Outcomes](https:/resources.equator-network.org/guidelines/prisma/items/data-items-outcomes.html) | List and define all outcomes for which data were sought. Specify whether all results that were compatible with each outcome domain in each study were sought (for example, for all measures, time points, analyses), and, if not, the methods used to decide which results to collect.   - List and de… | Not applicable |
| [10b. Other Variables](https:/resources.equator-network.org/guidelines/prisma/items/data-items-other-variables.html) | List and define all other variables for which data were sought (such as participant and intervention characteristics, funding sources). Describe any assumptions made about any missing or unclear information   - List and define all other variables for which data were sought. It may be sufficien… | Materials and methods section, ‘Search of journals’, paragraph 3 ; Supplementary file 2 |
| [11. Risk of bias in individual studies](https:/resources.equator-network.org/guidelines/prisma/items/risk-of-bias-in-individual-studies.html) | Specify the methods used to assess risk of bias in the included studies, including details of the tool(s) used, how many reviewers assessed each study and whether they worked independently, and, if applicable, details of automation tools used in the process.   - Specify the tool(s) (and version) u… | Not applicable - see page 8 |
| [12. Effect measures](https:/resources.equator-network.org/guidelines/prisma/items/effect-measures.html) | Specify for each outcome the effect measure(s) (such as risk ratio, mean difference) used in the synthesis or presentation of results.   - Specify for each outcome or type of outcome (such as binary, continuous) the effect measure(s) (such as risk ratio, mean difference) used in the synthe… | Not applicable – ‘Search of journals’ section, paragraph 3 |
| 13. Synthesis Methods |  |  |
| [13a. Deciding which studies were eligible for each synthesis](https:/resources.equator-network.org/guidelines/prisma/items/synthesis-methods-eligibility.html) | Describe the processes used to decide which studies were eligible for each synthesis (such as tabulating the study intervention characteristics and comparing against the planned groups for each synthesis described in item 5. | Not applicable. |
| [13b. Data preparation methods](https:/resources.equator-network.org/guidelines/prisma/items/synthesis-methods-data-preparation.html) | Describe any methods required to prepare the data for presentation or synthesis, such as handling of missing summary statistics or data conversions.   - Report any methods required to prepare the data collected from studies for presentation or synthesis, such as handling of missing summary… | Not applicable. |
| [13c. Methods for tabulating or displaying results](https:/resources.equator-network.org/guidelines/prisma/items/synthesis-methods-tabulating-or-displaying-results.html) | Describe any methods used to tabulate or visually display results of individual studies and syntheses   - Report chosen tabular structure(s) used to display results of individual studies and syntheses, along with details of the data presented. - Report chosen graphical methods used to v… | Not applicable. |
| [13d. Synthesis methods](https:/resources.equator-network.org/guidelines/prisma/items/synthesis-methods-synthesis-methods.html) | Describe any methods used to synthesise results and provide a rationale for the choice(s). If meta-analysis was performed, describe the model(s), method(s) to identify the presence and extent of statistical heterogeneity, and software package(s) used.   - If statistical synthesis methods were used… | Not applicable. Descriptive analysis was conducted. |
| [13e. Methods for exploring heterogeneity](https:/resources.equator-network.org/guidelines/prisma/items/synthesis-methods-exploring-heterogeneity.html) | Describe any methods used to explore possible causes of heterogeneity among study results (such as subgroup analysis, meta-regression).   - If methods were used to explore possible causes of statistical heterogeneity, specify the method used (such as subgroup analysis, meta-regression).   -… | Not applicable. |
| [13f. Sensitivitiy analyses](https:/resources.equator-network.org/guidelines/prisma/items/synthesis-methods-sensitivity-analyses.html) | Describe any sensitivity analyses conducted to assess robustness of the synthesised results.   - If sensitivity analyses were performed, provide details of each analysis (such as removal of studies at high risk of bias, use of an alternative meta-analysis model). - If any sensitivity an… | Not applicable. |
| [14. Reporting bias assessment](https:/resources.equator-network.org/guidelines/prisma/items/reporting-bias-assessment.html) | Describe any methods used to assess risk of bias due to missing results in a synthesis (arising from reporting biases)   - Specify the methods (tool, graphical, statistical, or other) used to assess the risk of bias due to missing results in a synthesis (arising from reporting biases). - … | Not applicable. |
| [15. Certainty assessment](https:/resources.equator-network.org/guidelines/prisma/items/certainty-assessment.html) | Describe any methods used to assess certainty (or confidence) in the body of evidence for an outcome   - Specify the tool or system (and version) used to assess certainty in the body of evidence. - Report the factors considered (such as precision of the effect estimate, consistency of f… | Not applicable. |
| **Results** |  |  |
| 16. Study Selection |  |  |
| [16a. Results of the search and selection process](https:/resources.equator-network.org/guidelines/prisma/items/study-selection-search-results.html) | Describe the results of the search and selection process, from the number of records identified in the search to the number of studies included in the review, ideally using a flow diagram | Results, paragraph 1; Figure 1. |
| [16b. Excluded studies](https:/resources.equator-network.org/guidelines/prisma/items/study-selection-excluded-studies.html) | Cite studies that might appear to meet the inclusion criteria, but which were excluded, and explain why they were excluded.   - Cite studies that might appear to meet the inclusion criteria, but which were excluded, and explain why they were excluded. | Results, paragraph 1; Figure 1. |
| [17. Study characteristics](https:/resources.equator-network.org/guidelines/prisma/items/study-characteristics.html) | Cite each included study and present its characteristics.   - Cite each included study. - Present the key characteristics of each study in a table or figure (considering a format that will facilitate comparison of characteristics across the studies). | A total of 352 trials were included in this review. This dataset is available from authors, stated under Declarations.  A table of the key characteristics of all 48 trials that reported PPI is provided in Table 4 |
| [18. Risk of bias in studies](https:/resources.equator-network.org/guidelines/prisma/items/risk-of-bias-in-studies.html) | Present assessments of risk of bias for each included study   - Present tables or figures indicating for each study the risk of bias in each domain/component/item assessed and overall study-level risk of bias. - Present justification for each risk of bias judgment—for example, in t… | Not applicable. |
| [19. Results of individual studies](https:/resources.equator-network.org/guidelines/prisma/items/results-of-individual-studies.html) | For all outcomes, present for each study (*a*) summary statistics for each group (where appropriate) and (*b*) an effect estimate and its precision (such as confidence/credible interval), ideally using structured tables or plots   - For all outcomes, irrespective of whether statistical synthesis w… | Not applicable. |
| 20. Results of Synthesis |  |  |
| [20a. Summary of studies](https:/resources.equator-network.org/guidelines/prisma/items/results-of-syntheses-summary-of-studies.html) | For each synthesis, briefly summarise the characteristics and risk of bias among contributing studies.   - Provide a brief summary of the characteristics and risk of bias among studies contributing to each synthesis (meta-analysis or other). The summary should focus only on study character… | Results paragraph 2; Table 1; Supplementary file 4 |
| [20b. Statistical results](https:/resources.equator-network.org/guidelines/prisma/items/results-of-syntheses-statistical-results.html) | Present results of all statistical syntheses conducted. If meta-analysis was done, present for each the summary estimate and its precision (such as confidence/credible interval) and measures of statistical heterogeneity. If comparing groups, describe the direction of the effect.   - Report results… | Not applicable. |
| [20c. Heterogeneity](https:/resources.equator-network.org/guidelines/prisma/items/results-of-syntheses-heterogeneity.html) | Present results of all investigations of possible causes of heterogeneity among study results.   - If investigations of possible causes of heterogeneity were conducted:   - present results regardless of the statistical significance, magnitude, or direction of effect modification. … | Not applicable. |
| [20d. Sensitivity analyses](https:/resources.equator-network.org/guidelines/prisma/items/results-of-syntheses-sensitivity-analyses.html) | Present results of all sensitivity analyses conducted to assess the robustness of the synthesised results   - If any sensitivity analyses were conducted:   - report the results for each sensitivity analysis.   - comment on how robust the main analysis was given the results of all… | Not applicable. |
| [21. Risk of reporting biases in syntheses](https:/resources.equator-network.org/guidelines/prisma/items/risk-of-reporting-biases-in-syntheses.html) | Present assessments of risk of bias due to missing results (arising from reporting biases) for each synthesis assessed   - Present assessments of risk of bias due to missing results (arising from reporting biases) for each synthesis assessed. - If a tool was used to assess risk of bias due … | Not applicable. |
| [22. Certainty of evidence](https:/resources.equator-network.org/guidelines/prisma/items/certainty-of-evidence.html) | Present assessments of certainty (or confidence) in the body of evidence for each outcome assessed   - Report the overall level of certainty in the body of evidence (such as high, moderate, low, or very low) for each important outcome. - Provide an explanation of reasons for rating down (or… | Not applicable. |
| **Discussion** |  |  |
| 23. Discussion |  |  |
| [23a. General interpretation of the results](https:/resources.equator-network.org/guidelines/prisma/items/discussion-general-interpretation.html) | Provide a general interpretation of the results in the context of other evidence   - Provide a general interpretation of the results in the context of other evidence. | Discussion, paragraphs 2-6 (Pages 16-19) |
| [23b. Limitations of included evidence](https:/resources.equator-network.org/guidelines/prisma/items/discussion-limitations-of-included-evidence.html) | Discuss any limitations of the evidence included in the review   - Discuss any limitations of the evidence included in the review. | Strengths and limitations, paragraph 2 |
| [23c. Limitations of the review processes](https:/resources.equator-network.org/guidelines/prisma/items/discussion-limitations-of-review-process.html) | Discuss any limitations of the review processes used   - Discuss any limitations of the review processes used and comment on the potential impact of each limitation. | Strengths and limitations, paragraph 1 |
| [23d. Implications](https:/resources.equator-network.org/guidelines/prisma/items/discussion-implications.html) | Discuss implications of the results for practice, policy, and future research   - Discuss implications of the results for practice and policy. - Make explicit recommendations for future research. | Discussion (pages 16-19) |
| **Other Information** |  |  |
| 24. Registration and Protocol |  |  |
| [24a. Registration](https:/resources.equator-network.org/guidelines/prisma/items/registration-and-protocol-registration.html) | Provide registration information for the review, including register name and registration number, or state that the review was not registered   - Provide registration information for the review, including register name and registration number, or state that the review was not registered. | The review was not registered. |
| [24b. Protocol](https:/resources.equator-network.org/guidelines/prisma/items/registration-and-protocol-protocol.html) | Indicate where the review protocol can be accessed, or state that a protocol was not prepared   - Indicate where the review protocol can be accessed (such as by providing a citation, DOI, or link) or state that a protocol was not prepared. | Protocol was prepared but not published. |
| [24c. Amendments](https:/resources.equator-network.org/guidelines/prisma/items/registration-and-protocol-amendments.html) | Describe and explain any amendments to information provided at registration or in the protocol   - Report details of any amendments to information provided at registration or in the protocol, noting: (*a*) the amendment itself, (*b*) the reason for the amendment, and (*c*) the stage of the… | Not applicable. |
| [25. Support](https:/resources.equator-network.org/guidelines/prisma/items/support.html) | Describe sources of financial or non-financial support for the review, and the role of the funders or sponsors in the review   - Describe sources of financial or non-financial support for the review, specifying relevant grant ID numbers for each funder. If no specific financial or non-fina… | Declarations, ‘funding’ statement |
| [26. Competing Interests](https:/resources.equator-network.org/guidelines/prisma/items/competing-interests.html) | Declare any competing interests of review authors   - Disclose any of the authors’ relationships or activities that readers could consider pertinent or to have influenced the review. - If any authors had competing interests, report how they were managed for particular review processes. | Declarations, ‘competing interests’ statement |
| [27. Availability of data, code, and other materials](https:/resources.equator-network.org/guidelines/prisma/items/availability-of-materials.html) | Report which of the following are publicly available and where they can be found: template data collection forms; data extracted from included studies; data used for all analyses; analytic code; any other materials used in the review   - Report which of the following are publicly available: templa… | Declarations ‘Availability of data and materials’ statement;  Data extraction form provided in Supplementary file 2. References of all included trials and their associated tri protocols available in Supplementary file 3. |

1. Page MJ, McKenzie JE, Bossuyt PM, Boutron I, Hoffmann TC, Mulrow CD, et al. The PRISMA 2020 statement: An updated guideline for reporting systematic reviews. PLOS Medicine [Internet]. 2021 Mar;18(3):e1003583. Available from: <https://journals.plos.org/plosmedicine/article?id=10.1371/journal.pmed.1003583>

2. Page MJ, McKenzie JE, Bossuyt PM, Boutron I, Hoffmann TC, Mulrow CD, et al. The PRISMA 2020 reporting checklist. In: Harwood J, Albury C, Beyer J de, Schlüssel M, Collins G, editors. The EQUATOR network reporting guideline platform [Internet]. The UK EQUATOR Centre; 2025. Available from: [https:/resources.equator-network.org/guidelines/prisma/prisma-checklist.docx](https://https:/resources.equator-network.org/guidelines/prisma/prisma-checklist.docx)
